# Early Infant Feeding Practices among Women Engaged in Paid Work in Africa: A Systematic Scoping Review

**DOI:** 10.1016/j.advnut.2024.100179

**Published:** 2024-01-20

**Authors:** Melina Mgongo, Scott B Ickes, Beatrice J Leyaro, Innocent B Mboya, Samantha Grounds, Emily R Seiger, Tamara H Hashim, Jamie L Conklin, Elizabeth W Kimani-Murage, Stephanie L Martin

**Affiliations:** 1 Institute of Public Health, Department of Community and Global Health, Kilimanjaro Christian Medical University College (KCMUCo), Moshi, Tanzania; 2 Better Health for the African Mother and Child, Moshi, Tanzania; 3 Department of Biological and Health Sciences, Wheaton College, Wheaton, IL, United States; 4 Kenya Medical Research Institute, Nairobi, Kenya; 5 Program in Nutritional Sciences, and Department of Health Systems and Population Health, University of Washington, Seattle, WA, United States; 6 Institute of Public Health, Department of Epidemiology and Biostatistics, Kilimanjaro Christian Medical University College (KCMUCo), Moshi, Tanzania; 7 Department of Translational Medicine, Lund University, Malmo, Sweden; 8 Department of Nutrition, Gillings School of Global Public Health, University of North Carolina at Chapel Hill, Chapel Hill, NC, United States; 9 Health Sciences Library, University of North Carolina at Chapel Hill, Chapel Hill, NC, United States; 10 African Population and Health Research Center, Nairobi, Kenya; 11 Carolina Population Center, University of North Carolina at Chapel Hill, Chapel Hill, NC, United States; 12 Department of Kinesiology and Health Sciences, William and Mary, WIlliamsburg, VA, United States

**Keywords:** breastfeeding, maternal employment, informal work, Africa, lactation support, baby-friendly workplace

## Abstract

Around the world, paid work without appropriate structural support is a key barrier to optimal breastfeeding practices. To better protect, promote, and support optimal breastfeeding practices among working women in Africa, this scoping review sought to understand how paid work influences infant feeding practices in the first 6 mo of life and what support women need to manage work and optimal infant feeding practices. We systematically searched PubMed, Scopus, Global Health, and CINAHL Plus, screened 2436 abstracts, and reviewed 322 full-text articles using Covidence for review and charting. We identified 203 articles that met the inclusion criteria. We identified 32 quantitative, 10 qualitative, 3 mixed-methods, and 2 review articles that focused on examining the relationship between work and breastfeeding, and 109 quantitative, 22 qualitative, 21 mixed-methods, and 4 review articles that included work as part of broader breastfeeding research but did not focus on work. Most studies reported a significant negative association between work and exclusive breastfeeding. Three major domains were reported in the qualitative studies: challenges to managing work and infant feeding, receiving support from employers and family members/caregivers, and strategies for feeding infants when the mother is working. Reviewed studies proposed recommendations to increase support for breastfeeding through changes to policies and support within worksites, the health system, and childcare; however, evidence of previously implemented policies or programs is limited. We recommend more consistent definitions and measurement of women’s work. Future research is needed on the impact of implementing various strategies and benefits for breastfeeding at workplaces, as well as efforts to support breastfeeding among informal workers.

**Statement of Significance**

This systematic scoping review examined the impact of maternal work on early infant feeding practices in Africa as opposed to previous studies that focused on the overall determinants of exclusive breastfeeding practices. Most studies reported a negative association between work and exclusive breastfeeding practices, suggesting an urgent need for changes to policy, worksite, health system, and childcare practices to promote optimal infant feeding practices such as exclusive breastfeeding.

## Introduction

Women identify work as 1 of the main reasons for the cessation of exclusive and continued breastfeeding [1]. Earning income can benefit families’ health, nutrition, and financial status, and paid work can also limit women’s time for infant care and feeding [2]. A meta-analysis of Demographic and Health Survey data from 50 low- and middle-income countries found that maternal employment was positively associated with the diversity of complementary foods and meal frequency from 6 to 12 mo, but not exclusive breastfeeding (EBF) for the first 6 mo [2].

Studies over several decades and global regions demonstrate time costs from household responsibilities and agricultural work, which have negative effects on infant care and feeding practices [3,[4], [148]]. Women’s workloads from paid and unpaid work continue to negatively influence breastfeeding practices [1]. Infant care and feeding responsibilities can also, in turn, limit women’s income earning abilities [5]. A review of qualitative research on EBF in sub-Saharan Africa reported that mothers commonly describe work as a barrier to EBF [6].

As urbanization increases rapidly in Africa, more women are engaging in paid work, both in the formal and informal economy [7]. The informal economy is characterized by job insecurity, lack of social protection, lower pay, and vulnerable situations [8,9]. Despite an estimated 89 % of working women in Africa working in the informal economy [10], nearly all strategies to support EBF among working mothers focus on formal workplaces [11]. Although increasingly provided by African national governments, paid maternity leave policies are typically restricted to the formal employment sector, are often for 3 mo, and typically exclude the majority of women who work without benefits in the informal sector [11,12]. The urgent need for policies and programs to support EBF for women working in the informal sector has been expressed for over 2 decades [5], but little progress has been made for this population. To better protect, promote, and support EBF among working women in Africa, a clearer understanding of how work (formal and informal) influences infant feeding practices and what support women need to successfully combine work with optimal infant feeding is needed.

This systematic scoping review aimed to answer the following 4 questions about maternal work and early infant feeding practices in Africa:

1. How does work influence infant feeding practices among women with children 0–6 mo of age?
2. What are the documented barriers and facilitators to EBF among working women?
3. What are strategies that women have used to exclusively breastfeed while working?
4. How is work defined and measured in relation to infant feeding?

For each research question, we sought to understand variations by informal and formal work.

## Methods

For this scoping review, we followed the PRISMA extension for scoping reviews (Supplemental Material) and the Joanna Briggs Institute guidelines for scoping reviews [13] to collaboratively design the protocol.

### Eligibility criteria and search strategy

We used the participants, concept, context, and studies elements [13] to evaluate eligible studies on the basis of our inclusion and exclusion criteria.

#### Participants

Participants included mothers who both reported infant feeding practices when their children were 0–6 mo of age and engaged in paid work. Paid work included formal employment, informal employment, and income-generating activities. We excluded studies conducted among women engaged in small, home-based agricultural activities as this type of work was considered outside of the boundary of paid work for this review.

#### Concept

We included studies that reported any infant feeding practices during 0–6 mo, when EBF is recommended, and that examined the relationship between work and infant feeding practices. For the purposes of the review, we use the term “infant feeding” to describe any feeding practices to children under 6 mo, and “breastfeeding” to describe the specific practice of feeding human milk. We included studies that were either “work-focused,” which explicitly sought to examine the relationship between work and early infant feeding, and “work-included,” which reported on work and infant feeding, but understanding the relationship between work and infant feeding was not a stated objective of the research. Because work is often included in studies as a sociodemographic characteristic and included as a control variable in models examining determinants of infant feeding, creating a distinction between these 2 types of studies was important when describing the literature.

#### Context

We included studies in West, Central, East, and Southern Africa, but excluded countries considered to be in North Africa and the Middle East region (i.e., Algeria, Morocco, Tunisia, Libya, and Egypt). We did not limit inclusion on the basis of other factors within study settings (e.g., rural/urban, worksites/community).

#### Studies/Source

We searched 4 databases: PubMed, Scopus, Global Health (EBSCO*host*), and CINAHL Plus with Full Text (EBSCO*host*). The last search date was 9 December 2022. We included peer-reviewed research studies with full-text articles (from online databases or authors) available in English and published during or after 1991—when indicators for assessing EBF were introduced by WHO (Greiner 2014; WHO 1991). Table 1 describes the PubMed search strategy.

**TABLE 1:** Search strategy: women, work, and breastfeeding in Africa

Search domain	Search terms
Infant feeding 0–6 mo	“infant feeding” OR “breast feeding” OR “breastfeeding” OR “breastfed” OR “breast fed” OR “breast feeds” OR “breastfeeds” OR “child feeding" OR “child nutrition" OR “infant nutrition” OR “human milk” OR “breast milk” OR “breastmilk” OR “lactation” OR “lactating” OR “lactates” OR “lactated” OR “Mixed feeding” OR “Infant formula” OR “Predominant feeding” OR “Exclusive replacement feeding” OR “Colostrum” OR “Prelacteal”
Work	“work” OR “employment” OR “working” OR “workplace” OR “workplaces” OR “worksite” OR “worksites” OR “job” OR “jobs” OR “occupation” OR “occupations” OR “occupational” OR “vocation” OR “vocations” OR “vocational” OR “employee” OR “employees” OR “employment” OR “employed” OR “personnel” OR “career” OR “careers” OR “workload” OR “traders” OR “trading” OR “informal economy” OR “informal economies” OR “sector” OR “sectors” OR “small-scale production” OR “workforce” OR “agriculture” OR “agricultural” OR “farming” OR “market” OR “markets” OR “marketplace” OR “marketplaces” OR “craft” OR “crafts” OR “mothercraft” OR “wages” OR “earnings” OR “informal labor” OR “casual labor” OR “informal labour” OR “casual labour” OR “self-employed”
Women and mothers	“women” OR “woman” OR “mothers” OR “mother” OR “maternal”
Africa	“Angola” OR “Angolan” OR “Benin” OR “Beninese” OR “Botswana” OR “Botswanan” OR “Botswanans” OR “Burkina Faso” OR “Burkinese” OR “Burkinabe” OR “Burundi” OR “Burundian” OR “Cameroon” OR “Cameroonian” OR “Cape Verde” OR “Cape Verdean” OR “Cabo Verde” OR “Central African Republic” OR “Chad” OR “Chadian” OR “Comoros” OR “Comorian” OR “Congo” OR “Congolese” OR “Cote d'Ivoire” OR “Ivory Coast” OR “Ivorian” OR “Djibouti” OR “Djiboutian” OR “Equatorial Guinea” OR “Equatorial Guinean” OR “Equatoguinean” OR “Eritrea” OR “Eritrean” OR “Eswatini” OR “Ethiopia” OR “Ethiopian” OR “Gabon” OR “Gabonese” OR “Gambia” OR “Gambian” OR “Ghana” OR “Ghanaian” OR “Guinea” OR “Guinean” OR “Kenya” OR “Kenyan” OR “Lesotho” OR “Lesothan” OR “Liberia” OR “Liberian” OR “Madagascar” OR “Madagascan” OR “Malagasy” OR “Malawi” OR “Malawian” OR “Mali” OR “Malian” OR “Mauritania” OR “Mauritanian” OR “Mauritius” OR “Mozambique” OR “Mozambican” OR “Namibia” OR “Namibian” OR “Niger” OR “Nigeria” OR “Nigerian” OR “Reunion” OR “Rwanda” OR “Ruanda” OR “Rwandan” OR “Sao Tome and Principe” OR “Santomean” OR “Senegal” OR “Senegalese” OR “Seychelles” OR “Sierra Leone” OR “Sierra Leonean” OR “Somalia” OR “Somalian” OR “South Africa” OR “Sudan” OR “Sudanese” OR “Swaziland” OR “Swazi” OR “Tanzania” OR “Tanzanian” OR “Togo” OR “Togolese” OR “Uganda” OR “Ugandan” OR “Western Sahara” OR “Zambia” OR “Zambian” OR “Zimbabwe” OR “Zimbabwean” OR “sub-Saharan Africa” OR “Africa South of the Sahara” OR “Central Africa” OR “Central African” OR “East Africa” OR “Eastern Africa” OR “East African” OR “Southern Africa” OR “South African” OR “West Africa” OR “West African” OR “Western Africa”

### Review, extraction, and synthesis

We managed the review process in Covidence Online software (https://www.covidence.org). Before beginning our review in Covidence, each team member independently read and voted on 10 abstracts to test the inclusion and exclusion criteria and ensure reviewer consistency. We discussed each abstract as a group and further refined the criteria during these discussions. Each abstract was independently reviewed by 2 team members using Covidence. We reviewed and resolved all conflicts through group discussion. For studies that passed initial screening on the basis of abstract review, the full-text articles were reviewed by 2 team members using inclusion and exclusion criteria and resolving conflicts through group discussions.

We used Covidence to manage data extraction, including country, setting, study design, sample size, participant characteristics, infant feeding measures, work measures, breastfeeding and work results, and strategies and recommendations to improve EBF among working women. We pilot tested the data extraction form as a team before extracting data. Using a spreadsheet of extracted data, we summarized studies by the following criteria: *1*) qualitative, quantitative, and mixed-methods; and *2*) work-focused and work-included studies. We summarized definitions of work and of breastfeeding and infant feeding. We then reported the relationship between work and infant feeding practices grouped by quantitative and then qualitative findings. Mixed-method studies were reported in their respective sections. We also summarized strategies and recommendations provided in the included studies and grouped these by policy, workplace, health system, enabling environment, family, and individual levels.

## Results

### Overall search results

**FIGURE 1:**
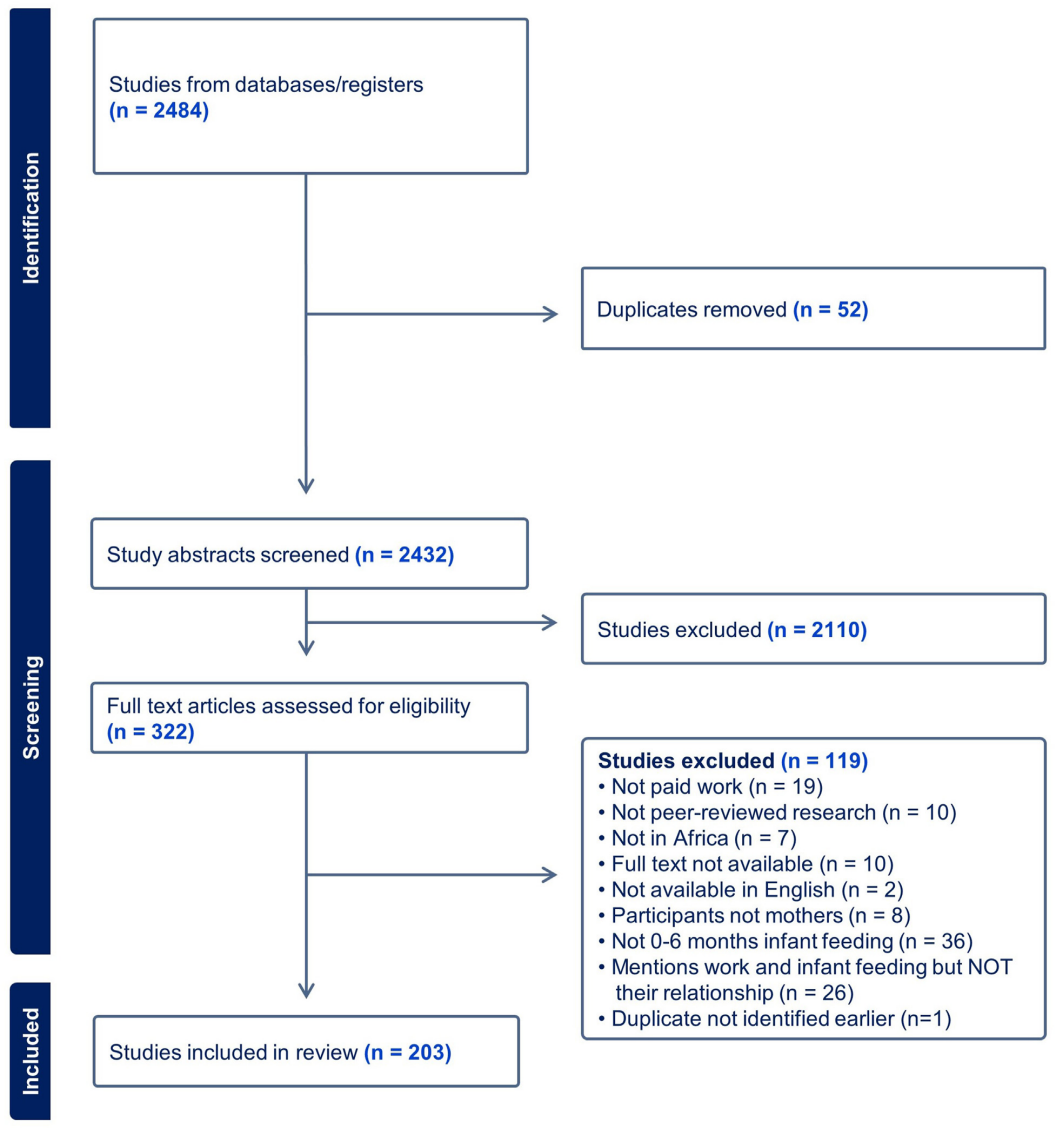
PRISMA diagram.

**FIGURE 2:**
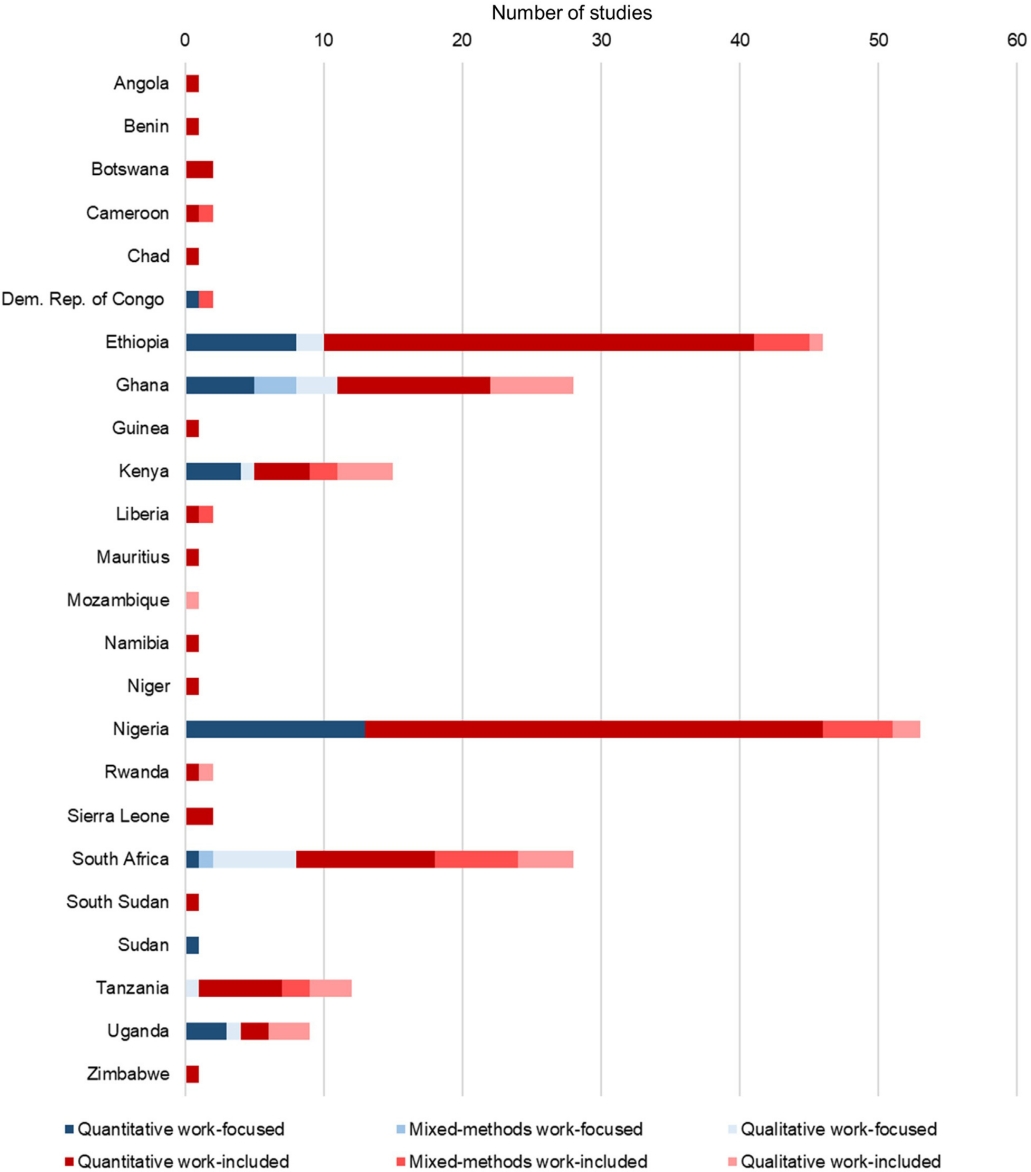
Number of studies by country and study type.

After screening 2432 abstracts and reviewing 322 full-text articles, we identified 203 articles that met the inclusion criteria (Figure 1). We identified 32 quantitative, 10 qualitative, 3 mixed-methods, and 2 review work-focused articles, 109 quantitative, 22 qualitative, 21 mixed-methods, and 4 review articles in the work-included category. Studies were conducted in 24 countries in Africa (Figure 2), with the majority based in Ethiopia, Nigeria, South Africa, and Ghana.

### Definitions of work

**FIGURE 3:**
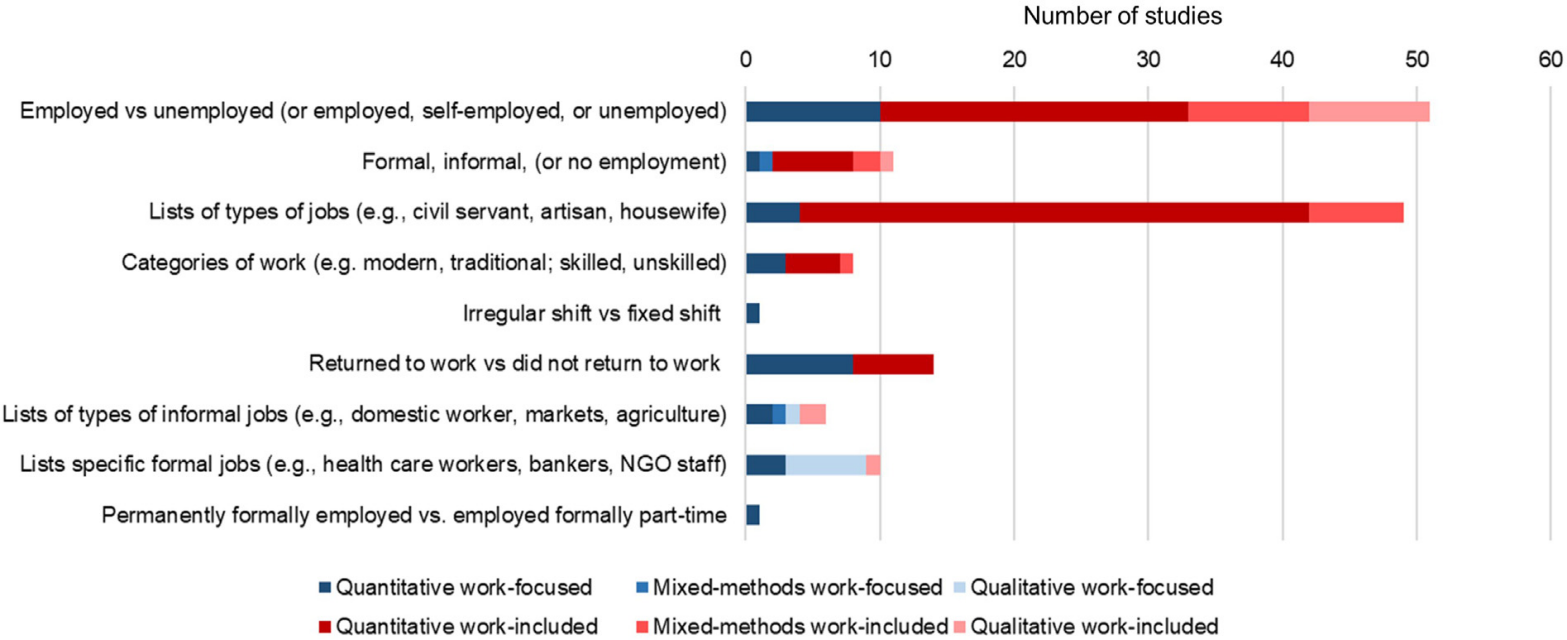
Employment categorization by study type.

Definitions of work varied across all studies (Figure 3, Supplemental Table 1). The most common approaches to measuring work were to list different types of occupations (e.g., civil servant, artisan, housewife) or to use broad categories about employment (e.g., employed, unemployed, self-employed). Although some studies discussed informal and formal work, studies rarely defined or distinguished between formal and informal work.

### Breastfeeding and infant feeding definitions and measures

Definitions of infant feeding measures varied considerably (Supplemental Table 2). Most studies consistently defined early/timely initiation of breastfeeding as infants who were put to the breast within 1 h of birth [[14], [15], [16], [17], [18], [19], [20], [21], [22], [23], [24], [25], [26], [27], [28]]. Others assessed breastfeeding initiation within 1–3 h [21]; 6 h [26]; within 24 h [23], and within 3 d postpartum [22]. Prelacteal feeding was defined as giving something other than breast milk during the first 3 d of life [[14], [29]]; feeding any drinks except medication or immunization and foods before the initiation of breast milk [30]; or feeding water, herbs, glucose, and infant formula [26]. The definition of colostrum feeding was consistent across studies as mothers fed colostrum or first milk to the infant [22,23,27,31].

The most frequently reported infant feeding measure was EBF. Most studies [17,19,28,30,[32], [33], [34], [35], [68], [149]] defined EBF on the basis of 24-h recall (24 h preceding the interviews) using the WHO/UNICEF definitions (WHO, 2021). Studies measured the prevalence of EBF at a range of time points, from 1 to 6 mo. The prevalence/proportion of EBF was different across studies on the basis of recall period (i.e., 24 h compared with time since birth or a combination of both).

Measures of non-EBF during the first 6 mo included predominant, partial, or full breastfeeding, or mixed feeding. Predominant breastfeeding was defined as infants receiving breast milk along with other liquids (e.g., water, fruit juice) and prelacteal feeds [14,[30], [32], [64]]. Full breastfeeding was defined as breastfeeding supplemented with only plain water [36]. Partial breastfeeding was defined as an infant receiving both breastfeeds and artificial feeds (e.g., either milk, or cereal, or other food) [30]. Several studies reported breastfeeding practices related to the introduction of complementary foods at 6 mo (e.g., timely initiation of complementary foods after 6 mo of EBF [24,27]; early cessation of EBF before 6 mo [37]; and age in months at the introduction of complementary foods [[26], [51]]). Mixed feeding was typically used in the context of HIV to describe feeding breastmilk along with other liquids and foods [21,25,38].

### Relationship between work and infant feeding practices

#### Quantitative studies

Table 2 summarizes the 32 work-focused quantitative studies. These studies were largely cross-sectional (30 of 32, 93.8 %) and were conducted in 8 countries. One study was conducted in Ghana and Nigeria [56], and 1 was in Nigeria and Uganda [60] for 30 study settings. Studies ranged in sample size from 36 to 5998 participants; 24 of 32 (75.0 %) enrolled a sample size of 500 or fewer participants.

**TABLE 2:**
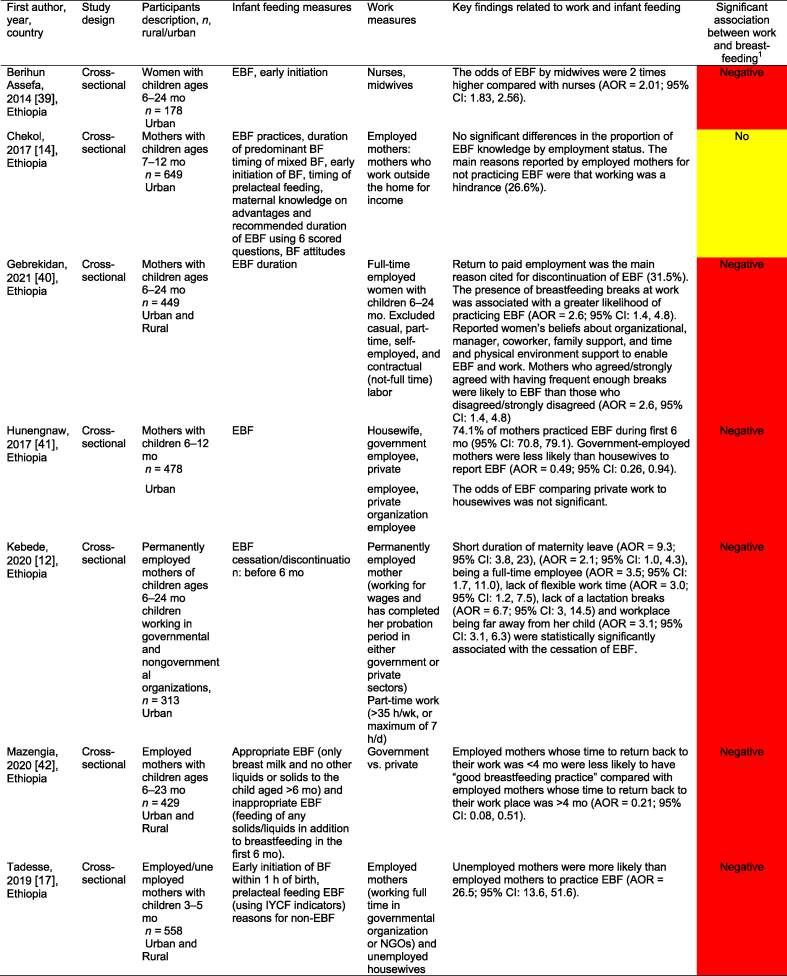

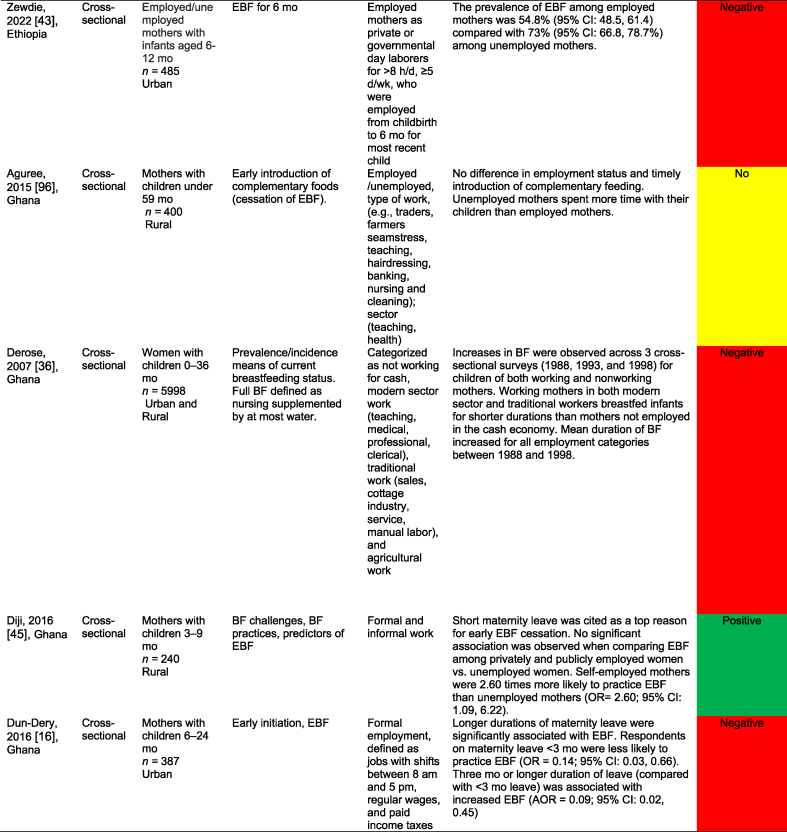

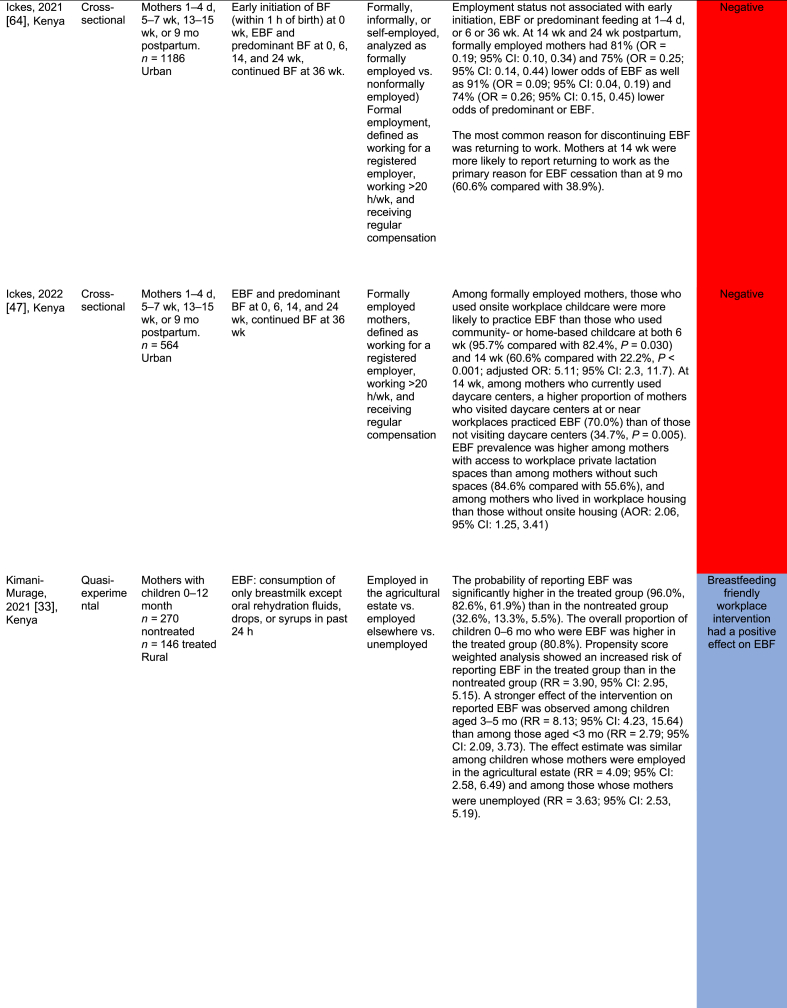

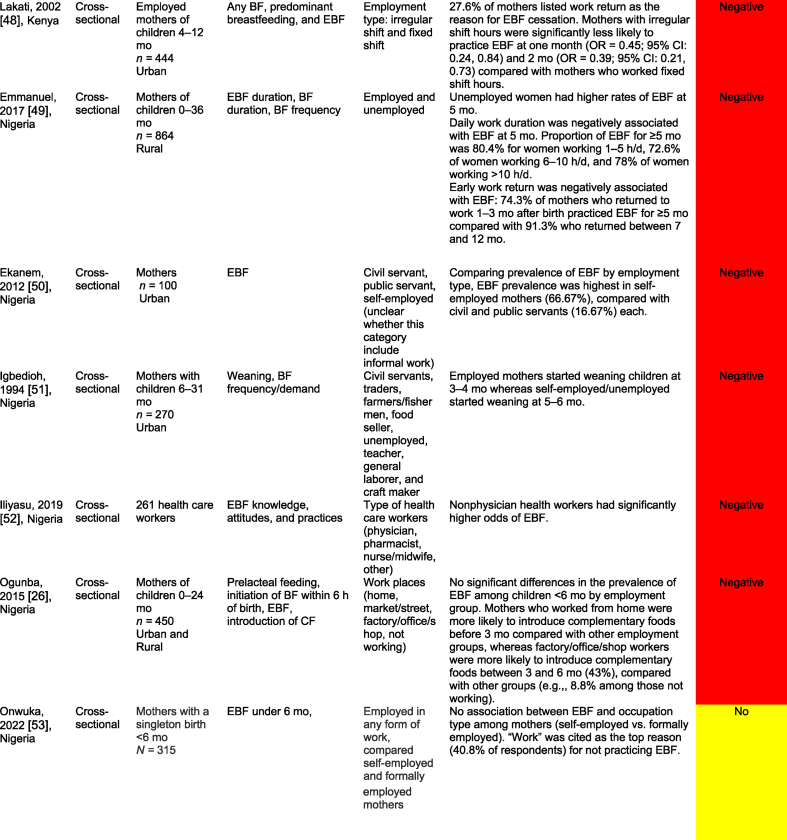

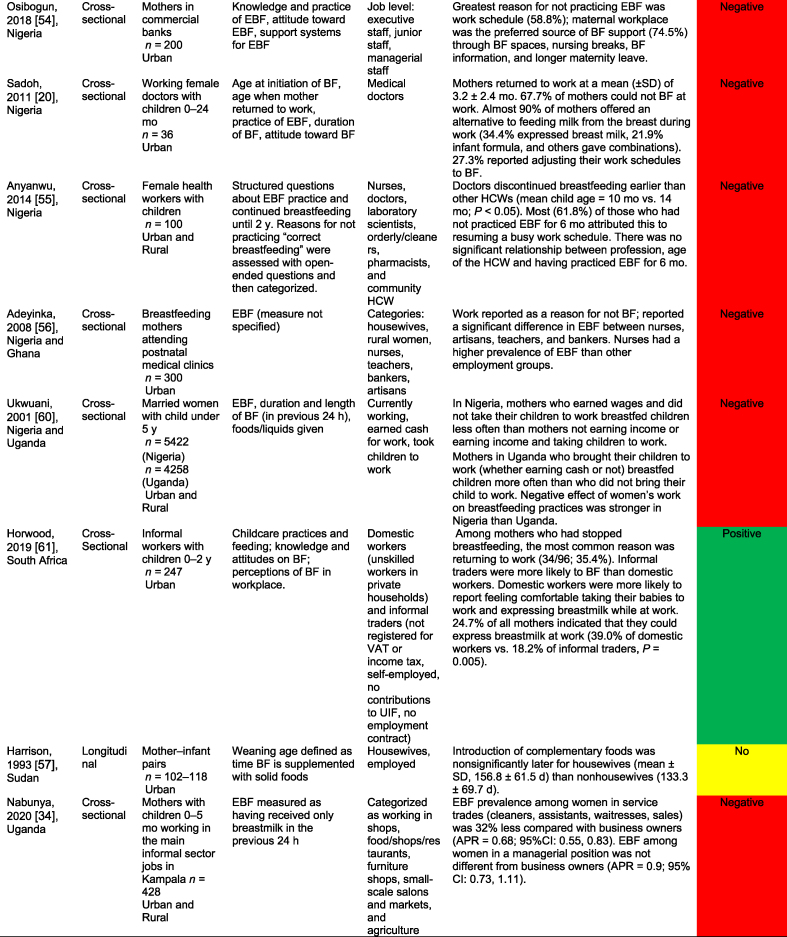

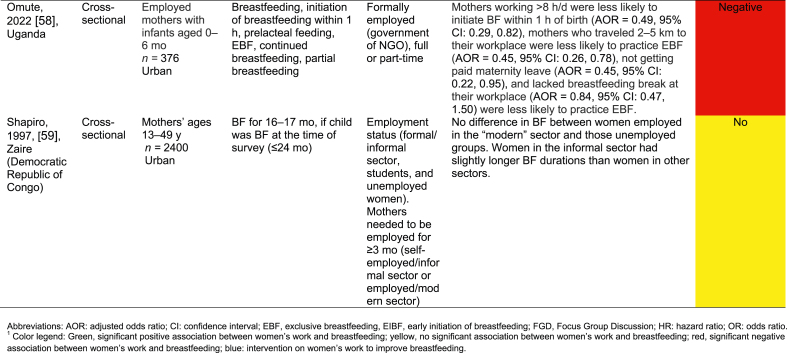
Quantitative studies focused on work and breastfeeding

A total of 27 of these 32 studies (84.3 %) reported a significant relationship between work and breastfeeding outcomes. In almost all studies (25 of 27, 92.6 %, reporting a significant relationship), the association between paid work and breastfeeding was negative because of various factors such as short duration of maternity leave, lack of breastfeeding supports (e.g., lactation breaks, breastfeeding spaces), commuting distance, and type of employment (e.g., formal, informal, or self-employed). Each of the 3 studies that reported a positive work–breastfeeding association focused on informal work and self-employment. In Nigeria, self-employed mothers were likelier than civil servants [50] to practice EBF. In South Africa, domestic workers were compared with informal traders (e.g., produce sales), noting that traders were more likely than domestic workers to breastfeed currently but less likely to report feeling comfortable taking their babies to work or expressing breastmilk at work [61]. Finally, “skilled” working mothers in Nigeria were found to be more likely to practice EBF than “unskilled” working mothers [62].

Only 1 study reported results from a workplace intervention [33]. This intervention, conducted among tea farm workers in Kenya, tested exposure to a Baby-Friendly Workplace Initiative that included onsite or community-based daycares, lactation rooms, breastmilk pumps, access to refrigeration, and mother and infant-friendly work policies. Mothers in the intervention (Baby-Friendly Workplace Initiative) group were likelier to report EBF at < 1 mo and at 5 mo of infancy than those in the control group. There was a 4-fold greater likelihood of reporting EBF over the 5-mo follow-up (RR = 3.90; 95 % CI: 2.95, 5.15), with more substantial effects observed among children in the 3–5-mo period than in those <3 mo [33].

Studies varied widely in their approaches, measurements, and comparisons. Several studies compared the likelihood of practicing EBF on the basis of the type of maternal employment (e.g., health care workers compared with other types of formal work [63]); whether they were business owners or managers [34]; and formal compared with nonformal employment [64]. Multiple surveys identified the reasons for EBF cessation, with “returning to work” as the first or second most common reason [61,64]. Three studies examined the duration of maternity leave, and noted that shorter leaves (e.g. <3 or 4 mo) were associated with a lower probability of EBF than longer leaves [12,16,42]. Similarly, not receiving any paid leave was negatively associated with EBF [58].

**FIGURE 4:**
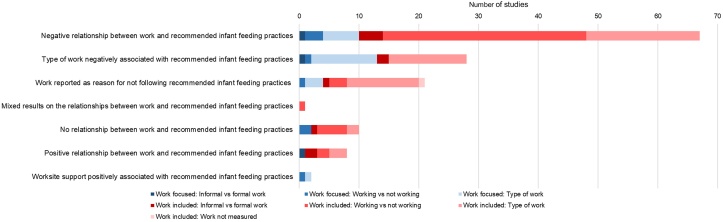
Summary of work and infant feeding relationships by study type.

Of the work-included quantitative and mixed-methods studies (*n* = 130, 109 quantitative, 21 mixed methods), 79 (60.7 %) reported a significant negative relationship between work and recommended infant feeding practices (e.g., early initiation and EBF) (Figure 4). Work was reported as a reason for not practicing optimal infant feeding practices in 17 work-included studies. Several studies reported that feeding practices differed by types of job (e.g., government employee, artisan, health care worker, domestic worker, and vendor), but the types of jobs and the relationship with infant feeding varied across studies. Infant feeding practices were also influenced by job characteristics (e.g., proximity to home, schedule, and full or part-time) (Supplemental Tables 3–5).

#### Qualitative studies

Supplemental Table 6 reports 10 work-focused qualitative studies and 22 work-included qualitative studies; Supplemental Table 4 reports 12 mixed-methods studies that reported qualitative results related to work and infant feeding (3 work-focused and 10 work-included). The qualitative results can be characterized in the following 3 main themes: *1*) challenges to managing work and infant feeding; *2*) receiving support from employers and family members/caregivers, and *3*) strategies to feed infants when the mother is working (Table 3). Mothers engaged in paid formal work reported that their workplaces had very strict working conditions, such as tight schedules, heavy workloads, and the need to resume work early, which interfered with EBF. Among studies with mothers who resumed work early, the majority reported that workplaces were not supportive of mothers to practice EBF. In some situations, work policies did not allow mothers to bring their infants to work or support breastfeeding mothers [35,37,75,[84], [93]]. In Kenya, Ethiopia, and South Africa, studies reported that the lack of a specific location to express or store breastmilk at workplaces was a barrier to EBF [64,84]. In Tanzania and South Africa, mothers were denied breastfeeding breaks during working hours, which affected EBF [65,81,94,95]. Other studies reported that mothers felt stressed while working, as they did not find time to eat or worked for long hours, which affected their ability to breastfeed [66,80,84].

**Table 3:** Synthesis of qualitative study themes, subthemes, and findings

Theme	Subthemes	Study findings
Challenges that women face to manage work and infant feeding	Long distance to work	• Long working hours away from home and long distances to work interfered with exclusive breastfeeding (EBF) [[65], [66], [67], [68], [69], [70], [71], [72], [73], [74], [101]]
	Lack of time to breastfeed while working	• Being able to bring their child to workplaces but not having time to breastfeed [66,93]
	Resuming work early	• Having to resume work early [22,28,46,63,65,75,67,70,76,77,78,79,90,[101], [136]]
	Strict working rules	• Formal employment has very strict schedules that do not allow mothers to practice EBF [73,93] • Work as stressor that affects breastfeeding, including having time to eat [66,80,84] • Some mothers reported being denied breastfeeding breaks [65,81] • Limited job security and lack of supportive policies for breastfeeding mothers in informal sector [72,82,101]
	Unsuitable workplaces conditions	• Working conditions are not supportive of EBF [40,67,79,82,86,75] • No special location to express breastmilk [46,84] • Not allowing children in their workplaces [35,73,93,[75], [84]]
Receiving support from employers and family members/caregivers	Employer support	• Receiving support from employers, like maternity benefits, helped women practice EBF [83,93] • Mothers reported to have flexibility in their workplaces to facilitate breastfeeding [66,69,81,[84], [85], [86]]
	Family support	• Caregivers feed infants when the mother was away [66,73,87,88,97,89] but mothers worried about what babies were being fed • Fathers were willing to provide support for EBF [66]
Coping mechanism to feed infants if mother is away	Breast milk expression	• Commitment to EBF and expressing breast milk [46,90,89,91,92] • Mothers had concerns that the expressed breastmilk was not sufficient or feasible to meet child needs [67,84,87,88,77,46,99] • Mothers have little control over what the child will be fed in addition to breast milk [87]
	Flexible working schedule	• Working at their homestead provided flexibility [76]
	Early introduction of other foods	• Mothers reported that early introduction of other foods such as infant formula was an alternative way to feed the child when the mother is away or if breast milk runs out [67,68,84,101,[75], [97]] • Mothers who felt they had insufficient breast milk caused by work stress used infant formula as a backup [77,88]

In most studies, participants reported receiving support for infant feeding from family members (e.g., grandmothers, spouses, older children), househelp, and employers. The family members fed infants when mothers were away for work [37,66,87,96,97]. Alhough mothers received support from other family members, 2 studies reported that mothers feared what the child was fed while the child was with another caregiver [87,96]. One study in Tanzania reported that, after tailored counseling, some fathers performed household chores and caregiving tasks to help mothers have more time to breastfeed [66]. Some studies reported on the role of the employer in supporting breastfeeding mothers, such as the provision of paid maternity leave and breastfeeding breaks during work hours [83,85,93,98] and provision of onsite childcare centers [98].

Several studies reported different maternal strategies to feed their infant when working, including breastmilk expression [40,64,[67], [77],^84^,^87^,^88^,^90^,^99^], working at home or bringing their infants to the workplace (most practical in the informal sector) [76,100], and the early introduction of other foods [68,75,84,97,67,101]. Although mothers expressed breast milk, mothers in only 3 studies reported that this could fully satisfy their infants’ needs [40,64,90]. Mothers in most studies feared that expressed breast milk was insufficient to meet children’s needs [64,[67], [77], [84], [87], [88], [99]], whereas mothers in other studies reported the perception of insufficient milk because of stress from work, affecting their ability to breastfeed [66,77,84,88,80]. In studies where women reported insufficient breast milk or mothers being away for long hours, infant formula, porridge, or cows’ milk was given to infants [[40], [68],^67^,^75^,^77^,^84^,^88^,^97^,^101^].

### Strategies and recommendations

Most articles offered recommendations to improve EBF among working women, but few reported strategies that had been observed or tested to increase EBF. Recommendations and strategies are presented in Table 4 and organized at the enabling environment, policy, workplace, health system, family, and individual levels. The most common policy-level recommendation was to extend paid maternity leave [40,42,45,61,98,105,[117], [118], [119],[133], [134], [135], [136]], but only a few articles [28,34] recommended extending protections that are available to women in formal employment to those in the informal sector. Worksite policies that support breastfeeding were also recommended by several authors, including paid breastfeeding breaks [12,61,68,136], specific spaces for breastfeeding or expression [12,61,68,118,119,124,125], and flexible working hours [12,61,125]. In addition, several authors recommended onsite or nearby childcare where mothers can breastfeed [98,105,110,112,117,119,134,136]. Counseling women about managing work and breastfeeding was recommended by several authors [61,112,113,[127], [128], [129]] as was counseling mothers on breastmilk expression [61,110,117,130]. There were fewer commonalities among the recommendations at the family and enabling environment levels. The most commonly reported strategies were bringing the infant to work [28,36,44,60,106], having flexibility with work that allowed mothers to breastfeed [12,20,33,44,48,64,85,86,105], and expressing breastmilk [12,28,77,78,90]. Armar-Klemesu et al. [5] noted that although strategies such as stopping work, working fewer hours, or bringing their infant to work can be effective in improving infant feeding practices, they can negatively impact women’s ability to earn income.

**TABLE 4:** Potential strategies and recommendations to increase exclusive breastfeeding among working mothers

Strategies tested or observed in included studies that supported EBF while working	Intervention, program, and policy recommendations from included studies
Policy • 3-mo paid maternity leave [33,86] longer maternity leave [49]; maternity leave policies [102]. • Employment protection policies for formally employed workers [102] • Child support grant received soon after childbirth for informal workers [28] Worksite • Workplace support for breastfeeding [102] • Support from employer [95] • Provide childcare facilities [103] staffed by experienced nurses [33] • Provide space [12] supplies and equipment for expressing and storing milk [33,46] • Revised existing workplace breastfeeding policy [33] • Additional employer maternity leave to complement national policies [86] • Paid breastfeeding breaks [33]/lactation breaks [12]/time to breastfeed [46]/breaks [105] • Flexible hours/schedule [[20], [33],^46^,^85^,^12^,^106^] allowed to leave work early to return home [86]/work for a short time return to child [44] work <8 h/d [105] • Support from coworkers [28,95] Health system • Training on breastmilk expression [107] Family • Husband [42] and husband and family support [40,85] Individual • Delay return to work until 6 mo [28,77,] stop working [101] • Combining annual leave with maternity leave to extend [86] • Bring infant to work [28,36,60,44,48] • Change working schedule [28] • Return home to the infant every few hours [71] • Expressing breast milk [12,28,[77], [78],^90^,^89^] • Express and store breastmilk to build a supply before returning to work [95] • Breastfeed more at night while working during the day [108]	Enabling environment • Breastfeeding promotion specific to employed mother [46] include messages about work and EBF in breastfeeding campaigns [109] acknowledge challenges of working women [96] and mother-centered benefits of BF [110] • Address social norms [46,101] • Promote breastmilk expression [22,34] • Increase community and family awareness on the importance of breastfeeding within the work environment [111] • Adapt interventions for working vs. nonworking [14] • TV shows with mothers of different professions BF, mass media [112] • Efforts to create enabling environment specific for employed mothers [113] Policy • Legislation [46], national policies [114,115] and programs for working mothers [114] in all workplaces [12] • Implement existing national policies [112] and create policy guidelines with details for action [89] • Paid maternity leave [116,136] • Extend maternity leave policies [42,45,78,[117], [118],[133], [134], [135]] to 6 mo [40,105,119] • Extend protections available to formal sector to informal sector [34] • Policies for women in the informal sector [5] • Governments should provide maternity grants to women in the informal work environment [28] • Policy makers should consider working mothers [35,120] • Cash transfers for informal workers immediately after childbirth [28] Worksite • Create EBF friendly worksites [33,73,116] • Policies at workplaces to support women to combine work with breastfeeding [24,33,46,49,63,121] • Increase awareness of existing related policies [34,37] • Employers provide longer maternity leave [41] • Employer-based program to support EBF [33,46,49,68,122] • Promote EBF at worksites [123] • Paid breastfeeding breaks [12,61,68,136] time for breastfeeding for employees [123,124] • Specific place for breastfeeding/expression [12,33,43,46,61,68,118,119,124,125] • Flexible work hours [12,61,100,103,104,125] • Shorter duration of work [12]/part-time work arrangements [136] • Provide childcare [43], onsite childcare [112,117,119] where mothers can breastfeed [105] or nearby childcare [110,134,136] • Improve childcare options near workplaces [22] • Allow mothers to bring their children to work [100] or have children brought to them at feeding time [104] • Increase support from coworkers and supervisors [12] by increasing awareness of importance of EBF [28,61] • Mother-friendly work places facilities for the child to stay with the mother safely adapted to informal settings.[61] • Sensitize employers in informal sector of the benefits of EBF and the existing laws and policies [34] • Allow mothers who live close to go home to breastfeed [104] • Provide safe transport [126] Health system • Counseling about work and BF [61,112,113,127,128,129] • Health care workers need skills to support working mothers [78] • Counsel mothers about breastmilk expression [110,111,117,130] • Interventions that promote EBF among HCWs [39,55,131] • Peer or health worker BF counseling [46] Family • Involve other caregivers and family members [85,61] • Encourage fathers to come with mothers to child health visits to advise parents on the importance of delaying income-generating activities for EBF [76] • Increased support to decrease mothers’ workload and increase time for breastfeeding [132]

## Discussion

This scoping review comprehensively examines the relationship between maternal work and early infant feeding practices in Central, East, Southern, and West Africa. This study adds to the body of knowledge on how paid work influences EBF practice in Africa and highlights the lack of support for lactating women working in different sectors. Our findings indicate a consistent, primarily negative, relationship between paid work and EBF, similar to findings from other regions [1]. However, the heterogeneity of studies, especially around the measurement of work and outcome indicators of breastfeeding, makes study comparison challenging and precludes our ability to produce an effective estimate of the impact of work on breastfeeding. Only 1 study tested an intervention to improve EBF among working mothers, but qualitative studies identified individual, family, health system, worksite, and policy-level actions that promote and support EBF. With >60 % of women of childbearing age engaged in paid work in Africa, the highest participation rate of any region [137], understanding the relationship between work and EBF is of public health importance. The timing of this review is essential as several African nations are in the policy planning or implementation stages to promote, protect, and support breastfeeding among employed mothers [138].

National policies and worksite programs can improve EBF rates among working women [1,116]. However, strategies to support EBF for working women typically focus on policies and benefits for formally employed women and programs at formal worksites [139], despite 9 out of 10 women engaged in paid work in Africa working in the informal sector [9]. Women in the informal sector face unique breastfeeding challenges, financial insecurity, unsuitable working situations, and low wages. These women do not benefit from national maternity protection policies, often requiring them to resume work soon after birth. All these challenges interrupt EBF practice and negatively affect maternal and child health and nutrition. In most African countries, few policies exist to protect women in the informal sector. Where maternity protection policies exist, they are often weak, and many women lack adequate knowledge to benefit from their provisions [11]. The International Labour Organization has proposed maternity cash benefits for women working in the informal sector [140] and cash transfers have been recommended for informally employed women [116]. However, maternity benefits for women working in the informal sector are currently lacking in most countries [12]. A child support grant is available to low-income mothers working in the informal sector in South Africa. However, delays in receiving funds impair breastfeeding practices as women resume work [28]. Future research using consistent measures of work that include formal and informal work categories, as well as work schedules, locations, and whether the infant is with them, is needed to understand better how work influences breastfeeding. Although in some settings, women who work informally may have higher rates of EBF than women who work in the formal sector, it is difficult to understand these relationships without clear and standard employment/work measures. For example, there were differences in breastfeeding practices among domestic workers and hawkers in South Africa, both informal workers [61].

An analysis of maternity leave duration on breastfeeding practices in 38 LMICs identified that a 1-mo increase in the legislated duration of paid maternity leave was associated with a 7.4 % increase in the prevalence of early initiation and a 5.9 % increase in the prevalence of EBF, among infants under 6 mo, corresponding to a 2.2-mo increase in BF duration [141]. An analysis by the World Bank indicates that, besides South Africa, Ethiopia, and the Gambia, most African countries only provide between 12 and 14 wks of leave (World Bank Gender Data Portal).

The data sources on the work–breastfeeding topic are mainly derived from 5 countries (Ethiopia, Ghana, Kenya, Nigeria, and South Africa) and are skewed to English language publications. Further, few multicountry settings use consistent methodology across different contexts. However, the reviewed studies demonstrate an overwhelmingly consistent relationship: paid work challenges EBF. Among populations of mothers who receive maternity leave, the impact of paid work and employment on breastfeeding is most substantially observed after mothers return to work [64]. Among mothers, those in management positions and business owners experience better breastfeeding outcomes than those engaged in wage labor [98]. These advantages might be because of having greater awareness of the policies, increased flexibility, and more income/financial security that does not require a premature return to work. Informal and self-employed mothers—those working in trades such as cleaning, food service, and market sales—are uniquely vulnerable to the challenges of work throughout the lactation period because of the lack of maternity protection policies.

Most studies recommended strategies to protect, promote, and support breastfeeding, but this review only identified one study in Africa that tested an intervention to improve breastfeeding in the workplace. In a global systematic review of worksite interventions to improve breastfeeding [116], the most common strategies were having a designated private space for breastfeeding or expressing milk and support from supervisors or coworkers, followed by flexible schedules to support milk expression during work and written breastfeeding policies. Most studies were from North America and East Asia, and the Pacific, and included other high- and middle-income countries, but none from Africa [116]. A realist review examining components of effective workplace interventions in 11 countries drawn mainly from North America noted the importance of supervisor and coworker awareness, support, and time to breastfeed or express during work [142]. Similarly, a systematic review and meta-analysis of studies in the United States and Asia found that workplace interventions, such as group education, support groups, and lactation spaces, can improve breastfeeding outcomes [143]. These reviews highlight the need for tailored interventions for women working in the informal sector.

Differences in the conceptualization or measurement of paid work across studies limit our ability to compare relationships between work and infant feeding practices. Most quantitative studies were cross-sectional and could not examine the temporal association between employment and breastfeeding. Despite the variation in the work measurement, these studies consistently agree on the negative relationship between paid work and breastfeeding practices. An analysis of the relationship between maternal work and infant feeding measures highly depends on the research question or objective. Detailed definitions and careful analysis of the maternal paid work and infant feeding relationship should reflect the local context to inform interventions, such as the predominant types of women’s work, the political capacity for legislated workplace supports, and existing maternity services provided by employers.

To strengthen the existing evidence base on maternal paid work and infant feeding in Africa, we recommend longer, prospective epidemiologic studies among working mothers in various settings that include both formal and informal work and use standardized measures of paid work. The current evidence base relies heavily on cross-sectional studies, which risks reverse causal inference whereby women of higher socioeconomic status may be less likely to resume paid work after delivery and where mothers of lower status may have fewer options to delay the return to paid work. Second, few studies have evaluated interventions to improve breastfeeding support for working mothers [33]. Evaluations of interventions are needed to identify the most beneficial policies and supports for breastfeeding promotion. This line of research could also examine women's productivity and how it is affected by mother and infant-friendly work policies, as well as a cost–benefit analysis of implementing these policies. Third, additional resources are needed for state-led social protection measures for women working in the informal sector, as these mothers do not benefit from the same maternity protection policies directed toward formally employed women. Fourth, more consistency is needed in measuring and defining work for research and policy purposes. The current evidence base makes comparison across studies difficult. Considerations should be made to differentiate contracted employment with benefits, self-employment, and informal employment. The benefits, if any, that are available to self- and informally employed women should also be reported in analyses.

Most research about work and infant feeding focuses on mothers’ perspectives and experiences. Future research should include key stakeholders (e.g., employers, managers, supervisors, other caregivers, and policymakers), especially in qualitative and implementation research. There are few examples of studies that have explored managers’, supervisors’, or employers’ perspectives to support EBF at workplaces [[40], [46], [78], [98], [103], [104]]. Moreover, it was evident that they play a prominent role in implementing the policies that support EBF. Engaging these influential groups in research can provide evidence of willingness to support EBF; however, there is a gap in understanding what workplaces are willing to implement and support. One-size-fits-all policies and programs are likely insufficient, and worksite-specific policies must be created and implemented in close collaboration with managers.

This review identified several studies examining workplace chemical exposures and chemical concentrations in breastmilk. These studies did not report on infant feeding practices and did not meet the inclusion criteria. However, more research is needed to describe chemical exposures from worksites, to understand how exposure to chemicals (e.g., flower farms/greenhouses) impacts infant feeding practices and maternal and child health, and to identify ways to limit women’s workplace chemical exposures [144,145,146].

This review calls for formulating or strengthening policies supporting exclusive and continued breastfeeding for working women in Africa. Given that the available policies favor the formal economy, these strengthened policies should ensure that women working in the informal economy have access to maternity protection. Strategies to support the women working in the informal economy may include advocacy for and actions toward fair wages, regulatory enforcement, improved governance, enhanced safety nets, and the inclusion of relevant stakeholders that reflect this population’s unique needs and priorities [147].

### Limitations

This review represents the most comprehensive synthesis of studies on work and early infant feeding practices in Africa. Our review was limited by including articles published only in English, which may have excluded some articles published only in French or Portuguese. A review of the gray and peer-reviewed literature about factors that influence EBF in Central and West Africa included research in French that reported that informal and formal work is a critical barrier to EBF and offered recommendations similar to those from our review. Second, our search may have missed other studies that measured but did not report work in the abstract. Our review was strengthened by using a clearly defined search protocol implemented by an independent librarian and a conflict resolution process to ensure that article inclusion criteria and data extraction were consistently applied.

## Conclusion

Maternal paid work represents an important source and result of women’s empowerment. However, robust evidence from 3 decades indicates a consistent and negative association between women’s work and breastfeeding in Africa and limited evidence of effective workplace interventions. Although most African countries meet the ILO recommendation of 14 wk of paid maternity leave, this policy needs to enable women to practice EBF for the recommended 6-mo duration and systematically excludes women in the informal sector. More efforts in policy, programming, and research in the formal and informal sectors are needed to ensure that women are well-supported to exclusively breastfeed and engage in paid work so that the roles of mothering and working outside the home successfully co-exist.

## Author contributions

The authors’ responsibilities were as follows –MM, SLM, SG, BL, IBM, ERS, TH: designed the study; MM, SLM, SG, BL, IBM, SBI, ERS, TH: performed the literature review and data synthesis; and all authors: read and approved the final manuscript.

## Conflict of interest

The authors report no conflicts of interest.

## Funding

SLM received support from NICHD of the National Institutes of Health under award number P2C HD050924 and from the International Society for Research on Human Milk and Lactation (ISRHML)-Family Larsson-Rosenquist Foundation Trainee Expansion Program. SBI received support from NIH under award number 3K01TW010827-05S1.

## Data availability

Data for this review will be made available upon written request to the corresponding author.

## Appendix A Supplementary data

Multimedia component1 Multimedia component1
