# Supplementary material for: Early Infant Feeding Practices among Women Engaged in Paid Work in Africa: A Systematic Scoping Review
Source: Adv Nutr. 2024 Jan 20;15(3):100179. doi: 10.1016/j.advnut.2024.100179 (PMC10877690; doi:10.1016/j.advnut.2024.100179)
Supplement: Multimedia component1 [file mmc1.docx]

**Supplemental Material**

**Supplemental Table 1**. Work measurements and definitions

| **Work measure** | **Type of study** |
| --- | --- |
| **Formal and informal work** | |
| Categorized as employed or unemployed, or employed, self-employed or unemployed (includes housewives. Vs. employed) | - Quantitative: Work-focused^1–9^ - Quantitative: Work and BF reported but not focus^10–32^ - Mixed Methods: Work and BF Reported but not focus^33–40^ - Qualitative: Work and BF reported but not focus^17,41–48^ |
| Categorized as formal or informal (or no employment) | - Quantitative: Work-focused^49^ - Mixed Methods: Work-focused^50^ - Quantitative: Work-included^51–56^ - Mixed Methods: Work and BF Reported but not Focus^57,58^ - Qualitative: Work and BF reported but not focus^59^ |
| Listed different types of jobs (e.g. civil servant, artisan, housewife, self-employed) | - Quantitative: Work-focused^60–63^ - Quantitative: Work and BF reported but not focus^12,64–98^ - Mixed Methods: Work and BF Reported but not Focus^99–105^ |
| Listed different categories or sectors of work (e.g. modern, traditional, government, NGO; white collar, blue collar, not working; skilled, unskilled) | - Quantitative: Work-focused^106–108^ - Quantitative: Work and BF reported but not focus^109–112^ - Mixed Methods: Work-included^113^ |
| Categorized as irregular shift vs fixed shift | - Quantitative: Work-focused^54^ |
| Returned to work vs. did not return to work (formal or informal not specified) | - Quantitative: Work-included^114–121^ - Qualitative: Work and BF reported but not focus^122–127^ |
| Type of work not specified but work (and related activities) discussed in qualitative results | - Mixed Methods: Work-included^128–132^ |
| **Informal work only** | |
| Specific types of informal work (e.g. domestic workers, working in shops, food, restaurants, furniture shops, salons, markets, agriculture, traders) | - Quantitative: Work-focused^133,134^ - Mixed Methods: Focused on Work and BF^135^ - Qualitative: Focused on Work and BF^116^ - Qualitative: Work and BF reported but not focus^59,136^ |
| **Formal work only** | |
| Lists specific formal jobs (e.g. health care workers, bankers, NGO staff) | - Quantitative: Work-focused^137–139^ - Qualitative: Focused on Work and BF^140–145^ - Qualitative: Work and BF reported but not focus 2019^146^ |
| Categorized as permanently employed vs. employed part-time | - Quantitative: Work-focused^147^ |

**Supplemental Table 2**. Breastfeeding and infant feeding definitions and measures used in included articles

| **Feeding practice** | **Infant feeding measures** |
| --- | --- |
| Exclusive breastfeeding (EBF) | EBF: feeding only breast milk (including milk expressed or from a wet nurse) without anything else in the last 24 h preceding the interview except for ORS, drops, and syrups (vitamins, minerals, medicines) for therapeutic purposes ^10,34,51,65,79,103,134,152,159^  EBF: measured according to the WHO/UNICEF definitions for assessing infant and young child feeding (IYCF); based on the maternal 24 h recall ^51^  EBF rate: "proportion of infants less than 120 days of age who were exclusively breastfed in the last 24 hours".^65^  EBF at one and six wk ^38^, one, three, and six m ^54^. EBF for children 0-5 m ^35^.  EBF: feeding breastmilk only with no other solids from childbirth through the time point of data collection) at 0, 6, 14, and 24 wk ^142^.  EBF: Exclusive breastfeeding (nothing but breast milk) measured by asking mothers if they initiated EBF for the baby and to state the duration of EBF in m. (mothers who breastfed infants exclusively for 1 to 3 wk were coded as 1 month) ^160^.  EBF: proportion of 0-5.9 m old infants who were exclusively breastfed ^15^.  Appropriate EBF practice: feeding only breast milk and no other liquids or solids, even water, with the exception of medicines prescribed by health professionals to the child age less than six m of age. Inappropriate EBF practice is feeding of any solids/liquids in addition to breastfeeding in the first six m of age of infants ^107^.  EBF cessation/discontinuation: A mother stopping/interrupting exclusive breastfeeding her child before 6 m since delivery according to WHO recommendation for EBF (feeding only breast milk, and no other liquids or solids with the exception of oral rehydration solution, supplements or medicines to the child age less than 6 m since birth) ^147^.  Age (of the child) at cessation of breastfeeding^35^ |
| Feeding colostrum | Whether the child was given colostrum or not^57,66,68,130^ |
| Early initiation | Early/timely initiation of breastfeeding: infants who were put to the breast within one hour of birth^2,35,51,57,64,68,99,108,130,135,138,148,150,156, 159,163^  Initiation of breastfeeding within 1-3 hours^99^ and six-hour of birth^108^  Breastfeeding initiation after birth (included, within 1 and 24 hours, and 3 days after delivery) ^130^, within 24 hours ^57^. |
| Prelacteal feeding | Prelacteal feeding: children who have been given something other than breast milk during the first three days of life^2,162^  Prelacteal feeding: giving infants any drinks except medication or immunization and foods before the initiation of breast milk^10^  Prelacteal feeding: water, herbs, glucose, infant formula^108^ |
| Giving other foods or liquids before 6 months | Complementary feeding: defined as any liquid or solid food given to the child besides breast milk. This did not include prescribed medications and supplements by doctors.^1,35,54,63,108,150,153^  Complementary feeding with continued breastfeeding and complementary feeding with no breast milk^35^  Timely initiation of complementary feeding (after 6 m of EBF)^68,150^.  Complementary feeding (liquids): using bottle, cup feeding, and solid foods, particularly maize porridge^153^  Age of introduction of complementary food^108^, or age in m when supplementary food was started^6^  Partial breastfeeding: Giving a baby some breastfeeds, and some artificial feeds, either milk or cereal, or other food^10^  Early introduction of complementary/supplementary feeding: introducing foods or liquids while breastfeeding before six m^42^  Time (in m) of introduction of extra foods in addition to breastfeeding (within) first 6 m^163^  Age at initiation of cereals^161^ |
| Predominant breastfeeding | Predominant breastfeeding: the practice of feeding breast milk along with water ^2^  Predominant breastfeeding: “the infant's predominant source of nourishment has been breast milk. However, the infant may also have received water and water-based drinks; fruit juice; oral rehydration salts solution; drop and syrup forms of vitamins, minerals and medicines; and ritual fluids in limited quantities. With the exception of fruit juice and sugar-water, no food-based fluid is allowed under this definition” ^65^  Predominant breastfeeding: The infant’s predominant source of nourishment has been breast milk, including milk expressed or feeding from a wet nurse as the predominant source of nourishment. However, the infant may also have received other liquids (water, water-based drinks, fruit juice), ritual fluids and ORS, drops or syrups (vitamins, minerals and medicines)^10^.  Predominant breastfeeding rate: proportion of infants less than 4 m of age who were predominantly breastfed in the last 24 hours^65^  Predominant breastfeeding at 0, 6, 14, and 24 wk^142^  Full breastfeeding: nursing supplemented by at most plain water^106^ |
| Mixed feeding | Mixed breastfeeding: the practice of feeding breastmilk along with other foods, liquids^14,18,27,35,40,41,70,99,115,116,135,169^  Mixed feeding of children 0-5 m^35^  Mixed feeding: [information on infant feeding practices and on the reasons for choosing different modes of infant feeding was obtained at 1 and 6 wk. At these visits, women were asked how they were currently feeding their infants (previous 24 hours)] ^38^ |
| Replacement feeding | Replacement feeding (Yes/ No)^163^  Replacement feeding at six wk ^38^and children 0-5 m^35^ |
| Continued breastfeeding | Continued breastfeeding: breastfeeding until 24 m (2 y)^68,114,161^  Continued BF at 36 wk^142^ |
| Breastfeeding status of all children <3y | Current breastfeeding status of all children born in the three y before the survey^106^ |
| Breastfeeding performance | Breastfeeding performance index (BPI) scores: assessed positive BPI scores of 1 point each for seven healthy breastfeeding practices^80^ |
| Weaning | Weaning age:  the time in days when breastfeeding begins to be regularly supplemented with solid food^5,6^ |
| Not breastfeeding | No breastfeeding^40^ |
| Breastfeeding intention/ decisions | The decision to exclusive breastfeeding - compared to exclusive formula feeding (one variable with the two categories)^33^  Intention to breastfeed for pregnant women (plans to breastfeed vs. plans not to breastfeed)^132^ |

**Supplemental Table 3**. Quantitative studies that included but were not focused on employment and breastfeeding

| **Author, Year, Country** | **Study Type** | **Population** | **Infant Feeding Measurement** | **Employment measurement** | **Findings that related work and breastfeeding** |
| --- | --- | --- | --- | --- | --- |
| 1 Adewuyi, 2017, Nigeria^64^ | Cross-sectional | Mothers with children 0-24 m, n=11851 | Early initiation of BF | Formal and informal work: Not working, agriculture and paid work | Association between work and early initiation of BF. Odds of BF was greater among non-working mothers or those engaged in household works. |
| 2 Adugna, 2017, Ethiopia^10^ | Cross-sectional | Mothers with infants 0-6 m, n=529 | EBF, BF, Partial BF, Prelacteal Feeding | Housewife vs. employee | Crude OR: Housewives had 2.15 times the odds of EBF compared to employees: (95% CI: 1.5, 3.07). AOR: Housewives had 2.6 times the odds of EBF compared to employees. (95% CI: 1.34, 4.9). |
| 3 Aghaji, 2002, Nigeria^65^ | Cross-sectional | Mothers with children <120 days old, n=235 | EBF; EBF rate (proportion of infants exclusively BF in last 24 hours); predominant breastfeeding; predominant breastfeeding rate (proportion of infants predominantly breastfed in the last 24 hours) | Formal and Informal work: professionals, students, skilled workers, petty traders, housewives | Occupation is associated with EBF (did not break down by type); Working outside the home cited as a reason for not practicing EBF. |
| 4 Ahinkorah, 2022, Chad^164^ | Cross-sectional | Mothers with children 0-2 y, n=3991 | EBF, Early initiation of BF | Not working and working | Higher EIBF among non-working mothers (AOR 1.37, 95% CI: 1.18, 1.59) |
| 5 Ahmed, 2019, Ethiopia^51^ | Repeated cross-sectional | Women living with a 0–23-month-old child, n=3680 (2000), n=3528 (2005), n=4037 (2011), and n=3861 (2016) | Early initiation of BF and EBF (according to the WHO/UNICEF); based on the maternal 24 h recall | Formal employment (professional, technical, managerial, clerical, and services areas); Informal employment (agricultural and manual work); No employment | Between 2000 and 2016, mothers who were informally employed were less likely to initiate breastfeeding within the first hour of birth compared to those who were un-employed. Informal employment had highest percent of EBF (60.2%). Informally employed mothers were less likely to initiate BF within the first hour of birth compared to unemployed. |
| 6 Alemu, 2020, Ethiopia^66^ | Cross-sectional | Mothers with infants 0-6 m, n=834 | EBF, colostrum given if baby had feed only breast milk within the first 6 m or  if first breast milk used by baby | Formal and informal work: Government, self-employed, housewife, other (student) | Good newborn care (EBF and colostrum) for employed mothers was 2.1 times higher (adjusted OR=2.1; 95% CI: 1.4-3.1). |
| 7 Anyanwu, 2014, Nigeria^114^ | Cross-sectional | Mothers who received perinatal care, n=143 | BF initiation, reasons for untimely BF initiation, intentions for EBF for 6 m | Occupation was not explicitly reported | 13.6% reported that they did not intend to EBF for 6 m due to their work/school schedule |
| 8 Appiah, 2021, Ghana^165^ | Cross-sectional | Mother 15-49 y with birth history and children born in 2 y preceding survey and practiced breastfeeding | Early initiation of breastfeeding | Not working and working; type of work not specified | Working women (AOR 0.90; 95% CI: 0.87,0.93) had lower odds of early initiation compared to not working women |
| 9 Argaw, 2019, Ethiopia^11^ | Cross-sectional | Mothers with infants <12 m and that lived in the area for 6+ m, n=634 | Prelacteal feeding practices (giving liquids/foods prior to establishment of regular BF); early initiation of BF (proportion of children born in the last 24 m made to BF within 1 h of birth); EBF | Housewives, farmers, civil servant, merchant, daily laborer, student, and house servant; employed mothers | Farmers practiced prelacteal feedings (AOR 4.33; 95% CI: 1.73-10.81, p-value = 0.002) up to four folds more than housewives. 13.3% of housewives had prelacteal feeds, 20.7% had not; for working/employed: 18.8% had prelacteal feeds, 42.5% had not; for farmers: 67.7% had prelacteal feeds, 36.7% had not |
| 10 Armar- Klemesu, 2000, Ghana^166^ | Cross-sectional | Households with children less than 3 y, n=556 | Child feeding index (giving the child prelacteal feeds and still BF; created for children 4 + m); First foods a child was given and child/mother interactions regarding food given | Formal and informal work: Current employment; not working, working part-time, working full time, unemployed. Also assessed place of employment (home, market/streets, and factory/office/shop). | No significant association between employment status and workplace with feeding index. However, the average feeding index score was less than -2, indicating a negative practice. |
| 11 Asare, 2018, Ghana^52^ | Cross-sectional | Mothers (15-49 y) who BF children aged 0-24 m, n=355 | EBF, continuous BF; Com. Feeding; bottle feeding; current BF practices (based off the last 24 h before the interview); EBF knowledge; early initiation of BF (proportion of children born in the last 24 m who were put to the breast within one hour of birth) and Colostrum feeding | Formal and informal work | Did not discuss that occupation was associated with EBF; Unadjusted OR for formal employees and EBF was 1; unadjusted OR for informal employees was 0.79 with a 95% CI: of 0.41, 1.56. |
| 12 Asekun- Olarinmoye, 2011, Nigeria^67^ | Cross-sectional | Mothers of children 0-2 y, n=500 | EBF, BF + artificial milk, artificial milk + weaning diet, maize gruel, and adult diet. | Formal and informal work: full-time housewives, unskilled workers, skilled workers, professionals. | Work and BF not linked in analysis. Compared mother's occupation in children attending daycare vs home. Discussion noted that work outside the home or return to full-time work is a likely factor responsible for ow EBF. Authors note that need for mothers to put their children in daycare centers arises from many of them work outside their homes. |
| 13 Asemahagn, 2016, Ethiopia^12^ | Cross-sectional | Mothers of infants 0-6 m, n=346 | EBF, accepting infants below 6 m who were fed only breast milk in past 24 h; early initiation of breastfeeding. | Housewife, merchant, government employee, non-government employee and daily laborer; employed vs unemployed; formal and informal work | Unemployed mothers were more likely to practice better EBF than employed mothers (OR 1.88; 95 % CI 1.05, 3.33). |
| 14 Asfaw, 2015, Ethiopia^13^ | Cross sectional | Mothers with infants less than 12 m, n=634 | EBF (according to WHO) (ideal practice); early initiation of BF; provision of colostrum; duration of EBF for the index infant; Prelacteal feedings: given to infant before they are put on the breast for the first time. | Housewife, working/employed or farmer | Odds of EBF for housewife was higher than employed mothers (OR 1.66; 95 % CI: 1.136, 2.41). No statistically significant difference comparing housewives and farmers: (OR 1.06; 95 % CI: 0.64, 1.75). |
| 15 Awoke, 2020, Ethiopia^68^ | Cross-sectional | Mothers with children at least 2 y old, n=347 | Optimal BF: initiation of BF within one hour of delivery, giving colostrum, exclusive BF for 6 m, introducing complementary food at 6 m and continued BF up to 2 y; knowledge about optimal BF and counseling at follow-ups. | Formal and informal work: Government employee, housewives, or other (merchant, private employee and daily laborer) | Government employees (AOR= 8.0; 95% CI: 1.7, 36.4) had a higher chance of practicing optimal BF; woman's occupation was significantly associated with optimal BF practice (Housewives: 39% had optimal BF practice; Government employees: 44.4% had optimal breastfeeding practice; Other: 47.6% had optimal BF practices) Government employed and other mothers were 8.0 and 8.3 times more likely to practice optimal BF than housewives. |
| 16 Ayawine 2015, Ghana^69^ | Cross-sectional | Nursing mothers of babies 0-12 m, n=300 | EBF; knowledge of EBF; EBF counseling and BF in public | Formal and informal work; mother's occupation: farming, trading, apprentice, or civil servant; availability and duration of maternity leave; number of days per week the mother works; whether infant comes with to work; food the infant is given and how it is stored | No significant difference in length of days spent at work (P=0.076). 79% and 96% of nursing mothers who have resumed work fed 0-6 m infants with a prepared cereal. 5.3% of mothers in Abuakwa fed babies with baby formula and expressed milk. No nursing mother in Barekese gave expressed milk to baby upon resumption of work. Maternal occupation was not significantly associated with EBF. |
| 17 Ayele 2019, Ethiopia^14^ | Case-control | HIV positive women (n=249; 83 cases and 166 controls) | Mixed feeding (giving child some breast feeds, and some artificial feeds); prelacteal feeding (administration of any food/drinks before first breastfeed); replacement feeding (no breast milk but adequate diet until child can be fed family food) | Employed or housewife; type of work not specified | Occupational status was found to be significantly associated with none EBF practice; employed women (AOR=4.363, 95% CI: 2.324 to 8.191) was a predictor of none EBF; Occupational status has a significant association in this study: employed women were found to be 4.363 times more likely to be practiced nonexclusive BF |
| 18 Balogun 2017, Nigeria^109^ | Cross sectional | Mothers of children under 5 n=248 | Knowledge and attitude of BF; initiating BF immediately after birth; currently BF; EBF; expressing breastmilk, frequency of BF, currently BF, age of weaning | Maternal occupation: senior professional, intermediate professional, junior professional, semi-skilled, unskilled, or homemaker/student | 79.8% of rural respondents practiced EBF compared to 29.0% of urban (P < 0.001); more urban women citing work resumption as reason for no EBF (P = 0.010); urban mother's occupation was sig associated with increased knowledge of breastfeeding (p<0.05) |
| 19 Bankole 2022, Guinea,^167^ Nigeria, Sierra Leone | Cross Sectional | Mothers with infants < 24 mo | Early initiation; EBF; partial BF | Employed vs unemployed (type of work not specified) | Unemployed mothers in Nigeria (0.03, 95% 1.03, 1.5) were less likely to practice optimal breastfeeding |
| 20 Basnet 2020, Ethiopia, Bangladesh, Vietnam^15^ | Cross Sectional | Mothers with child <5 y old; Ethiopia n=2746, Bangladesh n=4400, Vietnam n=4029 | EBF (proportion of 0-5.9 m old infants exclusively BF), Minimum Meal Frequency, dietary diversity for 6-23 m. | Employed vs. unemployed (as a measure of autonomy) (type of work not specified) | Not statistically significant, but OR in Bangladesh was 0.69, and in Ethiopia = 0.67, comparing the odds of EBF in employed vs unemployed mothers |
| 21 Bayissa, 2017, Ethiopia^70^ | Cross sectional | HIV+ mothers of children less than 6-12 m and using PMCTC services n=392 | Infant feeding practices (common infant feeding practices were BF, replacement feeding and mixed feeding); some practiced EBF and some practiced exclusive replacement feeding; maternal knowledge on MTCT of HIV | Maternal employment: housewife, government employee, merchant, others; formal and informal work | Self-employed women were 3.5 times more likely to practice EBF than government employees (OR, 3.51 95% CI:=1.65-6.37). Employment showed a significant association with EBF: OR (95% CI:) of EBF of self-employed mothers: 2.11 (1.03-4.29), AOR=3.51 (1.65-6.37). |
| 22 Begna, 2015, Ethiopia^98^ | Cross sectional | Mothers who breastfed for between 6 m and 2 y and were a resident of selected kebeles n=403 | knowledge and practice of BF; knowledge of optimal child feeding practices and socio-cultural influences of child feeding | Occupation of Mother (Employee (GO/NGO) vs. Merchant vs. Housewife vs. Student vs. Farmer vs. Daily worker vs. other) | Housewives had 2.42 times the odds of EBF compared to employees (GO/NGO). (95%CI: 1.36, 4.33, p=0.022). Other groups had lower odds of EBF compared to the employee (GO/NGO). |
| 23 Bekele, 1999, Ethiopia^168^ | Cross-sectional | Mothers of children 0-23 m living in study villages n=1536 | Methods used in child feeding: Bottle alone, breast and bottle feeding, total bottle fed, EBF, partial breastfeeding, total never bottle fed | No work outside home vs. work outside home (type of work not specified) | The odds of bottle feeding were 3.02 times higher for mothers who work outside the home compared to those who did not work outside the home. |
| 24 Berde, 2018, Namibia^16^ | Cross sectional survey | Mothers with infants less than 24 m from Namibia DHS n=1926 | Drinking anything from a bottle the night before | Mothers occupation: Not working or working | Working mothers had 51% higher odds of Bottle Feeding after 6 m compared to non working mothers. Bottle feeding as compared to cup feeding (preferred) |
| 25 Berde, 2016, Nigeria^71^ | Cross-sectional; secondary analysis of DHS data | Mothers with last-born children born in the past two y n=11910 | Initiation of BF within 1 h of birth (early) and initiation of BF after 1 h (late) | Mothers occupation: Not working or working | Working mothers had 51% higher odds of BF compared to non-working mothers. |
| 26 Berhe, 2017, Ethiopia^72^ | cross sectional | Mothers with children less than 6 m n=423 | Optimal BF: initiation of breastfeeding within the first one hour of delivery and no additional feeding given. | Categories: housewife, student, employee, merchant | Occupation was associated with essential newborn care (BF was not separate) and significantly associated with essential newborn care. |
| 27 Bodjrènou, 2021, Benin^73^ | Cross-sectional | Mother-child pairs 0-17mo in 8 villages | Breastfeeding on demand, breastfeeding frequency during children illness, and positioning and attachment of children while breastfeeding, complementary feeding (Minimum dietary diversity, minimum meal frequency, minimum acceptable diet) | Formal and informal work; categories: No activity, food processing, agriculture, animal breeding, trading, foods selling, handicraft, number of activities. In analysis compared working in Agriculture vs. not, working in trading vs. not, working in animal breeding vs. not, working in food-processing vs. not. | The odds of breastfeeding on demand for those working in agriculture were 1.7 times that of those not working in agriculture (working in any other setting) however the CI and p-value are not significant.  The odds of breastfeeding on demand for those working in trading were 1.9 times that of those not working in trading (working in any other setting). (CI: 1.07-3.35, p=0.02817)  The odds of having good positioning and attachment for breastfeeding for those working in animal breeding were 0.47 times that of those not working in animal breeding (working in any other setting) however the CI and p-value are not significant.  The odds of having good positioning and attachment for breastfeeding for those working in trading were 0.61 times those not working in trading (working in any other setting) however the CI and p-value are not significant. |
| 28 Cherop, 2009, Kenya^169^ | Cross-sectional | Mother-infant pair (infant 0-6 m) n=384 | EBF up to 6 m and mixed feeding | Mentioned among the barriers to EBF; type of work not specified | Mothers (6.1%) who worked outside home did not have time to BF and introduced other foods. No EBF for mothers working outside home. This reason scored highly among mothers with infants aged 3-4 m (8/90) vs 5.7% (4/72) among infants 0-2 m. |
| 29 Chineke, 2017, Nigeria^74^ | Cross-sectional analytical | Nursing mothers within Imo State University Teaching Hospital n=200 | EBF for 6 m | Type of occupation: Civil servants, Housewife, Trader, Student, Others | Being traders [OR: 0.24; 95% CI: (0.109-0.551); p<0.000] and students [OR: 0.27; 95% CI: (0.090-0.835); p=0.019] were significantly less likely to practice EBF compared to housewife. |
| 30 Chuwa, 2013, Tanzania^75^ | Cross-sectional | Mothers of infants 0-6 m n=92 | EBF for 6 m | Type of occupation: Business, Employed, Peasants, Housewives  formal and informal work | Short maternity leave found to accelerate ineffective BF; Unsupportive working environment |
| 31 Dalcastagne, 2018, Angola^53^ | Cross-sectional | Children 0-23 m n=749 | Prevalence of: Early initiation (within the first hour), EBF, Complementary feeding as infant approached 6 m | Survey: formal employment (those working mostly in the public or private sector of work), informal employment (self-employed); formal and Informal | Formally employed mothers' prevalence of EBF under 6 m was observed to be 54% higher than self-employed mothers. OR= 1.54 (1.05, 2.26). |
| 32 Dare, 2011, Nigeria^76^ | Cross-sectional | Married/single women practicing artificial or BF  and who nursed children before, n=255 | EBF, complementary feeding | Self-employment (farmers, artisans, petty traders, civil servants (teachers, nurses, office workers). Described mothers' hours of work per day: 7-8 hours per day, 9-12 hours per day. Formal and Informal | Higher EBF among mothers who work for 7-8 hours (44.8%) than 9-12 hours. Significant difference in the pattern of BF between mothers who work for less than 8 hours and who work more. Civil servant mothers work about 8 hours per day and most live in the same town where they work, usually have a break period during working hours |
| 33 Davies-Adetugbo, 1996, Nigeria^77^ | Cross-Sectional | Lactating mothers with a BF history and one living child in good health, n=102 | Time of initiation of BF, time of initiation of human milk substitutes, BF duration | Occupation (housewife as the reference, trader, artisan, clerical work), and work away from home. | The artisan occupational group (OR = 0.31, p = 0.0 147) decreased the initiation of BF. Average duration of BF (in m) of mothers who worked away from the home was 14.1 (4.2) and was 17.6 (3.2) for full-time housewives. The difference of 3.5 m is significant (Kruskal-Wallis H = 20.29, p = 0.0000). At 2 wk the artisan introduced human milk substitutes than housewives (10.39 (1.36 to 79.39) 0.0241 |
| 34 De Onis, 2006, USA, Norway, Brazil, Ghana and India ^170^ | Longitudinal | 0-24m, n=903 | Early initiation of BF; EBF: receiving only breast milk from mother, and no other liquids or solids; Predominant BF: breast milk as predominant but infant could also receive water/water-based drinks fluids; Complementary between 4 and 6 m, feeding and partial BF | Maternal employment (employed outside of home, full time job) and type of job (not described in detail). | Fewer mothers employed outside home in compliant group compared to non-compliant group. Mothers who complied with MGRS feeding were less likely to be employed outside home. |
| 35 Deselew, 2020, Nigeria^3^ | Cross-sectional | Mothers with infants 6-23m, n=704 | EBF | The mother's occupation mentioned as Yes or no and in table 4 the maternal employment Yes or no; no specific categorization of occupation | The odds of EBF practice were 1.93 times higher among the unemployed mothers, compared to those reported for the employed subjects (AOR: 1.93;95% CI: 1.17-3.20). |
| 36 Diji, 2016, Ghana^17^ | Cross-sectional study | Mothers with healthy infants aged 3-9m, n=240 | EBF ("defined as the practice whereby infants receive breast milk alone with no additional fluids or solids other than syrups containing vitamins, mineral supplements, or medicines"; "in this study, EBF refers to birth up to the time of the interview for mothers with infants up to six m of age"); EBF challenges | Formal and informal work; categorized as unemployed, self-employed, privately employed, or publicly employed | Short maternity leave was found to be a breastfeeding challenge of mothers (mean 3.41 (SD 1.29); self-employment was an independent predictor of EBF (AOR 2.67 (95% CI: 1.11, 6.41)); "while 30% of the mothers who used mixed/formula feeding were unemployed, only 11.2% of those who practised EBF fell into this category"; unemployed: 42.9% EBF, 57.1% non-EBF; self-employed: 72.4% EBF, 27.6% non-EBF; privately employed: 69.6% EBF, 30.4% non-EBF; publicly employed: 72.2% EBF, 27.8% non-EBF; "Compared to their counterparts who practiced mixed/formula feeding, those who practiced EBF experienced significantly higher level challenges regarding...difficulty combining work and breastfeeding (U=4250, p=0.000); "Relative to mothers who were unemployed, those who were self-employed were 2.60 times more likely to practice EBF" |
| 37 Doherty, 2012, South Africa^171^ | Community-based cluster-randomized trial | Pregnant women in third trimester of pregnancy; mother-child pairs assessed at 3, 6, 12, and 24 wk after birth, n=999 | Complete cessation of BF (no BF in 24hours and 7 days prior and no BF reported in following interviews) by 3, 6, or 12 wk. | If mother earns money for herself | Mother earning money for herself was associated with higher odds of stopping BF (OR 1.9, 95%CI 1.3-2.8) by 12 wk |
| 38 Ekanem, 1993, Nigeria^61^ | Cross-Sectional | Women with children 0-24 m, n=240 | BF practice and duration | Unclear, women largely from low socioeconomic group and mainly engaged in non-professional occupations (petty trading/food hawking) | Reason for stopping BF was that the mother had to resume work- looks like around 10% |
| 39 Esan, 2013, Nigeria^172^ | Cross-sectional | Female physicians in public facilities in the selected local government authorities, n=50 | EBF for 6 m, time to initiate BF (1 h, 1-<=24 h) and >24 h), and continued BF to 2 + y. | Among the reasons for stopping EBF; Formal work only | Return to work was among the main reasons for stopping EBF, reported by 8 of 67 (11.9%) of female physicians. |
| 40 Eskezyiaw 2014, Ethiopia^78^ | Cross sectional | Mothers with children 6 m-2 y, n=562 | Early initiation, CF (before 6 m; foods/liquids provided along with BF; timely initiation CF time of of supplementary food at 6m. | Formal and informal work: Daily laborer, private business (merchant, farmers), government, housewife. | Reason for early start to CF was working outside of home; crude and adjusted OR and p-values and percentage of CF for different occupations in Table 2. |
| 41 Feleke 2021, Ethiopia^173^ | Cross-sectional | Mothers of infants 6-12m, n=860 | Non EBF is giving infants other foods or fluids in addition to the breast milk other than drugs, vitamins, and minerals to the infants before the age of six m;  EIBF is infants breastfed within one hour of birth | Government employee, farmer, merchant, day laborer, housewife | Government employee (AOR=8.20 95% CI: 5.191, 12.940) Daily laborer (AOR 1.7 95% CI:1.01,2.90) and merchant (AOR 0.44, 95% CI:0.35, 0.90) were significantly associated with not practicing EBF. |
| 42 Gara 2005, Zimbabwe^18^ | Prospective Survey | Women with children 0-2 y n=200 | Mixed Feeding, EBF, Formula Feeding | Paid employees; employed vs. unemployed | Paid employees largely resorted to mixed feeding. (42%, 3.4% practiced EBF)  EBF was more popular in the unemployed (78%) (p<0.05) |
| 43 Gebremedhin 2021^79^ | Cross-sectional | Mother with children aged 0–23 months, =1406 | Exclusive breastfeeding practice 24 hour recall for those less than 6 months six months EBF for those aged 6 to 23 months | Respondent Occupation; No work Technical; Agricultural Others | Employment status was significantly associated with low EBF |
| 44 Gebriel,  2000, Ethiopia^97^ | Cross-sectional | Mother-child pairs of 0-2 y children in semi-urban district of Adigrat, Tigari, Ethiopia, n=334 | Weaning food: any food items including water given after the age of four m  early weaning: when supplementation of foods in addition to breastfeeding started before the age of four m  correct weaning: when additional food is supplemented between the age of child and recommended duration  delayed weaning: additional food is supplemented after the age of four and 6 m  bottle-feeding: ever use of bottles for the index child-feeding | Working at home vs. work on a day basis vs. self-employed vs. employed by government | OR: 3.45 (95% CI: 1.61, 8.14) for early weaning in those who work outside of the home compared to reference group (housewives) (regardless of educational and income status?)  A larger proportion of mothers who worked outside the home bottle-fed compared to mothers who worked at home, mothers working outside the home had three times higher odds of bottle-feeding compared to housewives. |
| 45 Ghuman 2009, South Africa^115^ | Prospective cohort | Mothers who delivered over a one-month period (interviewed at birth and 14 wk later) n=168 (first interview) 117 (second interview) | BF initiation; infant feeding intentions; infant feeding practices at 14 wk (mixed feeding, EBF, formula feeding) | Maternal occupation measured | By 14 wk, 11% of HIV positive mothers practiced EBF, while 12/19 [63%] practiced mixed feeding due to their need to return to school (40%) or to work (20%); Convenience was the most common reason for mixed feeding; reasons for changing feeding practice was work related (over 20%) |
| 46 Goon, 2021, South Africa^160^ | Cross-sectional study | Mothers of infants aged 18-29 m on ART  n=469 | Exclusive breastfeeding (nothing but breast milk) measured by asking mothers if they initiated EBF for the baby and to state the duration of EBF in m. (mothers who breastfed infants exclusively for 1 to 3 wk were coded as 1 month). | Employment was measured by asking if respondents had worked for wages or salary in the past 12 m or not; type of employment not specified | Unadjusted: being unemployed (COR: 1.77, 95%CI: 1.17, 2.68) was associated with a higher odds of practicing EBF for the first 6 m. Adjusted: Being unemployed (AOR: 1.66, 95%CI: 1.08, 2.56, p<0.05) was significantly associated with a higher likelihood of exclusive breastfeeding. |
| 47 Hailu, 2020, Ethiopia^80^ | Cross-sectional | Mothers with an infant 6-12mo, n=605 | Breastfeeding performance index (BPI) scores assessed positive BPI scores of 1 point each for seven healthy breastfeeding practices: early breastfeeding initiation; pre-lacteal feeds; bottle feeding; exclusive breastfeeding; not receiving liquids; not receiving formula or other milk, and not receiving solids). S cores were categorized as “poor” breastfeeding practices (0-5) or “good” breastfeeding practices (6-7). | Occupational status | BPI scores were significantly associated with maternal occupation |
| 48 Horii, 2017, Niger^19^ | Cross-sectional | Mothers (15-49 y) with a child below 24 m, n=1026 | Early initiation | Self-report; formal and informal work | Sales or service workers were 7.7 times more likely to practice early initiation compared to household workers with no income (95% CI: 1.3, 47.8). |
| 49 Horwood, 2020, South Africa^116^ | Cross-sectional | Caretakers with infant 13-16 wk, n=4172 | Mothers asked various questions about feeding practices help determine feeding measures; introduction of other foods/liquids, baby formula, prelacteal feeds, EBF (consistently reported only giving breastmilk) | Two surveys were used to assess employment and determine changes in duration. Ex: mother returning to work. Type of work not specified | Employed mothers who had returned to work or school were less likely to practice EBF at 14 wk (AOR 3.8; 95% Cl 3.1-4.6) |
| 50 Horwood, 2018, South Africa^174^ | Cohort | Caregivers with infants of approximately 14 wk, n=4172 | EBF, introduction of food or fluids to infant, formula milk, pre lacteal feeds, mixed feeding | Self-report. Employment and unemployment: no paid work past 12 m, paid work but not since baby born, and paid work since baby born. | Work heavily influenced the mother's decision to BF or EBF. bi-variable analysis: mother return to work 0.2 (0.4-0.7). multivariable analysis: mother return to work 0.3 (0.2-0.4) |
| 51 Hussein, 2019, Tanzania^20^ | Longitudinal cohort | Women (15-48 y) in third trimester of pregnancy followed through 9 m, n=430 | EBF using 24 hours recall: mother asked to list foods that were given to the child apart from breast milk for the past 24 hours. Second recall since used to assess EBF practice at monthly visits. | Employed or unemployed; formal and informal work | Unemployed women had increased odds of practicing EBF [OR=1.5; 95 % CI: 1.1, 2.5]. Unemployed was protective of EBF practice using recall since birth method [OR=0.9, 95% CI: 0.5, 1.6] but not statistically significant. |
| 52 Igbedioh, 1992, Nigeria^82^ | Cross-sectional | Mothers who regularly visited the post-natal health clinics n=100 | BF and weaning practices; effect of severity of cost of living on BF pattern; intended age of cessation and factors influencing the decision; starting age of supplementary feeding and type fed; factors influencing milk formula fed and type; age of introducing milk formula; type of legumes fed | Employment: civil servant, trader, farmer/fisherman, food seller, not employed, teacher, general laborer, and craft maker | Relationship between occupation and time/duration of introducing milk formula; the high number of respondents who BF on demand (92%) correspond with the high number of self-employed or unemployed respondents (80%). Most respondents who planned to cease BF when their children would be above 1 1/2-year-old, were self- or unemployed. Better educated women in paid employment breastfed for shorter time. |
| 53 Igbedioh, 1995, Nigeria^81^ | Cross-sectional | Mothers (infants 4-9 m old) who regularly visited the post-natal health clinics, n=200 | Food children were fed; BF initiation, practices, and feeding frequencies; intended age of cessation of BF; age of introducing milk formula, supplementary feeding, feeding pap, and the influencing factors; Weaning food used; mode of feeding gruel and storage conditions; feeding of legumes | Occupation: civil servants, trader, farmer/fisherman, food seller, business, crafts making, and unemployed | Maternal employment cited as a main reason for the use of milk formula (3%); Self-employed or unemployed planned to finish BF earlier than employed mothers. |
| 54 Iliyasu, 2019, Nigeria^146^ | Cross-Sectional | Female health care workers, with children 0-5 y.   n=261   Urban | Prevalence and practice of EBF, support of BF in the workplace, knowledge, and attitude towards EBF | Formal and informal work | Self-employed mothers were more likely to practice EBF than their unemployed counterparts (AOR = 2.61; 95%CI: 1.09, 6.22).  The second most frequently reported challenge to EBF was a short maternity leave period (mean m = 3.41. |
| 55 Issaka, 2014, Ghana, Liberia, Nigeria, and Sierra Leone^175^ | Cross-sectional | Infants 3-5 m from four Anglophone West African countries n=2447 (n=166 in Ghana, n=263 in Liberia, n=1658 in Nigeria, n=360 in Sierra Leone) | Early introduction of solid, semi-solid or soft foods | Mother's work status: working (past 12 m) or non-working | EISF rates were not significantly different between working and nonworking in all the countries except in Nigeria (employed mothers reported a significantly higher rate); no association found between the working status of mothers and EISF |
| 56 Jahanpour, 2022, Tanzania^176^ | Cross-sectional | Mothers with infants 0-6mo,  n=1216 | EBF | Informal work only; working for family members/someone else | Mothers who were self-employed (AOR  =1.9; CI 95% 1.2‚ 3.0; p = 0.008) or not working (AOR = 1.98; CI 95% 1.3‚ 2.9; p = 0.001) were more likely to practice EBF compared to those employed by a family member/someone else; Need for social support to decrease the work-load for breastfeeding mothers so as to permit her to EBF |
| 57 Jimoh, 2018, Nigeria^177^ | Cross-sectional | Mothers 14-49 y and those less than 14 y in a marital relationship, n=270 | Prelacteal feeding practices | Work (income generating activity) or no work | Among mothers who worked: 171 (84.2%) were prelacteal feeding, 32 (15.8%) were not; among mothers who were NOT working: 59 (88.1%) were prelacteal feeding, 8 (11.9%) were not; P-value: 0.445 |
| 58 Kaldenbach, 2022, South Africa^178^ | Cross-sectional | Mothers with infants aged 25-31 wk, n=774 | BF; early introduction of other foods/liquids; 24-h food and fluids recall; breastfeeding knowledge and attitudes | Returning to work or school | At time of interview, 22.5% of mothers had stopped BF, with 33.1% citing returning to work as a reason for stopping. At time of interview, 52.7% were BF but had introduced other fluids or foods, with 26.1% citing return to work or school as a reason. Maternal return to work/school associated with stunting and selected risk factors [AOR = 0.91; 95% CI: 0.42, 1.91] |
| 59 Kebebe 2017, Ethiopia^83^ | Community-based cross-sectional | Mothers/caregivers of children (>23 m), n=418 | Bottle feeding intention (plans of bottle feeding in future) and practice (proportion of children that were fed any liquid (including breast milk) or semisolid from bottle in past 24 hours) | Occupation: housewife, merchant/private job, employed, others | Reason for bottle feeding: Work (outside home) (48/82, 58.5%) and reason for intended bottle feeding included return to work, (63/93, 67.7%). Housewives were 62.5% times less likely to practice bottle feeding than mothers with outside home job (AOR = 0.375; 95% CI: 0.208, 0.676). |
| 60 Kenechi, 2015, Nigeria^84^ | Cross-sectional | Mother infant pairs (infants <6 m) attending the infant welfare clinic, n=400 | Infant feeding practices: EBF practices, predominant breastfeeding (PBF), complementary breastfeeding (CBF), and breastfeeding (BF); infant's first feed, everything baby took in the previous 24 hours, reason for stopping BF; knowledge of EBF and willingness to practice EBF | Categorized as subsistence farmer, business, or housewife | Those in subsistence farming (OR 0.67, CI -.38-1.45) were less likely to practice good newborn care and more likely to initiate BF early; occupation was a main predictor of essential newborn care.  Early BF: 90.0% housewife, 5.3% subsistence farmer, 4.7% business; P-value for occupation and early breastfeeding = 0.010; Early BF: housewife (univariate OR: 1, 95% CI: 1, multivariate 95% CI: 1), subsistence farmer (univariate OR: 2.93, 95% CI: 1.35,6.35; multivariate OR: 2.54, 95% CI: 1.12,5.77), business (univariate OR: 0.44, 95% CI: 0.06, 3.35; multivariate OR: 0.45, 95% CI: 0.55,3.63). |
| 61 Komakech, 2020, Uganda^85^ | Cross-sectional | Mothers with infants 0-6 m in refugee settlements n=561 | EBF practices, age of introduction of complementary foods/fluids; type of food/fluids introduced; nutritional status measures | Sources of livelihood; small stock keeping, arable farming, salary-paid jobs ,cattle production, Poultry , Traditional beer processing, Vending, veld foods , others | Reasons for mothers to stop BF were either that the mother was at work or school. |
| 62 Kulwa, 2006, Tanzania^179^ | Cross-sectional | Mothers of children 6-24m, n=100 | EBF practices, age of introduction of complementary foods/fluids and type of food/fluids introduced; nutritional status measures | Formal and informal | Resumed work was the mother's reason for introducing complementary foods among 27% of children. |
| 63 Lakati, 2002, Kenya^153^ | Cross-sectional | Mothers with children 4-12 m, n=444 | BF duration, frequency, and complementary feeding | Formal and informal | During the day, 29.7% of the mothers from higher socioeconomic group (71% of which are formally employed) fed their babies. 79.4% from lower socioeconomic class (19% are formally employed). At 1 and 2 m after birth, mode of work (fixed hours vs. shift work) was associated with EBF (OR=0.451, 95%CI: 0.243, 0.836) and (OR=0.39, 95%CI: 0.206, 0.738), respectively. |
| 64 Lakew, 2015, Ethiopia^180^ | Cross-sectional | Women 15-49 who were usual residents, n=11,654 | Timely initiation of BF (within one hour): by asking mothers to provide information regarding the time at which infant was put to the breast after delivery | Occupational status: non-working (housewife) and working (professional/  technical/managerial, clerical, sales and services, skilled manual, unskilled manual and agriculture classifications). | Working mothers were 23% less likely to timely initiate BF (AOR 0.77, 95% CI: 0.69,0.85); prevalence of early initiation of BF by occupation: housewife: 57.6 (95% CI: 56.3, 58.9) and working: 53.5 (95% CI: 48.7, 51.2). |
| 65 Lawani, 2014, Nigeria^21^ | Cross-sectional | Parturients infected with HIV/AIDS who attended the PMTCT/pediatric clinics when their child was 18 m old n=556 | Challenges in adhering to their infant feeding preference/ recommendations for PMTCT of HIV; infant feeding practice: EBF, exclusive formula feeding, mixed feeding; who made decisions regarding feeding and reasons/time of mixed feeding; duration of BF | Employed or unemployed; job commitment was considered a potential reason for mixed feeding | Adherence was strongly associated with age, marital status, and employment status, but not with residence, educational status, or parity; Adherence was higher in the unemployed (OR, 2.10; 95% CI:, 1.20-2.45) and lower in the employed (OR, 0.78; 95% CI:, 0.68-0.90)"; job commitment was cited by 1 (2.4%) woman as a reason for mixed feeding. |
| 66 Lawoyin, 2001, Nigeria^110^ | Cross-sectional | Mother-infant pairs (singleton deliveries, infants 6 m old) n=2794 | EBF | Occupation classified as higher (professionals and the top civil servants- managerial cadre of workers), middle (technical and skilled workers), and lower (partially skilled and the unskilled). Formal/informal | Factors independently associated with EBF was higher maternal occupation (OR 1.6; 95% CI:; 1.02 - 2.5). |
| 67 Liben, 2016, Ethiopia^86^ | Cross-sectional | Mothers of infants less than 6 m n=333 | EBF, Prelacteal feeding & Colostrum feeding , early initiation | Housewife, Government employed, others (farmers, daily laborer, traders); formal/informal | Housewife (unemployed) mothers were more likely to practice EBF (AOR 4.81;95 % CI 2.30, 10.06). |
| 68 Mahgoub, 2002, Botswana^87^ | Cross-sectional | Children under 3 y n=400 | early initiation, BF | Sources of livelihood; small stock keeping, arable farming, salary-paid jobs, cattle production, Poultry, Traditional beer processing, Vending, veld foods, others | Reason for mothers to stop BF were that the mother was at work or school |
| 69 Manyeh, 2020, Ghana^88^ | Cross-Sectional | Mothers with children at least 6 m old, n=1870 | EBF | Sociodemographic determinants: occupation type (Farmer, artisan, unemployed, etc.).  Self-report, Formal and informal | Farmers were 77% more likely to practice EBF than unemployed mothers (COR 1.77, 95%CI (1.23, 2.55). Artisans were 36% less likely to practice EBF than unemployed mothers (AOR 0.64, 95%CI (0.43, 0.96). |
| 70 Matanda, 2014, Kenya^111^ | Cross-sectional | Dyads of mothers aged >49 and children aged >23 m, n=2125 | Early initiation of BF within an hour of birth | Early BF initiation by occupation categories: white-collar, blue-collar, or not working; formal/informal | Blue-collar and not working mothers were more likely to initiate BF within one hour across surveys, but associations were not statistically significant. KDHS 1998: Blue-collar (OR=1.13, 95%CI 0.80 to 1.61); not working (OR=1.17 95%CI 0.86 to 1.60). KDHS 2003: Blue-collar (OR=1.09, 95% CI: 0.79 to 1.50); not working (OR=1.13 95% CI: 0.81 to 1.57). KDHS 2008-9: Blue-collar (OR=1.47, 95% CI: 0.98 to 2.20); not working (OR=1.57 95%CI 1.03 to 2.42). Non-working women were significantly more likely to initiate BF within one hour than the white-collar women. |
| 71 Mbawalla 2017, Tanzania^117^ | Cross-sectional | Mothers with children 12 m or below, n=213 | EBF, complementary feeding | Self-report (survey)  formal/informal | N/A - the study focused on the connection between oral health and employment, but not employment and EBF. |
| 72 Mekuria 2015, Ethiopia^118^ | Cross-Sectional | Mothers with infants less than 6 m, n=413 | EBF, Complementary feeding | Quantitative survey compared EBF practice by sociodemographic categories, including employment; Self-Report; type of work not specified | Work was cited as one of the most common reasons for no EBF. Unemployed mothers were 1.98 times more likely to practice EBF than employed mothers (AOR=1.98 (1.21, 3.22). |
| 73 Mensah, 2017, Ghana^22^ | Cross-Sectional | Mothers with children 1-6 m, n=380 | EBF | Quantitative survey compared EBF practice by sociodemographic categories, including employment type (self, public, private, unemployed); Self-report, formal and informal work | Women who reported private (formal) employment were more likely to practice EBF up to 5-7 m (15.0%) compared to publicly employed mothers (1.58%) (X^2=18.5756, p=0.005). Self-employed mothers are more likely to practice EBF than privately or publicly employed mothers (in Discussions, but not in Tables). |
| 74 Mohammed, 2021, Nigeria^154^ | Cross-sectional | Mothers who were currently BF at time of study or who had stopped BF not greater than 2 y, n=270 | EBF at 6m | Formal and informal work; Occupation | 37 (82.22%) of the health workers practiced EBF, 35(79.55%) of teachers, 65(71.43%) of those in business, and 48(53.33%) of those doing other jobs practiced EBF, respectively (X^2=16.736, P=0.001). |
| 75 Morhason-Bello, 2022, Nigeria^181^ | Cross-sectional | Nursing mothers, n=58,244 | Early initiation of BF | Formal and informal work; Occupation (not working, unskilled/ clerical/ household and domestic, agricultural, skilled/ sales/ services, professional/ technical/ managerial | In the 2008 survey, women who are skilled workers were 1.08 (95% CI: 1.01- 1.16) times more likely to initiate breastfeeding early compared to those who are not employed.  In 2018, women who were skilled workers were 0.89 (95% CI: 0.83 -0.94) and 0.91 (95% CI: 0.85 - 0.97) less likely to initiate breastfeeding early compared to those who were not working. |
| 76 Motee 2013, Mauritius^112^ | Cross-sectional | Mothers 18-45 y with children under 5 y n=500 (n=216 urban; n=284 rural) | EBF; Duration of BF; Cessation of BF | Resumption of work, Student, blue collar, white collar housewife, self-employed | Barriers to EBF include employment (27.3%). Even if women are employed as professionals (28.3%) or are housewives (26.3%), they are more likely to discontinue BF within 24 m. Associations between the duration of BF and parity, alcohol consumption, education, and occupation. |
| 77 Muluye, 2012, Ethiopia^89^ | Cross-sectional | HIV positive mothers with child less than 2 y old n=209 | EBF, mixed BF, exclusive replacement feeding; recommended infant feeding practice: those who practiced EBF or exclusive replacement feeding; Not Recommended: mixed BF | Occupational status: housewife, daily laborer, government worker, private/merchant/farmers/others; formal and informal work | Disclosure of HIV status with their spouse, insufficient breast milk and occupational status (Housewife, day laborer vs other categories) were independently associated (p-value < 0.05) with recommended infant feeding practice (EBF and ERF) AOR = 7.7 (95% CI: 1.1, 53.97), AOR = 0.14 (95% CI: 0.03, 0.65), AOR= 14.6 (95% CI: 1.36, 156.40). Daily laborers were 14.6 times more likely to have recommended way of infant feeding practice than private/merchants. |
| 78 Napyo 2020 Uganda^23^ | Prospective cohort | HIV positive mothers and infants, n=466 | Prelacteal feeding 7-day recall EBF at 6 weeks and 14 weeks of age | Employed vs unemployed | No association between employment status and EBF |
| 79 Ndiokwelu, 2014, Nigeria | Cross-sectional | Mothers with children 0-24 m, n=200 | BF, EBF, and complementary feeding practices | Civil Servant, business, unemployment/Student, Medical Health Personnel, Others; formal and informal work | Positive relationships between mother's occupation and cessation of BF |
| 80 Nieuwoudt, 2018, South Africa^24^ | Cross-sectional | HIV positive and negative mothers with infants < 6 m from health clinics where primary health care is provided, n=298 | Ever BF, time to initiation of BF, EBF, predominant feeding, Exclusive formula feeding and mixed feeding using 24 hours period; feeding practices assessed whether lifetime or current feeding at 3 m and 3-6 m | Employed vs unemployed | Formula-feeding mothers were significantly more likely to be employed (35.6%) than breastfeeding counterparts (16.3%) Unemployed mothers had nearly double the odds (1.88, 95% CI: 1.03 - 3.44) of EBF compared to employed |
| 81 Nyanga, 2012, Kenya^25^ | Cross-sectional | Lactating mothers with infants aged 0-6 m, n=117 | EBF | Employed; not employed | Employment strongly influenced the EBF whereas non-employed mothers breastfed more by 42 percentage points compared to employed (P=0.00). Chi-squared usage to compare the relationship |
| 82 Ogbo, 2015, Nigeria^182^ | Longitudinal study (DHS data) | Mothers with children under 24 m n=88152 total (n=8199 in 1999, n=7620 in 2003, n=33385 in 2008, n=38948 in 2013) | Early initiation of BF (put to the breast within 1 hr of birth-based on mother's recall; EBF, predominant BF and bottle feeding rate (any liquid or semi-solid food from a bottle with nipple/teat) | Employment status: not working or working in past 12 m | P-value = <0.001 for interaction of mother's employment (working or not working) with early initiation of BF. P-value = 0.176 for interaction of mother's employment with EBF. P-value = 0.007 for interaction of mother's employment with predominant BF. P-value = 0.126 for interaction of mother's employment with bottle-feeding. Proportion of early initiation of BF decreased over the 4 time points, but slight increase in the intervening year among women with no schooling, unemployed mothers, poorer households (those with no health service contacts); employment was not associated with EBF |
| 83 Ogunlesi, 2010, Nigeria^55^ | Cross-sectional | Mothers with children 1-24 m attending a Nigerian Infant Welfare Clinic, n=262 | Poor BF practices include  delayed initiation of BF, use of pre-lacteal feeds, and failure to practice EBF for the first 6 m; observed delayed initiation of BF and EBF | Occupational groups: professionals (bankers, nurses, teachers, administrators and physicians) and others (traders, artisans, technicians, dress markers, farmers and students) | Professionals and the others who were commenced on BF within 1 h of birth were similar [26 (45.6%) vs. 72 (35.1%); X^2=2.097, P=0.1]. Lower proportion of professionals had prelacteal feeding [12 (21.1%) vs. 84 (41.0%); X^2=7.625, P=0.006], the EBF rate was similar in both groups 10/46 (21.7%) vs. 28/150 (18.7%); X^2=0.213, P=0.645]. EBF failure was significantly associated with low education, occupation as a professional, and delivery outside health facilities.  Occupation as a professional showed no advantage for BF practices, but professionals avoided prelacteal feeding, delayed initiation of BF, and failed to practice EBF. |
| 84 Ogwu, 2016, Botswana^26^ | Randomized clinical trial | HIV-positive pregnant women up to 6 m post-partum (counselled to practice EBF for 5 m), n=677 | Duration of total BF and reasons for discontinuation earlier than recommended | unemployed, employed/student, or self-employed | Urban location  (aHR=1.86, 95%CI: 1.27,2.73; P=0.002), salaried employment or being a student (aHR=2.78, 95% CI: 1.63, 4.75) and infant hospitalization before weaning (aHR=2.04, 95% CI: 1.21, 3.45) were associated with early BF cessation; employed/students (54%) were mostly likely to wean before 5 m (HR = 4.43; 95% CI: 3.27, 6.00). |
| 85 Ohaeri, 2016, Nigeria^91^ | Cross-sectional | Nursing mothers with children 0-2 y attending, n=210 | Survey with questions on practice of EBF | Survey (Trader vs. Civil Servant vs. Student vs. Artisan) | 90% answered that work discourages their choice of EBF;  Significant relationship between occupation and practicing EBF (chi-square=25.66, DF=3, p<0.0005)  16.67% of traders, 32.38% of civil servants, 14.76% of students, 5.71% of artisans were currently practicing EBF. |
| 86 Okechukwu, 2020, Nigeria^183^ | Cross-sectional | Female bankers who had children between 0-5 y, n=41 | EBF | Informal work only; bankers | Respondents agreed that childcare -related challenges created a challenge for practicing EBF among employed mothers. |
| 87 Okeh, 2010, Nigeria^92^ | Cross-sectional | Mothers attending postnatal unit, n=190 | EBF (unclear how this was determined) time intervals at which mothers BF their children | Civil servants, traders, students and housewife; formal/informal | A significant relationship between the category/occupation of mothers and time intervals at which mothers BF; EBF was dependent on occupation. |
| 88 Olaitan, 2015, Nigeria^119^ | Cross-sectional | EBF practicing mothers (250) vs non-EBF mothers (250), n=500 | EBF | Employment was not reported; maternal work was a reason for non-EBF | No EBF due to work demands and inability to preserve expressed milk (32% of mothers) |
| 89 Pascale 2007, Cameroon^27^ | Cross-sectional | Infants (0-1 yr) and mother; n=171 | BF and Mixed Feeding | Mothers Profession (jobless housewives vs. small job vs. workers) | Profession was significantly correlated with the length of BF, with housewives having the longest time of BF, compared to those with “small jobs” and full-time “workers”. |
| 90 Qureshi, 2011, Nigeria^28^ | Quasi-experimental; pre/post intervention surveys | Biological mothers who BF, n=358 | EBF, predominant BF, partial BF, any BF, prelacteal feeds | housewife vs civil servant/trader | lower EBF practice among working mothers |
| 91 Reda, 2019,  Ethiopia^93^ | Retrospective cohort study | Mothers with children 6-24 m, n=639 | Time to initiate complementary feeding (solid, semisolid or liquids other than breast milk) and ability to know the correct time to initiate complementary feeding (recalled 6 m after birth) | Farmer, housewife, private, or government | Government employee [AHR = 1.67, 95% CI: 1.10-2.53] was significantly associated with the time to initiate into complementary feeding; hazard of introducing CF among government employed was 1.67 times higher compared to housewives [AHR = 1.67, 95% CI: 1.10-3.53]. |
| 92 Rutagumba, 2021, Rwanda^184^ | Cross-sectional | Mothers with infants 6-12 m, n=221 | Early initiation; EBF | Employment measured as binary, type of work not specified | Employed mothers had lower odds of EBF (OR = 0.245, 95% CI: 0.16-0.62) |
| 93 Saaka, 2012, Ghana^94^ | Cross-sectional | Mother-child pairs (children 0-6 m) from health clinics, n=355 | BF, Colostrum, Giving Pre-Lacteals, EBF Rates | Unemployed, farming, salaried Worker, trader, pito brewing, others, unemployed, farmer, salary worker, petty trader | The strongest determinant of EBF was occupation being a trader. Higher percentage of EBF compared for farmers, salary workers, petty traders, but not unemployed. EBF was more common among petty trading and those on regular salary. Petty traders were 4 times more likely to practice EBF, compared unemployed. Salary worker and farmer were more likely to practice EBF, compared to non-working mothers. |
| 94 Siziba, 2015, South Africa^120^ | cross-sectional descriptive | Mothers/caregivers of infants (0-6 m) at health facilities in 4 provinces, n=580 | Six-month EBF rate: infants (in their sixth month of life) who received only breast milk on the previous day/total number of 6 m infants; BF initiation; EBF for 6 m; BF cessation | Didn't measure employment; return to work was reason for various IYCF practices | 9 (out of 32) identified working as a reason for not BF and 29 (out of 58) cited going back to work as a reason for BF cessation. 23 (out of 314) cited “return to work” as reason for giving infants other liquid/foods. |
| 95 Sobo, 2008, Nigeria^121^ | Cross-sectional | Nursing mothers selected from the list of clinic attendance, n=120 | looked at awareness of and practice of EBF and reasons for no EBF | Major occupation: farming (a few others in vocational work) | 11 (26.8%) respondents cited type of job as a reason for not practicing EBF. |
| 96 Sonko, 2015, Ethiopia^185^ | Cross-sectional | Mothers with children under 6 m n=420 | EBF, Prelacteal feeding, initiation of BF within one hour | Occupation: Student, Worker, Housewife/ unemployed; formal and informal work | Housewife/unemployed showed significant association with the practice of EBF (AOR=9.9;95% CI: 1.01 - 98.6) |
| 97 Teshale, 2021,  Ethiopia^162^ | Cross-sectional | Women of reproductive age who gave birth within two y preceding survey, n=14,672 | Pre-lacteal feeding practices | Type of work not specified; Occupation | Working women showed significant contribution to change (decrease) of pre-lacteal feeding practices |
| 98 Tewabe, 2016, Ethiopia^186^ | Cross-sectional | Mothers with children under 6 m, n=405 | EBF | Self-report via administered questionnaire; type of work not specified | 36.6% of employed mothers practiced EBF compared to 53.6% of their unemployed. Unemployed mothers were 3 times more likely to practice EBF than employed mothers (AOR=3.01, 95% CI: 1.46, 6.20). |
| 99 Tewabe, 2017, Ethiopia^187^ | Cross-sectional | Mothers with children under 6 m, n=423 | EBF | Quantitative survey compared EBF practice by sociodemographic categories, including employed vs unemployed; Self-report | Unemployed mothers are 3 times more likely to practice EBF (AOR=2.24, 95% CI: 1.16, 4.31) |
| 100 Thomas 2017, South Africa^30^ | Prospective birth cohort | Breastfeeding mothers from Drakenstein Child Health Study n=899 | BF practices (assessed at postnatal study visits at 6-10 wk, 14 wk, 6, 9, 12, 18, and 24 m): current feeding practices (time, frequency, type of food); EBF; BF initiation, duration, and cessation | Assessed via a questionnaire: employed/working or non-employed/not working. | Employment and HIV diagnosis predicted a lower likelihood of BF initiation and earlier BF-discontinuation; working mothers were more likely to never initiate BF (OR 2.60, CI 1.08, 6.24); Working, uninfected mothers were more likely to discontinue BF than unemployed (aHR 1.37, CI 1.10-1.784); Employment was a predictor of shorter BF duration and ethnicity a predictor of longer BF duration for uninfected mothers. For HIV-infected mothers' ethnicity and employment were not predictors. Younger age trended as a predictor for longer duration of BF. |
| 101 Tsegaw, 2021,  Ethiopia^188^ | Cross-sectional | Mothers with infants aged 0-6 m, n=1185 | EBF | Currently not working vs currently working | In bi-variable multilevel logistic regression maternal occupation currently working and community level of employment were associated with EBF. In multilevel logistic regression only community employment was associated with EBF. |
| 102 Ukwuani, 2003, Nigeria^189^ | Cross-sectional | Married women with living children at the time of survey, n=5331 | BF Duration and EBF | DHS; Other: Categorized by earning cash and if children came with them to work | Work reduced BF duration unless the mother earned cash and brought child to work (66.5% breastfeed). Mothers who earned cash but did not bring child to work had the shortest BF duration. |
| 103 Victor, 2013, Tanzania^190^ | Cross-sectional | Women (15-49 y) with children 0-23 m, n=10,522 | Early initiation of BF, EBF under 6 m, continued breastfeeding at 1 year, continued breastfeeding at 2 y, predominant breastfeeding, children ever breastfed, bottle feeding | mother's working status: working vs. non-working | Early initiation of infants 0-23 m was significantly lower among working mothers (43.2%) compared to non-working mothers (63.2%).  Exclusive breastfeeding of infants aged less than 6 m of age was significantly lower among mothers who had worked in the last 12 m (48.0%) compared to those who hadn't (58.9%). (p=0.035)  Odds for working mothers for delayed initiation of breastfeeding, non-exclusive breastfeeding and predominant breastfeeding = 2.3. (95%CI: 1.62, 3.29). |
| 104 Warille 2017,  South Sudan^191^ | Cross-sectional | Mothers with children aged 9 to 12 m attending the immunization and pediatric outpatient clinics, n=384 | EBF (practice and knowledge; EBF was defined as "giving only breast milk (and modern medicines only if prescribed)"); initiation of breastfeeding; use of prelacteal feeds; reasons for non-EBF | Occupation was categorized as housewife, salaried employee, self-employed, or student via a questionnaire | No significant association between occupation, age of the mother, mode of delivery and exclusive breastfeeding. Mothers tended to breastfeed longer where there was support from husbands or provision of work-based designated areas for breastfeeding. |
| 105 Woldeamanuel 2020,  Ethiopia^192^ | Cross sectional | Women who have births in the past five y prior the survey, n=5122 | early initiation, exclusive breastfeeding | Employment Status: (Not working, Working) | Working status was not significantly associated with early initiation and exclusive breastfeeding |
| 106 Yako,  2013,  South Africa^193^ | Cross-sectional | Convenience sample of 60 mothers in Community Health Centre's PMTCT program. Every mother who attended the PMTCT clinic six wk postnatally, irrespective of HIV status, was eligible if she had a singleton birth with a live, full-term baby, n=60 | socio-economic factors (availability of clean water, refrigerator, formula feed, income); presence of other family members in home; method of infant feeding communicated to clinic staff; maintenance of the method; additional substances given to the infant | Unemployed vs. focally employed full-time vs. employed on a part-time basis or informally vs. students  formal/informal | 50% of mothers who (n=8) changed their method of feeding changed it because they were going back to school, 25% (n=4) were going back to work. Mothers who changed their method of feeding were HIV-negative mothers. 100% of HIV-positive mothers maintained infant feeding method; 50% of HIV-negative mothers maintained infant feeding method. |
| 107, Yeboah  2019,  Ghana^194^ | Cross-sectional | Mothers of children 6-24 m, n=160 | EBF | Quantitative survey compared EBF practice by several sociodemographic categories, including occupation (employed/unemployed)  Self-report, not specified | Unemployed mothers were 1.2 times more likely to practice EBF as compared to their employed counterparts (AOR=1.20, 95%CI: 0.27, 0.89). |
| 108 Yeheyis, 2016, Ethiopia^195^ | Cross-sectional | Women with children 6-12 m, n=398 | Complementary feeding | Quantitative survey compared sociodemographic categories: employed vs unemployed; Proper complementary feeding (providing nutritionally adequate foods at 6 m along with breast milk); Self-report | 87% of employed mothers were more likely to start complementary feeding at the wrong time, 54.1% of employed mothers began too early (AOR=0.138, 95%CI (0.045, 0.418)). Mothers who started complementary feeding before six m (n=134), 14.9% (n=20) reported long work hours as primary reason. |
| 109, Yimer 2021,  Ethiopia^196^ | Community-based cross-sectional study | Mothers of children 6-24 m, n=634 | EBF | Type of work not specified; Employed vs not employed | Reasons for not practicing EBF |

**Supplemental Table 4.** Results of work-focused and work-included mixed-methods studies

| **Author, Year, Country** | **Data collection methods** | **Population** | **Infant feeding measurement** | **Employment measurement** | **Findings that related work and breastfeeding** |
| --- | --- | --- | --- | --- | --- |
| ***Work focused mixed-methods studies*** | | | | | |
| 1 Luthuli, 2020, South Africa  ^135^ | Questionnaires and in-depth interviews | Mothers with infants less than 1 year who returned to informal work (n=18) | Initiated breastfeeding, exclusive breastfeeding, formula feeding, mixed feeding | Characteristics of work and child care; type of work not specified | Quantitative: No results reported related to work and breastfeeding.  Qualitative: Return to work was a key reason for changing feeding practices within two weeks post-delivery. Quantitative: No results reported related to work and breastfeeding. |
| 2 Nkrumah 2021, Ghana ^197^ | In-depth interviews and questionnaires | Breastfeeding health workers in Effutu Municipality | Not measured | Workplace supports; clinical staff or support staff | Quantitative: No results reported related to work and breastfeeding.  Qualitative: Breastfeeding health workers reported maternity leave, spousal support, and coworker support. Participants reported the lack of breastfeeding policy in hospitals. Feeding strategies mothers used were expression (34%), breastfeed when home (40%), breastmilk and porridge (26%). |
| 3 Nkrumah 2017, Ghana^50^ | Focus group discussions and cross sectional survey | Qualitative: Mothers of infants 0 to 6 m (n=35)  Quantitative: Mothers of infants 0 to 6 m (n=225) | Early initiation, exclusive breastfeeding, breastfeeding frequency | Formal employment classified as paid jobs and/or situations where mothers work in formal organizations such as schools, hospitals, banks, factories and supermarkets.    Informal employment: included mothers who were self-employed; usually in small enterprises such as, subsistence farming, dressmaking, hairdressing, trading, catering, and other forms of self-employment. | Quantitative: 84% of informally employed mothers practiced EBF compared to their formally-employed counterparts (16%), (p = 0.020).  Maternal occupational type was related to EBF frequency. More informally employed women (91%) breastfed 8 or more times per day compared to formally employed mothers (9%) (p=0.021).  Mothers who went to work with their children (64%) breastfed 8 or more times daily compared to those who did not (36%) (p=0.000).  Qualitative: Flexibility of maternal work was identified as a helpful BF support along with scheduled feeding and family support.  Mothers who worked in hazardous conditions and who maintained EBF had the flexibility to leave their children in the care of relatives or older siblings. |
| ***Work-included mixed-methods studies*** | | | | | |
| 1 Adeniyi, 2019, South Africa^33^ | Prospective cohort | Women living with HIV (n=1709) | The decision to exclusivelybreastfeeding - compared to exclusive formula feeding (one variable with the two categories). | Employment status defined as unemployed vs employed | Quantitative: Chi-square test demonstrated an association between EBF and employment status but not in adjusted analysis.  Qualitative: reasons for infant feeding choices for women with HIV are returning to work, looking for employment, and farm work is too much and won't be able to stay with the baby. These were under the "work/school-related" theme. These women mostly preferred formula-feeding. These women mostly preferred formula-feeding because they were returning to school or work, or looking for work |
| 2 Agunbiade, 2012, Nigeria^100^ | Complementary mixed method design | Breastfeeding mothers (Yoruba mothers with an infant between 3 m-1 year of age; seeking postnatal care at the health center) (n=200 breastfeeding mothers completed the questionnaire; 11 mothers participated in in-depth interviews) | Questionnaire: "breastfeeding knowledge, intention, and factors encouraging or discouraging breastfeeding mothers from the practice of exclusive breastfeeding...knowledge about breastfeeding initiation, duration, infant nutrition, and challenges of breastfeeding"; in-depth interviews discussed breastfeeding experiences and support; reported awareness of breastfeeding, intention, EBF practice, frequency of breastfeeding, length of breastfeeding, supplementation, reasons for breastfeeding, breastfeeding experiences, constraints + challenges of breastfeeding, reasons for stopping EBF, and breastfeeding initiation | Mothers responded to a questionnaire and the following categories were listed: artisan, employed in private sector, employed in public sector, self employed, student, trading  formal and informal work | Quantitative: returning to work/business was cited by 28 (24%) of the women for discontinuation of EBF.  Qualitative: No results reported that relate work and breastfeeding. |
| 3 Alabi 2020  Nigeria^34^ | Cross-sectional survey design, and mixed method of data collection (in-depth interviews) | Nursing mothers with babies 7-12 m (n=538 quantitative and 40 qualitative) | The dependent variable was operationalised by whether the respondents breastfed their infants with only breast milk during the first 6 m of birth. We asked, ˜What did you feed your child with when he/she was between ages 0 and 6 m with options, “breastmilk only”, “complementary breastfeeding”, “infant formula only”, “pap only”, and “solid foods”. Those who chose only the first option were regarded to have practiced EBF. | employment status during the first 6 m after delivery (employed/unemployed)  type of work not specified | Quantitative: No significant association between mothers' employment status and practice of EBF.  Qualitative: highlighted the importance of the nature of a mother's work in relation to EBF, particularly length of maternity leave and ability to bring baby to work. |
| 4 Andare 2019 Kenya^35^ | Cross-sectional | Mothers living with HIV with infants 0-12 m old at the time of the study attending PMTCT clinic at the Kiambu Level 4 Hospital. (n=180) | Children 0-5 m; Exclusive breastfeeding, replacement feeding and mixed feeding  Children 6-12 m; complementary feeding with continued breastfeeding and complementary feeding with no breast milk | Compared EBF by the occupation categories. Did the same with complementary feeding with breastfeeding (children 6-12 m) by occupational categories.  Employed, unemployed, casual labor  type of work not specified | Quantitative: Though not statistically significant, mothers who were employed (OR=1.36) and unemployed (2.17) were more likely to practice exclusive breastfeeding compared to those on casual labor.  Qualitative: No results reported that related work and BF. |
| 5 Bekere 2014, Ethiopia^163^ | Focus group discussions and cross sectional survey | Mothers living with HIV with children aged 0-6 m visiting selected health institution of West Oromia (n=118) | Initiation of breastfeeding, EBF, replacement feeding, mixed feeding | housewives, merchants, civil servants and farmers  formal and informal work | Quantitative: Being a merchant and working far from home were barriers to EBF. Those who work far from their homes were .0348 less likely to practice EBF.  Qualitative: No results reported that relate work and breastfeeding. |
| 6 Burns, 2016, Democratic Republic of Congo^101^ | Formative research | Women of reproductive age, mothers with children aged less than two y and health care providers (FGD=188  Structured interviews=119) | Structured interviews among mothers of infants <24 m of age were also conducted, with a questionnaire focused on IYCF knowledge, attitudes, and practices. IYCF measured are initiation of breastfeeding, EBF | Categorized as source of income: Fishing, small business, temporary wages, spouse, salaried job, relative, rent, handcraft, agriculture, collects firewood, enterprise/working for others and cooks | Quantitative: No results reported that relate work and breastfeeding.  Qualitative: Working in the fields affected EBF practice. For example, a mother reported feeding her crying infant porridge when she returned home from being in the field all day. |
| 7 Demelew, 2014, Ethiopia^102^ | Cross-sectional, qualitative and quantitative study | WRA with children 0-2 y (n=356) | Prelacteal feeding, EBF for 6 m | Government worker, private company worker, daily laborer, housewife  formal and informal work | Quantitative: Inconvenient working hours was reported as a challenge to BF among 36%; sixty-nine per cent of mothers who are housewives practice exclusive breastfeeding, but a significant association was not found when this was compared with laborers.  Qualitative: No results reported that relate work and breastfeeding. |
| 8 Jama, 2017, South Africa^37^ | Longitudinal cohort | Qualitative: In-depth interviews among mothers who planned to EBF for 6 m recruited during pregnancy, purposively sampled to include working women, teenagers, and HIV positive pregnant women (n=22)  Quantitative: Same sample as above recruited for surveys (n=125) | infant feeding practices (using in-depth interviews and 24 h feeding recall): EBF or having introduced foods | Reported how many mothers had "current paid work"; also looked at additional income sources that included "mother receives money from employer" and "mother receives money from self-employment" | Qualitative: Returning to school and work was cited as a barrier to EBF. For example, the infant being given sugar water or the mother struggling to express breastmilk and therefore introducing formula were cited.  Expressing breastmilk and staying with the infant for 6 m were cited as helping to maintain exclusive breastfeeding status while working.  Quantitative: No results reported related to work and breastfeeding. |
| 9 Kiarie, 2004, Kenya^38^ | Cohort and FGD | Pregnant women living with HIV recruited at 36 wk, follow up at 1 and 6 wk (n=128) | Not breastfeeding, EBF, mixed feeding [information on infant feeding practices and on the reasons for choosing different modes of infant feeding was obtained at 1 and 6 wk. At these visits, women were asked how they were currently feeding their infants (previous 24 hours)] | Employed/not employed  Not specified | Quantitative: occupation of the woman was not associated with infant feeding practices (but maternal employment was a predictor of poor infant growth)  Quantitative: No results reported related to work and breastfeeding. |
| 10 Kumeh, 2020, Liberia^128^ | In-depth interviews | Mothers of malnourished children and non-malnourished children aged between 0 and 2 y (n=100) |  |  | Quantitative: No results reported that relate work and breastfeeding.  Qualitative: Mothers gave returning to school or vocational training as a reason separating them from their infants and not being able to breastfeed.  Mothers discussed the economic burdens of being the primary family breadwinner and a caregiver.  Mothers were not able to care for their infants as often as needed due to jobs and informal work with poor support in terms of leave, pay, security, accommodations, and childcare support. This necessitated early initiation of complementary feeding. Searching for work caused mothers to be separated from their infants, hindering breastfeeding practices. |
| 11 Melese Ayele 2021, Ethiopia^103^ | Questionnaires and focus group discussions | Mothers with children aged less than 24 m (n=423; 6 FGD) | EBF to 6 months duration | Formal and informal work, work categories included housewife, employee, and merchant | Quantitative: Employed mothers were 78% less likely (AOR = 0.22, 95% CI: 0.16, 0.56) to practice EBF compared to unemployed mothers and housewives.  Qualitative: Work was cited as a barrier to exclusive breastfeeding. This work included outdoor farming, causing women to need to walk far distances and to leave their infant in the care of other children or grandparents. While the mother is away at work, the infant may be given complementary foods such as eggs, cow’s milk, and fruit.  Despite being aware of the importance of EBF, mothers reported not being able to EBF due to economic status. |
| 12 Moshy, 2013, Tanzania^129^ | Cross-sectional | Quantitative: Mothers with children < 5y enrolled in Kindergarten and brought to Reproductive and Child Health clinics in Chole and Jibondo (Chole n=52 Jibondo n=104 Total n=156)  Qualitative:  Interviews, focus group discussions, personal observations, and informal talks  Chole: Interviews with women, men, kindergarten teachers, and healthcare officials (n=10); FGD with men, women, and kindergarten cooks (n=4 FGD)  Jibondo: Interviews with women, caregivers, men, and healthcare officials (n=14); FGD with men and women (n=5 FGD) | Qualitative questions about breastfeeding, introduction of substitute foods | Qualitative questions about income-generating activities (food production, rope making, handcrafts, farming all mentioned)  Other: income generating activities particularly seaweed farming and octopus picking | Quantitative: No results reported related to work and breastfeeding.  Qualitative: Most mothers in Chole still breastfed their children as in the past because their important activities for generating income and food production, rope making, handcrafts and farming, took place around their homesteads. In Jibondo, the frequency and duration of breast-feeding among the under-fives had reduced substantially because mothers resumed seaweed farming and octopus fishing soon after delivery. Most mothers resumed their livelihood activities soon after 40 days.  Cases were reported of mothers resuming seaweed farming and octopus fishing in less than a month after delivery. An extreme case was reported of one mother who resumed her fishing activities within a week after she had delivered, and the child was found to be underweight. However, there were still some mothers who waited up to eight m before they resumed their activities. |
| 13, Muko 2004, Cameroon^104^ | cross-sectional | Mothers who participated in the PMTCT program (had been counselled, tested positive, administered nevirapine) and delivered live babies at least 3 m prior to the study. (n=104) | Choice of Feeding: Artificial milk vs. breastfeeding | occupation of mothers (housewives vs. farmers vs. teachers vs. students vs. other civil servants vs. no occupation) | Quantitative: Authors report EBF frequency by occupation type: Housewives: 81% breastfeeding  Farmers: 82% breastfeeding  Teachers: 91% breastfeeding  Students: 85% breastfeeding  Other civil servants: 100% breastfeeding  No occupation:67% breastfeeding  12.5% note “job” as a barrier to breastfeeding.  Qualitative: No results reported related to work and breastfeeding. |
| 14 Mushaphi, 2017, South Africa^130^ | Nested qualitative study was conducted among mothers in an ongoing birth cohort study | Quantitative (cohort): newborns who weighed at least 1500g with no congenital conditions and were less than 17 d.  Qualitative: 37 caregivers participated in FGD (4 FGDs with 7-10 caregivers each). | Breastfeeding initiation after birth (included, within 1 and 24 hours, and 3 days after delivery). Colostrum feeding and exclusive breastfeeding to 6 m after birth. | Type of work not specified | Quantitative: No results reported related to work and breastfeeding.  Qualitative: Mothers cited going back to work or school as one of the main reasons for introducing complementary feeds early than 6 m.  Returning to school or work was also the main reason for stopping breastfeeding before 2 y. |
| 15 Nwankwo, 2002, Nigeria  ^113^ | Cross-Sectional | Mothers with children between 4 and 28 m (n=411) | EBF | Quantitative survey compared EBF practice by several sociodemographic categories, including employment. Qualitative interviews explored experiences with EBF or complementary feeding.  Self-report, formal and informal work | Quantitative: Among employed mothers, those in skilled employment were more likely to practice EBF (15.9%) compared to those in an unskilled occupation (8.5%) (p=0.045).  Qualitative results: None results reported that related work and breastfeeding. |
| 16 Okafor 2022 Nigeria^198^ | Cross sectional and IDIs | 1409 mothers | Level of compliance to EBF was categorized as high or low based on the responses to 10 items,on the questionnaire | Occupation (i.e. civil servant, trader, farmer, self-employed, housewife student) | Quantitative: No results reported related to work and breastfeeding.  Qualitative: Unfavorable conditions at the workplace affected infant feeding practices. |
| 17 Safari, 2013, Tanzania^57^ | Cross-Sectional | Women attending Reproductive Child Health Services facilities in Morogoro (n=130) | Early Initiation, early introduction of other foods and liquids, EBF | Semi-structured questionnaire collected demographic data and attitudes about breastfeeding, compared BF practice by several sociodemographic categories, including employment.  Self-Report, Formal and Informal employment | Quantitative: Self-employed or unemployed women were up to 5 times more likely to practice EBF than their formally employed counterparts (p<0.05).  Qualitative results: None results reported that related work and breastfeeding. |
| 18 Trafford 2020, South Africa ^40^ | Survey and semi-structured interviews | Mothers registered in MomConnect reporting infant feeding practices during the first six m (n=115) | Exclusive breastfeeding, Mixed feeding and no breastfeeding | Employed vs unemployed | Quantitative: No results reported related to work and breastfeeding.  Qualitative: Needing to return to work was identified as a factor that could be important when deciding breastfeeding intentions. Mothers who had to leave their infant with family when returning to work were cited to have low decision-making power.  Returning to work (either planned or forced) was identified as a primary reason for mixed feeding at less than 6 month of age or introducing formula. The need to return to work also affected the timing of beginning complementary feeding.  Maternity leave was identified as only enabling breastfeeding until the leave period was over. |
| 19 West 2019, South Africa^131^ | In-depth interviews and cross-sectional survey | Quantitative: All women attending antenatal or postnatal care at Witkoppen between July 7, 2015 and March 6, 2018 extracted from the Witkoppen's electronic FRESH start database. n=8116  Qualitative: Mothers living with HIV on ART with a child < 12 m, breastfeeding or formula feeding. Healthcare providers engaged in ante-and postnatal care (nurses, lay counselors, health educators) (n=34; 22 mothers and 12 healthcare providers) | Feeding choice (breastfeeding vs. formula feeding) | Work category not measured. Discussed as a topic in interviews. | Quantitative: No results reported related to work and breastfeeding.  Qualitative: Employment influenced feeding choice. One mother said she chooses to breastfeed because she is not working and does not have money to buy formula. Concerns that family members or daycare providers would feed something other than breastmilk led some women to choose formula feeding instead of breastfeeding. |
| 20 Yeneabat, 2014, Ethiopia^199^ | Cross-sectional | Mothers of index infants less than 12 m old (n=592) | EBF (cessation), complementary feeding | Self-report, women were classified by specific occupation (see above)  Other: Mothers categorized by specific occupation (Housewife, farmer, merchant, civil servant, student, other) | Quantitative: Returning to work was listed as the most common main reason for the introduction of complementary foods before 6 m (30.8%).  Mothers engaged in occupations such as day laborers, house servants, and pottery makers were 5.16 times more likely to terminate EBF early compared to housewife mothers (AHR: 5.16, 95%CI (1.02, 26.12)).  Civil servant mothers were 1.72 times more likely to cease EBF early compared to housewives (HR-1.74, 95%CI (1.27, 2.40). Result was not significant in adjusted models.  Farmers were less likely to stop EBF early compared to housewives (HR=0.77, 95%CI (0.60, 0.99)). This also lost significance upon adjustment.  Qualitative: The highest proportion of introduction of complementary foods occurred during the first month of the infant's life, after the time of baptism, which is when mothers often return to work. |
| 21 Zulliger, 2013, South Africa^132^ | Convergent parallel, mixed methods study | Women diagnosed with HIV and were currently pregnant and/or had delivered a live baby within the past 2 y. Pregnant and postpartum women purposively invited to participate in qualitative study. [Quantitative (n=406)  Qualitative (n=34, 12 post-partum, 22 pregnant)] | Intention to breastfeed for pregnant women (plans to breastfeed vs. plans not to breastfeed), ever breastfed vs. never breastfed for postpartum women | Not really measured, just discussed briefly in qualitative interviews | Quantitative: No results reported related to work and breastfeeding.  Qualitative; Women who were working or looking for work frequently reported that breastfeeding was not feasible because others were involved in the care of the infant who might provide the infant with food and other liquids. Preferred formula feeding because it gave them more flexibility in what they fed the baby and in childcare. |

Abbreviations: AOR: Adjusted odds ratio; OR: Odds ratio; EBF, exclusive breastfeeding, EIBF, early initiation of breastfeeding

**Table 5 Reviews that report work and breastfeeding in Africa**

| **First author, year, country** | **Type of review** | **Number of Articles included** | **Breastfeeding measures** | **Work measures** | **Results** |
| --- | --- | --- | --- | --- | --- |
| ***Work-focused systematic reviews*** | | | | | |
| Habte 2022, Ethiopia  ^36^ | Systematic review and meta-analysis | 24 articles | Exclusive breastfeeding | Employment status | Maternal employment status was significantly associated (POR=0.51, 95% CI 0.16, 0.86) with EBF; employed mother were less likely to practice EBF |
| Wake 2021, Ethiopia  ^200^ | Systematic review and meta-analysis | 45 articles | EBF to 6 m | Maternal employment | Full-time maternal employment was negatively associated with EBF in comparison to unemployed mothers (OR 0.43; 95% CI: 0.31, 0.61). |
| ***Systematic reviews that include work and breastfeeding*** | | | | | |
| Ejie 2021, 11 countries in sub-Saharan Africa  ^201^ | Qualitative systematic review | 20 articles |  |  | Maternal employment was the most common barrier to EBF |
| Gyamfi, 2022, Ghana  ^202^ | Scoping review | 15 articles | EBF practices, barriers, facilitators, workplace factors | Maternal work (experience, type, rank, location, risks to infant health, going to work with the infant, hours worked, and distance from home to workplace) | EBF for 6 months was more prominent among mothers who were unemployed or worked in the informal sector. Formal sector mothers lacked supportive workplace policies |
| Habtewold 2019, Ethiopia  ^150^ | A systematic review and meta-analyses | 70 articles | Timely initiation of breastfeeding (TIBF), exclusive breastfeeding, and timely initiation of complementary feeding. | Maternal occupation: government-employed vs unemployed | Maternal occupational status significantly associated with low EBF practice, but not TIBF. The pooled estimate showed that women who were government employed had lower odds of TIBF (OR=0.82, 95% CI 0.59-1.13), though this association was not statistically significant.  The pooled estimates showed significantly lower odds of EBF among government-employed women (OR=0.60, 95% CI: 0.40, 0.91). |
| Nieuwoudt 2019, South Africa  ^39^ | Mixed-methods systematic review | 72 articles | EBF up to 6 m. | Employed vs unemployed | Common EBF barriers included unsupportive workplaces. Shorter EBF duration observed among employed mothers. Young and unemployed mothers were vulnerable to stopping EBF because of gendered cultural expectations in their households and low perceived power. Older mothers faced their own challenges. Those who were employed or looking for work opted for formula or mixed feeding. |

**Abbreviations**: POR: Pooled odds ratio; OR: Odds ratio; EBF, exclusive breastfeeding, TIBF, timely initiation of breastfeeding

**Supplemental Table 6.** Descriptions of qualitative studies

| **First author, year, country** | **Data collection methods** | **Participant description, n** | **Key findings related to work and infant feeding** |
| --- | --- | --- | --- |
| ***Work-focused qualitative studies (n=10)*** | | | |
| Gebrekidan, 2020, Ethiopia  ^203^ | Interviews | Mothers with infants less than 12 m, full-time formal work (n=20) | - Breastmilk expression uncommon and most considered it inappropriate - Described the challenge of managing EBF, childcare, household activities and work |
| Horwood 2020, South Africa  ^204^ | Focus group discussions (FGD) | Women with children aged less than 5 y (n=87, in 14 FGDs) | - On returning to work many mothers changed their infant feeding practices, including adding breastmilk substitutes. Some mothers continued to breastfeed albeit not exclusively. Others shortened the duration of any breastfeeding or ceased breastfeeding altogether - Unable to afford breastmilk substitutes they gave tea - Several women expressed breastmilk |
| Ickes 2021, Kenya  ^142^ | Interviews | Mothers who were employed at a commercial flower farm or hotel and had a child younger than 12 m (n=42) | - Work poses numerous obstacles to practicing EBF after returning from maternity leave: inability to travel home or to daycare to breastfeed during workday, lack of knowledge about and experience with milk expression and storage, and lack of EBF spaces and pumping equipment - Employed mothers reported going back to work necessitates early introduction of other foods. Early introduction of foods like porridge and cow's milk would help child adjust to a new diet before they return to full time work. - Few employed mothers reported expressing breast milk, despite wanting to; they perceived it to be infeasible due to lack of instruction on how to do so and lack of a hygienic environment and equipment. - Mothers indicate that on-site lactation rooms would enable expression and storage at their workplaces, but were concerned over pesticide exposure. - Mothers were concerned that their expressed milk was appropriately stored and reheated at daycare and given to the appropriate child. - Mothers indicated that they would be willing to express breastmilk for their baby if they were provided breast milk instruction, a place to store the milk, a breast milk pump, and a private lactation room. |
| Mabaso 2020, South Africa  ^143^ | Semi-structured interviews | Senior managers and employed mothers (n=12) | - Lack of space to express and store breast milk at work - Challenges dealing with conflict between work demands and infant breastfeeding needs (stress and discomfort from full breasts) - No recognition that a mother returning to work may be breastfeeding and have both work and breastfeeding duties |
| Mabaso 2022, South Africa  ^205^ | In-depth interviews | Mothers who are teachers (n=13), principals (n=14) | - Breastfeeding was incompatible with teaching, lack of facilities and time - Common to stop breastfeeding during leave in preparation for returning to work - Mothers perceived principals to be unsupportive of breastfeeding and were uncomfortable asking for workplace support |
| Maponya 2021, South Africa  ^206^ | Semi-structured interviews | Breastfeeding mothers (n=8) | - Mothers need to return to the workplace soon after birth - There is little support from employers and coworkers for exclusive breastfeeding, workplace policies are not fully implemented, and workplaces are not supportive of expressing and storing milk |
| Mlay, 2004, Tanzania  ^144^ | Interviews | Mothers 25-30 y old and breastfeeding babies less than 7 m old, (n=6) | - Workplace flexibility, despite its importance, was reported to be widely variable. - Mothers are being forced to choose between employment and their children. |
| Nkrumah 2020, Ghana  ^140^ | In-depth interviews | Professional working mothers in the formal sector in Accra, Ghana (n=20) | - Workplace environment is not conducive enough for mothers to exclusively breastfeed their babies. - Two major items identified as promoting exclusive breastfeeding were maternity leave and early closing hours for nursing mothers. - Factors promoting EBF included maternity leave and early closing hours. Factors hindering EBF included absence of maternity policy in organizations, inadequate institutional support (ex. lack of breastfeeding rooms and breaks in the day to breastfeed), and work-family imbalance |
| Stumbitz 2020, South Africa  ^207^ | In-depth interviews | Mothers with a child born after 2011 and who also have experienced working during pregnancy and returned to work after maternity leave (n=51) | - Participants were informed of workers' rights to workplace breastfeeding support. Employees shared that this knowledge would have increased their likelihood to ask for support. - Mother started preparation soon after she gave birth by introducing formula feeding alongside breastfeeding. - Expressing breastmilk at work was reported. - Breastfeeding for six m was not possible because of returning to work. |
| Wolde 2021, Ethiopia  ^208^ | In-depth interviews | Employed mothers who received maternity leave or had on-site child care | - Returning to work at 3 m was a barrier to breastfeeding - Mothers with workplace accommodations and support reported greater satisfaction with their job and better breastfeeding practices |
| ***Work-included qualitative studies (n=21)*** | | | |
| Afolayan 2020 Nigeria, ^209^ | Focus group discussions | Mothers of infants 6 m | - Mothers knew EBF for 6 m was recommended but introduced other foods at 3-4 m because government-employed mothers needed to resume work after maternity leave |
| Agyekum  2022, Ghana  ^210^ | Semi-structured interviews | Mothers of infants 0-5 m, health care workers | - Type of work influences EBF. Formal settings are not conducive to EBF because of separation and lack of private space. Mothers in informal settings are able to be with their children and exclusively breastfeed longer |
| Ahishakiye 2019, Rwanda  ^211^ | Focus group discussions | Four key informant groups: mothers and fathers of infants aged 0-23 m, grandmothers, and community health workers (n=144; 16 FGD) | - It was reported that some mothers, particularly those that earn their livelihood from daily labor, breastfed their infants simultaneously while doing manual work. For example, it was noted that mothers bring their infants during farming activities, but might not be able to breastfeed on demand out of concern for losing her job. |
| Coetzee 2017, Nigeria  ^122^ | In-depth interviews | Mothers were interviewed in their third trimester of pregnancy and again post-partum (n=37; 25 mothers living with HIV, 12 mothers not living with HIV) | - During the third trimester, many participants stated that attending work would not influence their ability to EBF. They would either take the infant with them to work or express milk. - One month post-partum, some women stated that EBF posed some challenges as they tried to juggle both work and breastfeeding. For example, long work hours prevented some participants from finding time to go home during the day to feed their children. |
| Cuinhane 2017, Mozambique  ^41^ | In-depth interviews (0-6 m), direct observation of feeding practices, and focus group discussions | Lactating mothers living with HIV with children 0-2 y (n=59) | - Participants presented that one of the reasons for practicing mixed feeding was returning to work and school. - Participants said practicing exclusive breastfeeding up to six m was a challenge because most of them had to return to work 3-4 m after childbirth. Farming days lasted 4-6 hours, and during that time, they left their babies at home. |
| Doherty 2020, South Africa  ^123^ | Focus group discussions | Mothers with infants ages less than 6 m (n=67) | - Few women described expressing breastmilk to leave for their infants while at work or school. - Mothers’ concerns were related to not being able to express sufficient milk for the day and fears that family members would give other foods in addition to the expressed breastmilk. They reported having very little control over what their infants were fed while they were away. - Some mothers reported hiding their BF behaviors if they BF non exclusively for fear of reproach from HCWs. |
| Goon 2020, South Africa  ^42^ | Semi-structured interviews | Mothers living with HIV, 18 y and above who reported to have introduced complementary feeding to their infants before six m, N=319 | - A few women had to introduce formula feeding gradually after a month so that the child would adapt to the mother’s absence when she returned to work or school. - Employed mothers have difficulty practicing exclusive breastfeeding because of work-related challenges. They have to resume work as stipulated in their leave notification. They have no option other than to leave the baby at home with other relatives. In some cases, they are compelled to stop breastfeeding due to the long hours at work and work-related travel. |
| Hunter-Adams 2016, South Africa  ^124^ | Semi-structured interviews and focus group discussions | Women who had arrived in Cape Town within the last 5 y, who were over 18 y old, and with children 0-2 y old (n=23 for IDI; n=48 for FGD; total n=71) | - Work was an important stressor that inhibited the mothers' abilities to breastfeed. - Mothers report that they do not eat enough because of stressors, including employment, causing milk insufficiency. |
| Kimani-Murage 2015, Kenya  ^212^ | In-depth interviews, focus group discussions, and key informant interviews | Women of reproductive age who were pregnant, breastfeeding, or with children under 5, community leaders, healthcare professionals, community health workers, and traditional birth attendants (n=110; 20 men and 90 women) | - Work lasts too long to bring children, so they are left behind with siblings, relatives, neighbors, or (substandard) day care centers. These centers will introduce other foods too early, resulting in malnourishment. - Expressing breast milk is uncommon, sometimes culturally unacceptable. Formula is too expensive, so the only other choice is a substitute, such as milk or porridge, introducing it as early as 1 month. |
| Matare 2019, Tanzania  ^44^ | In-depth interviews and focus group discussions (only with fathers) | mothers with infants 0-5 m (n=36) | - Over half of mothers reported that heavy workloads, including farming and gardening, limited their ability to breastfeed optimally. - Long distances between the home and the farm, the physical demands of farm work, and other work requiring long periods of time away from the baby made it particularly challenging to breastfeed frequently, on-demand, or when the baby cried. - Some mothers reported that during farm work they left the baby at home in the care of others for several hours at a time. Other mothers brought the baby to the farm with them, but they were unable to take time to breastfeed while farming. Some mothers reported that cooking and fetching water also inhibited breast-feeding. - After trying related recommendations, several fathers and mothers perceived women having more time to breastfeed negatively. |
| Mgongo 2018, Tanzania  ^59^ | Focus group discussions | Women with infants aged 0 to 12 m who were still breastfeeding (n=78) | - Mothers felt that it is difficult to practice EBF because of the challenges they are facing, such as the need to return to work. - Most mothers had the responsibility of feeding their families; hence, they needed to resume working soon after delivery. They said that they were typically working at the farm-land â€˜shamba' for the whole day before returning home in the evening. - Pressures that the mothers in the informal sectors faced when returning to work that causes them to compromise EBF practices. Mothers working in the formal sectors had similar challenges (the need to return to work), as maternity leave is only three m. - In some situations, the mothers who were working in a formal sector shared that they were denied the 2 hours for breastfeeding though the national Policy of Tanzania allows for mothers to breastfed 2 hours per day until the baby is 6 m of age. |
| Mulindwa 2004, Uganda^136^ | In-depth interviews and focus group discussions | Mothers with children under 2 y (n=not specified)  5 FGDs (11 members) | - Respondents felt that working away from home deprived them of access to their children and limited their ability to breastfeed on demand. This constraint led to the early introduction of other foods. - Working away from home and the expense of childcare often forces mothers to leave the children with relatives who might not listen to instructions or might not be able to care for the child the way that the mother wants. - Respondents reported a desire to bring their children to work, but cited working conditions or the nature of their jobs as inhibitors. - Mothers reported being unable to check up on their children during the workday because of the distance to their houses. If they left, they could miss customers. |
| Omer-Salim 2007, Tanzania  ^125^ | Semi-structured interviews | Mothers of children 0-6 m of age and currently breastfeeding (n=8) | - Participants described how feeding the baby, housework, and paid work have to adjust to each other. |
| Operto 2020, Uganda  ^213^ | Focus group discussions | Women living with HIV at Kawempe Home care, whose youngest child is aged between six m and two y and the women aged above 18 y (n=20) | - Return to work was cited as a reason for not EBF for six m. It was noted that many workplaces do not allow mothers to bring their infants and that some partners may wish for the infant to stay at home and be mixed fed. |
| Otoo 2009, Ghana  ^45^ | Focus group discussions | Women recruited from 3 antenatal clinics which offered voluntary counseling and testing for HIV while pregnant with index child under 4 m old at the time of the study (n=4 FGDs with 7-10 women) | - In general, participants reported working mothers would not have enough time to breastfeed their infants exclusively either because of the short maternity leave or the inability to find a convenient feeding location. This was especially the case for mothers who hawk their products or are busy trading in the market. |
| Samburu 2021, Kenya  ^126^ | Focus group discussion, in-depth interviews, key informant interviews | Mothers living with HIV and not living with HIV who had a child under age 2 y, community leaders, religious leaders, traditional birth attendants, herbalist, health administrators, community health volunteers (CHVs), healthcare providers, mothers, fathers, grandmothers who were residing in Koibatek (n=205) | - Employment was reported to be a challenge for breastfeeding mothers because mothers often spend a long time away from the infant and most workplaces do not offer lactation rooms or any support policies for breastfeeding. |
| Sewannonda 2022, Uganda  ^214^ | Focus group discussions and key-informant interviews | Parents and healthcare and public health professionals | - Breastfeeding practices are influenced by work-life balance and employment demands |
| Stumbitz 2018, Ghana  ^58^ | Semi-structured interviews and focus group discussions | Employers and employees from differently-sized firms in different economies (small, medium, large firms;)  (n=29 employers, n=34 employees) | - Many workplaces comply with or even exceed statutory entitlements, such as with maternity leave, but they still do not cater to mothers' needs by not allowing children in the workplace, assigning breastfeeding breaks at the end of shifts, or not allowing for flexibility in the mothers' schedules. The mothers' most immediate needs, like facilitating access to their children, are not met, even though the employers are meeting mandated MP provisions. - Small, formal economy businesses were found to have better workplace supports, including the ability to bring children to the workplace, allowing for easier breastfeeding. - Breastfeeding in public spaces and bringing children to work is common in the informal economy, and that proximity to their children allows mothers to breastfeed whenever the child needs. The staff in these workplaces can have familial bonds that facilitate this support, but those take time to form, and those supports are often not extended to newer employees. |
| Talbert 2018, Kenya  ^46^ | Focus group discussions | Mothers with newborns (n=50) | - Mothers reported breastfeeding on demand and leaving work or school to return home to breastfeed. - Either expressing breastmilk or returning home during the day were identified as means of maintaining breastfeeding while working away from the home. |
| Tampah-Naah 2019, Ghana  ^47^ | In-depth interviews | Mothers aged 15-49 who were residents of the selected sites and who had given singleton birth to a child currently aged 0-23 m (n=20) | - Work schedule was reported to interfere with EBF practice, especially since the mother cannot always bring the child along to do work. The mothers are forced to leave their child with a caretaker while they work, causing them to be unable to feed them. - Mothers do not have enough time during the day to breastfeed, leading to the introduction of other foods while the mother is away. - Returning early to work (3 m of maternity leave) was reported to be a challenge to mothers trying to practice EBF. |
| Van der Merwe 2018, South Africa  ^48^ | In-depth interviews | Mothers living with HIV (aged 18-29 y) from Gauteng Province, South Africa with a baby between one and six m old and enrolled in an MTCT program (n=8) | - Practicing breastfeeding was influenced by work demands and personal preferences. - Women with employer or partner support may be more likely to exclusively breastfeed than single mothers and others lacking such support. |
| Wainaina 2018, Kenya  ^215^ | In-depth interviews and focus group discussions | Qualitative IDIs (n=3 stay-at-home mothers and 6 working mothers) and FGDs (n=4 stay-at-home mothers and 8 working mothers) | - Participants reported that they received inadequate workplace support, citing returning to work after maternity leave as a major challenge. - Mothers reported that their schedules did not allow adequate time to breastfeed during the day, so they were forced to wake up early to express. Some women expressed at work in unsuitable locations (office, car, bathroom) as a result of not having designated expression facilities. They also did not have access to dedicated refrigeration, opting to use a common refrigerator or leave it in the car and worry about it spoiling. - Mothers also reported that work-related stress and reduced BF frequency decreased their milk production. - Several women also reported introducing formula during maternity leave in lieu of breastfeeding so the child would sleep longer (so the mother could work from home) and would get used to eating formula in case the expressed milk ran out. - Breastfeeding breaks and flexible hours were reported to reduce maternal stress and enable breastfeeding during the workday - something that most institutions lack. |

1. Aguree S, Ziem JB, Issah A, Akrugu T, Joseph A. Maternal employment, child’s caring practices and nutritional status in Northern Ghana. *Int J Child Health Nutr*. 2015;4(1):54-60.

2. Chekol DA, Biks GA, Gelaw YA, Melsew YA. Exclusive breastfeeding and mothers’ employment status in Gondar town, Northwest Ethiopia: a comparative cross-sectional study. *Int Breastfeed J*. 2017;12:27. doi:10.1186/s13006-017-0118-9

3. Desalew A, Sema A, Belay Y. Exclusive Breastfeeding Practice and its Associated Factors Among Mothers with Children Aged 6-23 Months in Dire Dawa, Eastern Ethiopia: A Community-based Cross-sectional Study. *J Midwifery Reprod Health*. 2020;8(4):2419-2428. doi:10.22038/jmrh.2020.44011.1524

4. Emmanuel A, Clow SE. Does maternal employment affect breastfeeding in Plateau State, Nigeria? *Afr J Nurs Midwifery*. 2017;19(2). doi:10.25159/2520-5293/1130

5. Harrison GA, Brush G, Zumrawi FY. Motherhood and infant health in Khartoum. *Bull World Health Organ*. 1993;71(5):529-533.

6. Igbedioh SO. Influence of mother’s occupation and education on breast-feeding and weaning in infants and children in Markurdi, Nigeria. *Nutr Health*. 1993;9(4):289-302.

7. Kimani‐Murage EW, Wilunda C, Macharia TN, et al. Effect of a baby‐friendly workplace support intervention on exclusive breastfeeding in Kenya. *Matern Child Nutr*. 2021;17(4):e13191. doi:10.1111/mcn.13191

8. Shapiro D, Tambashe BO. Education, employment, and fertility in Kinshasa and prospects for changes in reproductive behavior. *Popul Res Policy Rev*. 1997;16(3):259-287. doi:10.1023/A:1005761504449

9. Ukwuani FA, Suchindran CM, Cornwell GT. Influences of mother’s work, childhood place of residence, and exposure to media on breast-feeding patterns: experience of Nigeria and Uganda. *Soc Biol*. 2001;48(1-2):1-20. doi:10.1080/19485565.2001.9989025

10. Adugna B, Tadele H, Reta F, Berhan Y. Determinants of exclusive breastfeeding in infants less than six months of age in Hawassa, an urban setting, Ethiopia. *Int Breastfeed J*. 2017;12:45. doi:10.1186/s13006-017-0137-6

11. Argaw MD, Asfaw MM, Ayalew MB, et al. Factors associated with prelacteal feeding practices in Debre Berhan district, North Shoa, Central Ethiopia: a cross-sectional, community-based study. *BMC Nutr*. 2019;5:14. doi:10.1186/s40795-019-0277-8

12. Asemahagn MA. Determinants of exclusive breastfeeding practices among mothers in azezo district, northwest Ethiopia. *Int Breastfeed J*. 2016;11:22. doi:10.1186/s13006-016-0081-x

13. Asfaw MM, Argaw MD, Kefene ZK. Factors associated with exclusive breastfeeding practices in Debre Berhan District, Central Ethiopia: a cross sectional community based study. *Int Breastfeed J*. 2015;10:23. doi:10.1186/s13006-015-0049-2

14. Ayele AA, Seid KA, Muhammed OS. Determinants of none-exclusive breast feeding practice among HIV positive women at selected Health Institutions in Ethiopia: case control study. *BMC Res Notes*. 2019;12(1):400. doi:10.1186/s13104-019-4457-z

15. Basnet S, Frongillo EA, Nguyen PH, Moore S, Arabi M. Associations of maternal resources with care behaviours differ by resource and behaviour. *Matern Child Nutr*. Published online 2020:e12977. doi:10.1111/mcn.12977

16. Berde AS. Factors Associated with Bottle Feeding in Namibia: Findings from Namibia 2013 Demographic and Health Survey. *J Trop Pediatr*. 2018;64(6):460-467. doi:10.1093/tropej/fmx091

17. Diji AK, Bam V, Asante E, Lomotey AY, Yeboah S, Owusu HA. Challenges and predictors of exclusive breastfeeding among mothers attending the child welfare clinic at a regional hospital in Ghana: a descriptive cross-sectional study. *Int Breastfeed J*. 2016;12:13. doi:10.1186/s13006-017-0104-2

18. Gara CP, Pazvakavambwa I, Maponga CC, Gavaza P. An investigation of the factors influencing the choice of infant feeding methods among urban Zimbabwean women in the context of HIV transmission. *Cent Afr J Med*. 2005;51(1-2):1-4.

19. Horii N, Allman J, Martin-Prével Y, Waltisperger D. Determinants of early initiation of breastfeeding in rural Niger: cross-sectional study of community based child healthcare promotion. *Int Breastfeed J*. 2017;12:41. doi:10.1186/s13006-017-0134-9

20. Hussein TH, Mgongo M, Uriyo JG, et al. Exclusive Breastfeeding Rates and Factors Associated with Exclusive Breastfeeding Practices in Northern Tanzania: Measurement using Two Different Methodologies-24 Hours Recall and Recall Since Birth. *Int J MCH AIDS*. 2019;8(1):32-43. doi:10.21106/ijma.258

21. Lawani LO, Onyebuchi AK, Iyoke CA, Onoh RC, Nkwo PO. The challenges of adherence to infant feeding choices in prevention of mother-to-child transmission of HIV infections in South East Nigeria. *Patient Prefer Adherence*. 2014;8:377-381. doi:10.2147/ppa.S61796

22. Mensah KA, Acheampong E, Anokye FO, Okyere P, Appiah-Brempong E, Adjei RO. Factors influencing the practice of exclusive breastfeeding among nursing mothers in a peri-urban district of Ghana. *BMC Res Notes*. 2017;10(1):466. doi:10.1186/s13104-017-2774-7

23. Napyo A, Tumwine JK, Mukunya D, Waako P, Tylleskär T, Ndeezi G. Exclusive breastfeeding among HIV exposed infants from birth to 14 weeks of life in Lira, Northern Uganda: a prospective cohort study. *Glob Health Action*. 2020;13(1):1833510. doi:10.1080/16549716.2020.1833510

24. Nieuwoudt S, Manderson L, Norris SA. Infant feeding practices in Soweto, South Africa: Implications for healthcare providers. *S Afr Med J*. 2018;108(9):756-762. doi:10.7196/SAMJ.2018.v108i9.13358

25. Nyanga NM, Musita C, Otieno A, Kaseje D. Factors influencing knowledge and practice of exclusive breastfeeding in Nyando District, Kenya. *Afr J Food Agric Nutr Dev*. 2012;12(6):6632-6645.

26. Ogwu A, Moyo S, Powis K, et al. Predictors of early breastfeeding cessation among HIV-infected women in Botswana. *Trop Med Int Health*. 2016;21(8):1013-1018. doi:10.1111/tmi.12729

27. Pascale KNA, Laure NJ, Enyong OJ. Factors associated with breast feeding as well as the nutritional status of infants (0-12) months: An epidemiological study in Yaounde, Cameroon. *Pak J Nutr*. 2007;6(3):259-263. doi:10.3923/pjn.2007.259.263

28. Qureshi AM, Oche OM, Sadiq UA, Kabiru S. Using community volunteers to promote exclusive breastfeeding in Sokoto State, Nigeria. *Pan Afr Med J*. 2011;10:8. doi:10.4314/pamj.v10i0.72215

29. Tewabe T, Mandesh A, Gualu T, Alem G, Mekuria G, Zeleke H. Exclusive breastfeeding practice and associated factors among mothers in Motta town, East Gojjam zone, Amhara Regional State, Ethiopia, 2015: A cross-sectional study. *Int Breastfeed J*. 2017;12(1). doi:10.1186/s13006-017-0103-3

30. Thomas E, Kuo C, Cohen S, et al. Mental health predictors of breastfeeding initiation and continuation among HIV infected and uninfected women in a South African birth cohort study. *Prev Med*. 2017;102:100-111. doi:10.1016/j.ypmed.2017.07.004

31. Toma Y, Emebet B, Manaye Y, Yinager W. Timely initiation of complementary feeding and associated factors among children aged 6 to 12 months in Addis Ababa Ethiopia, 2015. *Epidemiol Open Access*. 2016;6(5):272-272.

32. Yeboah JY, Forkuor D, Agyemang-Duah W. Exclusive breastfeeding practices and associated factors among lactating mothers of infants aged 6-24 months in the Kumasi Metropolis, Ghana. *BMC Res Notes*. 2019;12(1):689. doi:10.1186/s13104-019-4723-0

33. Adeniyi OV, Ajayi AI, Issah M, et al. Beyond health care providers’ recommendations: understanding influences on infant feeding choices of women with HIV in the Eastern Cape, South Africa. *Int Breastfeed J*. 2019;14:7. doi:10.1186/s13006-019-0201-5

34. Alabi TA, Adejoh SO, Atinge S, Umahi E. Social and Bio-Medical Predictors of Exclusive Breastfeeding Among Nursing Mothers in Lagos and Taraba States, Nigeria. *J Pediatr Nurs*. 2020;52:e96-e102. doi:10.1016/j.pedn.2019.12.002

35. Andare N, Ochola S, Chege P. Determinants of infant feeding practices among mothers living with HIV attending prevention of mother to child transmission Clinic at Kiambu Level 4 hospital, Kenya: a cross-sectional study. *Nutr J*. 2019;18(1):64. doi:10.1186/s12937-019-0490-y

36. Habte MH, Seid SJ, Alemu A, et al. The effect of unemployment and post-natal care on the exclusive breast-feeding practice of women in Ethiopia: a systematic review and meta-analysis. *Reprod Health*. 2022;19(1):94. doi:10.1186/s12978-022-01404-y

37. Jama NA, Wilford A, Masango Z, et al. Enablers and barriers to success among mothers planning to exclusively breastfeed for six months: a qualitative prospective cohort study in KwaZulu-Natal, South Africa. *Int Breastfeed J*. 2017;12:43. doi:10.1186/s13006-017-0135-8

38. Kiarie JN, Richardson BA, Mbori-Ngacha D, Nduati RW, John-Stewart GC. Infant feeding practices of women in a perinatal HIV-1 prevention study in Nairobi, Kenya. *J Acquir Immune Defic Syndr*. 2004;35(1):75-81. doi:10.1097/00126334-200401010-00011

39. Nieuwoudt SJ, Ngandu CB, Manderson L, Norris SA. Exclusive breastfeeding policy, practice and influences in South Africa, 1980 to 2018: A mixed-methods systematic review. *PloS One*. 2019;14(10):e0224029. doi:10.1371/journal.pone.0224029

40. Trafford Z, Jewett S, Swartz A, et al. Reported infant feeding practices and contextual influences on breastfeeding: qualitative interviews with women registered to MomConnect in three South African provinces. *Int Breastfeed J*. 2020;15(1):81. doi:10.1186/s13006-020-00315-7

41. Cuinhane CE, Coene G, Roelens K, Vanroelen C. Exploring perceptions and practices of biomedical norms during exclusive breastfeeding among HIV-positive lactating mothers in Mozambique. *J AIDS Clin Res*. 2017;8(4):687-687.

42. Goon DT, Ajayi AI, Adeniyi OV. Reasons for the Early Introduction of Complementary Feeding to HIV-Exposed Infants in the Eastern Cape, South Africa: An Exploratory Qualitative Study. *Med Kaunas Lith*. 2020;56(12). doi:10.3390/medicina56120703

43. Kimani‐Murage EW, Wekesah F, Wanjohi M, et al. Factors affecting actualisation of the WHO breastfeeding recommendations in urban poor settings in Kenya. *Matern Child Nutr*. 2015;11(3):314-332. doi:10.1111/mcn.12161

44. Matare CR, Craig HC, Martin SL, et al. Barriers and Opportunities for Improved Exclusive Breast-Feeding Practices in Tanzania: Household Trials With Mothers and Fathers. *Food Nutr Bull*. 2019;40(3):308-325. doi:10.1177/0379572119841961

45. Otoo GE, Lartey AA, Pérez-Escamilla R. Perceived incentives and barriers to exclusive breastfeeding among periurban Ghanaian women. *J Hum Lact*. 2009;25(1):34-41. doi:10.1177/0890334408325072

46. Talbert AW, Tsofa B, Mumbo E, Berkley JA, Mwangome M. Knowledge of, and attitudes to giving expressed breastmilk to infants in rural coastal Kenya; focus group discussions of first time mothers and their advisers. *Int Breastfeed J*. 2018;13:16. doi:10.1186/s13006-018-0158-9

47. Tampah-Naah AM, Kumi-Kyereme A, Amo-Adjei J. Maternal challenges of exclusive breastfeeding and complementary feeding in Ghana. *PloS One*. 2019;14(5):e0215285. doi:10.1371/journal.pone.0215285

48. van der Merwe P, Mojapelo-Batka M. Women’s breast-feeding meanings in the context of mother-to-child transmission of HIV. *J Psychol Afr*. 2018;28(3):229-232. doi:10.1080/14330237.2018.1475463

49. Ickes SB, Oddo VM, Sanders HK, et al. Formal maternal employment is associated with lower odds of exclusive breastfeeding by 14 weeks postpartum: a cross-sectional survey in Naivasha, Kenya. *Am J Clin Nutr*. 2021;113(3):562-573. doi:10.1093/ajcn/nqaa351

50. Nkrumah J. Maternal work and exclusive breastfeeding practice: A community based cross-sectional study in Efutu Municipal, Ghana. *Int Breastfeed J*. 2017;12(1). doi:10.1186/s13006-017-0100-6

51. Ahmed KY, Page A, Arora A, Ogbo FA. Trends and determinants of early initiation of breastfeeding and exclusive breastfeeding in Ethiopia from 2000 to 2016. *Int Breastfeed J*. 2019;14:40. doi:10.1186/s13006-019-0234-9

52. Asare BY, Preko JV, Baafi D, Dwumfour-Asare B. Breastfeeding practices and determinants of exclusive breastfeeding in a cross-sectional study at a child welfare clinic in Tema Manhean, Ghana. *Int Breastfeed J*. 2018;13:12. doi:10.1186/s13006-018-0156-y

53. Dalcastagnê SV, Giugliani ERJ, Nunes LN, Hauser L, Giugliani C. Practice of exclusive breastfeeding and its associated factors in a suburban area in Angola: a cross-sectional study. *Sao Paulo Med J*. 2018;136(6):533-542. doi:10.1590/1516-3180.2018.0262161118

54. Lakati A, Binns C, Stevenson M. The effect of work status on exclusive breastfeeding in Nairobi. *Asia Pac J Public Health*. 2002;14(2):85-90. doi:10.1177/101053950201400206

55. Ogunlesi TA. Maternal socio-demographic factors influencing the initiation and exclusivity of breastfeeding in a Nigerian semi-urban setting. *Matern Child Health J*. 2010;14(3):459-465. doi:10.1007/s10995-008-0440-3

56. Yako EM, Nzama NPB. Maintenance of the selected infant feeding methods amongst postnatal mothers at risk of HIV in the Eastern Cape Province, South Africa. *Health SA Gesondheid*. 2013;18(1). doi:10.4102/hsag.v18i1.585

57. Safari JG, Kimambo SC, Lwelamira JE. Feeding practices and nutritional status of infants in Morogoro Municipality, Tanzania. *Tanzan J Health Res*. 2013;15(3):178-185. doi:10.4314/thrb.v15i3.5

58. Stumbitz B, Lewis S, Kyei AA, Lyon F. Maternity protection in formal and informal economy workplaces: The case of Ghana. *World Dev*. 2018;110:373-384. doi:10.1016/j.worlddev.2018.06.007

59. Mgongo M, Hussein TH, Stray-Pedersen B, Vangen S, Msuya SE, Wandel M. “We give water or porridge, but we don’t really know what the child wants:” a qualitative study on women’s perceptions and practises regarding exclusive breastfeeding in Kilimanjaro region, Tanzania. *BMC Pregnancy Childbirth*. 2018;18(1):323. doi:10.1186/s12884-018-1962-3

60. Adeyinka T, Ajibola F, Oyesoji A, Adedeji T. A hospital-based assessment of breast-feeding behaviour and practices among nursing mothers in Nigeria and Ghana. *Pak J Nutr*. 2008;7(1):165-171. doi:10.3923/pjn.2008.165.171

61. Ekanem IA, Ekanem AP, Asuquo A, Eyo VO. Attitude of working mothers to exclusive breastfeeding in Calabar Municipality, Cross River State, Nigeria. *J Food Res*. 2012;1(2):71-75.

62. Hunegnaw MT, Gezie LD, Teferra AS. Exclusive breastfeeding and associated factors among mothers in Gozamin district, northwest Ethiopia: a community based cross-sectional study. *Int Breastfeed J*. 2017;12:30. doi:10.1186/s13006-017-0121-1

63. Igbedioh SO. Influence of mother’s occupation and education on breast-feeding and weaning in infants and children in Makurdi, Nigeria. *Nutr Health*. 1994;9(4):289-302. doi:10.1177/026010609400900405

64. Adewuyi EO, Zhao Y, Khanal V, Auta A, Bulndi LB. Rural-urban differences on the rates and factors associated with early initiation of breastfeeding in Nigeria: further analysis of the Nigeria demographic and health survey, 2013. *Int Breastfeed J*. 2017;12:51. doi:10.1186/s13006-017-0141-x

65. Aghaji MN. Exclusive breast-feeding practice and associated factors in Enugu, Nigeria. *West Afr J Med*. 2002;21(1):66-69.

66. Alemu A, Eshete A. Newborn Care Practices and Associated Factors Among Lactating Mothers at Home in the Rural Districts of Gedeo Zone, Southern Ethiopia. *Pediatr Health Med Ther*. 2020;11:47-54. doi:10.2147/phmt.S232860

67. Asekun-Olarinmoye EO, Lawoyin TO, Asekun-Olarinmoye IO. Effect of rearing environment on the feeding pattern of under two years old Nigerian children. *Early Child Dev Care*. 2011;181(10):1331-1342. doi:10.1080/03004430.2010.526209

68. Awoke N, Tekalign T, Lemma T. Predictors of optimal breastfeeding practices in Worabe town, Silte zone, South Ethiopia. *PloS One*. 2020;15(4):e0232316. doi:10.1371/journal.pone.0232316

69. Ayawine A, Ae-Ngibise KA. Determinants of exclusive breastfeeding: a study of two sub-districts in the Atwima Nwabiagya District of Ghana. *Pan Afr Med J*. 2015;22:248. doi:10.11604/pamj.2015.22.248.6904

70. Bayissa ZB. Exclusive breast feeding status and its determinant among HIV positive women in West showa zone Oromia region Ethiopia. *J AIDS Clin Res*. 2017;8(1):646-646.

71. Berde AS, Yalcin SS. Determinants of early initiation of breastfeeding in Nigeria: a population-based study using the 2013 demograhic and health survey data. *BMC Pregnancy Childbirth*. 2016;16:32. doi:10.1186/s12884-016-0818-y

72. Berhe M, Medhaniye AA, Kahsay G, Birhane E, Abay M. Essential neonatal care utilization and associated factors among mothers in public health facilities of Aksum Town, North Ethiopia, 2016. *PloS One*. 2017;12(4):e0175902. doi:10.1371/journal.pone.0175902

73. Bodjrènou FSU, Amoussa Hounkpatin W, Termote C, Dato G, Savy M. Determining factors associated with breastfeeding and complementary feeding practices in rural Southern Benin. *Food Sci Nutr*. 2021;9(1):135-144. doi:10.1002/fsn3.1971

74. Chineke HN, Iwu AC, Diwe KC, et al. The practice of exclusive breastfeeding and its sociodemographic determinants amongst nursing mothers at a tertiary health care institution in South East, Nigeria. *Open J Prev Med*. 2017;7(4):63-73.

75. Chuwa M, Mgaya BB. FACTORS HINDERING BREASTFEEDING PRACTICES AMONG MOTHERS IN RURAL TANZANIA. *Afr J Midwifery Womens Health*. 2013;7(2):91-95. doi:10.12968/ajmw.2013.7.2.91

76. Dare AA, Joseph H, Femi O. Socio-Demographic Correlates of Breastfeedling Practices Among Mothers in Kogi State, Nigeria. *West Afr J Nurs*. 2011;22(1):28-37.

77. Davies-Adetugbo AA, Ojofeitimi EO. Maternal education, breastfeeding behaviours and lactational amenorrhoea: studies among two ethnic communities in Ile Ife, Nigeria. *Nutr Health*. 1996;11(2):115-126. doi:10.1177/026010609601100204

78. Eskezyiaw A, Meaza D, Direslgne M, Desta H. Early initiation of complementary feeding and associated factors among 6 months to 2 years young children, in Kamba Woreda, South West Ethiopia: a community-based cross-sectional study. *J Nutr Food Sci*. 2014;4(6):314-314.

79. Gebremedhin T, Geberu DM, Atnafu A. Less than one-fifth of the mothers practised exclusive breastfeeding in the emerging regions of Ethiopia: a multilevel analysis of the 2016 Ethiopian demographic and health survey. *BMC Public Health*. 2021;21(1):18. doi:10.1186/s12889-020-10071-2

80. Hailu WS, Bayih MT, Babble NF. Four in every ten infants in Northwest Ethiopia exposed to sub-optimal breastfeeding practice. *PloS One*. 2020;15(11):e0238576. doi:10.1371/journal.pone.0238576

81. Igbedioh SO, Edache A, Kaka HJ. Infant weaning practises of some Idoma women in Makurdi, Nigeria. *Nutr Health*. 1995;10(3):239-253. doi:10.1177/026010609501000308

82. Igbedioh SO, Aderiye JBI. Breastfeeding pattern and weaning practices in infants and children in makurdi, nigeria under changing socio-economic condition. *Ecol Food Nutr*. 1992;29(1):45-60. doi:10.1080/03670244.1992.9991290

83. Kebebe T, Assaye H. Intention, magnitude and factors associated with bottle feeding among mothers of 0-23 months old children in Holeta town, Central Ethiopia: a cross sectional study. *BMC Nutr*. 2017;3:53. doi:10.1186/s40795-017-0174-y

84. Kenechi OS, Chidiebere ODI, Joy E, Clement E, Uchenna E, Ifeyinwa N. Infant feeding practices and growth pattern in the first six months of life: a cross-sectional study of babies attending the infant welfare clinic of the Nnamdi Azikiwe University Teaching Hospital. *Br J Med Med Res*. 2015;6(9):935-947.

85. Komakech H, Lubogo D, Nabiwemba E, Orach CG. Essential newborn care practices and determinants amongst mothers of infants aged 0-6 months in refugee settlements, Adjumani district, west Nile, Uganda. *PloS One*. 2020;15(4):e0231970. doi:10.1371/journal.pone.0231970

86. Liben ML, Gemechu YB, Adugnew M, et al. Factors associated with exclusive breastfeeding practices among mothers in dubti town, afar regional state, northeast Ethiopia: a community based cross-sectional study. *Int Breastfeed J*. 2016;11:4. doi:10.1186/s13006-016-0064-y

87. Mahgoub SE, Bandeke T, Nnyepi M. Breastfeeding in Botswana: practices, attitudes, patterns, and the socio-cultural factors affecting them. *J Trop Pediatr*. 2002;48(4):195-199. doi:10.1093/tropej/48.4.195

88. Manyeh AK, Amu A, Akpakli DE, Williams JE, Gyapong M. Estimating the rate and determinants of exclusive breastfeeding practices among rural mothers in Southern Ghana. *Int Breastfeed J*. 2020;15(1):7. doi:10.1186/s13006-020-0253-6

89. Muluye D, Woldeyohannes D, Gizachew M, Tiruneh M. Infant feeding practice and associated factors of HIV positive mothers attending prevention of mother to child transmission and antiretroviral therapy clinics in Gondar Town health institutions, Northwest Ethiopia. *BMC Public Health*. 2012;12:240. doi:10.1186/1471-2458-12-240

90. Ndiokwelu CI, Maduforo AN, Amadi CA, Okwy-Nweke CP. Breastfeeding and complementary feeding practices of mothers of children (0-24 months) attending infant welfare clinice (IWC) at the Institute of Child Health (ICH) University of Nigerian Teaching Hospital (UNTH) Ituku-Ozalla Enugu. *J Biol Agric Healthc*. 2014;4(11):5-15.

91. Ohaeri BM, Bello SS. Exploring the barriers to exclusive breastfeeding in Ibadan North Local Government Area, Oyo State, Nigeria. *Afr J Midwifery Womens Health*. 2016;10(4):162-167.

92. Okeh UM. Breastfeeding and the mother-child relationship: A case study of Ebonyi State University Teaching Hospital, Abakaliki. *Afr J Prim Health Care Fam Med*. 2010;2(1). doi:10.4102/phcfm.v2i1.97

93. Reda EB, Teferra AS, Gebregziabher MG. Time to initiate complementary feeding and associated factors among mothers with children aged 6-24 months in Tahtay Maichew district, northern Ethiopia. *BMC Res Notes*. 2019;12(1):17. doi:10.1186/s13104-019-4061-2

94. Saaka M, Takyi SA, Maxwell T. An investigation of patterns and factors associated with exclusive breast feeding in Northern Ghana. *Int J Child Health Nutr*. 2012;1(2):92-103.

95. Sonko A, Worku A. Prevalence and predictors of exclusive breastfeeding for the first six months of life among women in Halaba special woreda, Southern Nations, Nationalities and Peoples’ Region/SNNPR/, Ethiopia: a community based cross-sectional study. *Arch Public Health*. 2015;73:53. doi:10.1186/s13690-015-0098-4

96. Warille EB, Onyango FE, Osano B. Knowledge and practice of exclusive breastfeeding among women with children aged between 9 and 12 months in Al-Sabah Children Hospital, Juba, South Sudan. *South Sudan Med J*. 2017;10(1):12-16.

97. W’Gebriel A. Determinants of weaning practices. *Ethiop J Health Dev*. 2000;14(2):183-189.

98. Zenebu Begna B, Belayneh Kefale G, Alayou G, et al. Knowledge and practice of mothers towards exclusive breastfeeding and its associated factors in Ambo Woreda West Shoa Zone Oromia Region, Ethiopia. *Epidemiol Open Access*. 2015;5(1):182-182.

99. Abebe B, Wondu G, Fekadu B. Exclusive breastfeeding practices of HIV positive mothers and its determinants in selected health institution of West Oromia, Ethiopia. *J Nutr Food Sci*. 2014;4(6):319-319.

100. Agunbiade OM, Ogunleye OV. Constraints to exclusive breastfeeding practice among breastfeeding mothers in Southwest Nigeria: implications for scaling up. *Int Breastfeed J*. 2012;7:5. doi:10.1186/1746-4358-7-5

101. Burns J, Emerson JA, Amundson K, Doocy S, Caulfield LE, Klemm RD. A Qualitative Analysis of Barriers and Facilitators to Optimal Breastfeeding and Complementary Feeding Practices in South Kivu, Democratic Republic of Congo. *Food Nutr Bull*. 2016;37(2):119-131. doi:10.1177/0379572116637947

102. Demelew MZ, Abdeta G. Assessment of exclusive breastfeeding practice among HIV positive women in Addis Ababa. *Afr J Midwifery Womens Health*. 2014;8(1):14-20. doi:10.12968/ajmw.2014.8.1.14

103. Melese Ayele W. Exclusive Breastfeeding and Normative Belief among Rural Mothers in Ethiopia, 2019: A Cross-Sectional Survey Embedded with Qualitative Design. *Obstet Gynecol Int*. 2021;2021:5587790. doi:10.1155/2021/5587790

104. Muko KN, Tchangwe GK, Ngwa VC, Njoya L. Preventing mother-to-child transmission: factors affecting mothers’ choice of feeding--a case study from Cameroon. *SAHARA J*. 2004;1(3):132-138. doi:10.1080/17290376.2004.9724836

105. Tebikew Y, Tefera B, Muluneh H. Determinants of cessation of exclusive breastfeeding in Ankesha Guagusa Woreda, Awi Zone, Northwest Ethiopia: a cross-sectional study. *BMC Pregnancy Childbirth*. 2014;14(262):(9 August 2014)-(9 August 2014).

106. Derose LF. Women’s work and breastfeeding simultaneously rise in Ghana. *Econ Dev Cult Change*. 2007;55(3):583-612. doi:10.1086/511193

107. Mazengia AL, Demissie H. Knowledge and Practice of Employed Mothers towards Exclusive Breastfeeding and Its Associated Factors in Mecha District, Northwest Ethiopia. *J Nutr Metab*. 2020;2020. doi:10.1155/2020/4820582

108. Ogunba BO. Effect of maternal employment on infant feeding practices in Southwestern Nigeria. *Food Nutr Sci*. 2015;6(7):597-604.

109. Balogun MR, Okpalugo OA, Ogunyemi AO, Sekoni AO. Knowledge, Attitude, and Practice of Breastfeeding: A Comparative Study of Mothers in Urban and Rural Communities of Lagos, Southwest Nigeria. *Niger Med J*. 2017;58(4):123-130. doi:10.4103/nmj.NMJ_289_16

110. Lawoyin TO, Olawuyi JF, Onadeko MO. Factors associated with exclusive breastfeeding in Ibadan, Nigeria. *J Hum Lact*. 2001;17(4):321-325. doi:10.1177/089033440101700406

111. Matanda DJ, Mittelmark MB, Urke HB, Amugsi DA. Reliability of demographic and socioeconomic variables in predicting early initiation of breastfeeding: a replication analysis using the Kenya Demographic and Health Survey data. *BMJ Open*. 2014;4(6):e005194. doi:10.1136/bmjopen-2014-005194

112. Motee A, Ramasawmy D, Pugo-Gunsam P, Jeewon R. An Assessment of the Breastfeeding Practices and Infant Feeding Pattern among Mothers in Mauritius. *J Nutr Metab*. 2013;2013:243852. doi:10.1155/2013/243852

113. Nwankwo BO, Brieger WR. Exclusive breastfeeding is undermined by use of other liquids in rural southwestern Nigeria. *J Trop Pediatr*. 2002;48(2):109-112. doi:10.1093/tropej/48.2.109

114. Anyanwu OU, Ezeonu CT, Ezeanosike OB, Okike CO. Breastfeeding practices as observed in those attending a teaching hospital for perinatal care. *J Nepal Paediatr Soc*. 2014;34(2):90-95. doi:10.3126/jnps.v34i2.8788

115. Ghuman MR, Saloojee H, Morris G. Infant feeding practices in a high HIV prevalence rural district of KwaZulu-Natal, South Africa. *South Afr J Clin Nutr*. 2009;22(2):74-79. doi:10.1080/16070658.2009.11734222

116. Horwood C, Surie A, Haskins L, et al. Attitudes and perceptions about breastfeeding among female and male informal workers in India and South Africa. *BMC Public Health*. 2020;20(1):875. doi:10.1186/s12889-020-09013-9

117. Mbawalla HS, Majid S. Infant feeding practices with oral health implications among suburban mothers of Tanzania. *Int J Child Health Nutr*. 2017;6(4):159-165.

118. Mekuria G, Edris M. Exclusive breastfeeding and associated factors among mothers in Debre Markos, Northwest Ethiopia: a cross-sectional study. *Int Breastfeed J*. 2015;10(1):1. doi:10.1186/s13006-014-0027-0

119. Olaitan IN, Onimawo IA, Nkwoala CC. Characteristics and micronutrient intakes of exclusively and non-exclusively breastfeeding mothers in Imo state of Nigeria. *Int J Food Sci Nutr Eng*. 2015;5(1):68-73.

120. Siziba LP, Jerling J, Hanekom SM, Wentzel-Viljoen E. Low rates of exclusive breastfeeding are still evident in four South African provinces. *South Afr J Clin Nutr*. 2015;28(4):170-179. doi:10.1080/16070658.2015.11734557

121. Sobo RA, Sokoya GO, Awonusi PA, Odufuwa BA. Knowledge attitudes and practice of exclusive breastfeeding among rural mothers in Ijebu-Ode, Ogun State, Nigeria. *West Afr J Nurs*. 2008;19(2):121-124.

122. Coetzee B, Tomlinson M, Osawe S, Abimiku A, Kagee A. Barriers to and Facilitators of Adherence to Exclusive Breastfeeding Practices Among HIV Infected and Non-Infected Women in Jos, Nigeria. *Matern Child Health J*. 2017;21(4):953-960. doi:10.1007/s10995-016-2253-0

123. Doherty T, Horwood C, Haskins L, et al. Breastfeeding advice for reality: Women’s perspectives on primary care support in South Africa. *Matern Child Nutr*. 2020;16(1):e12877. doi:10.1111/mcn.12877

124. Hunter-Adams J, Myer L, Rother HA. Perceptions related to breastfeeding and the early introduction of complementary foods amongst migrants in Cape Town, South Africa. *Int Breastfeed J*. 2016;11:29. doi:10.1186/s13006-016-0088-3

125. Omer-Salim A, Persson LA, Olsson P. Whom can I rely on? Mothers’ approaches to support for feeding: an interview study in suburban Dar es Salaam, Tanzania. *Midwifery*. 2007;23(2):172-183. doi:10.1016/j.midw.2006.05.002

126. Samburu BM, Kimiywe J, Young SL, et al. Realities and challenges of breastfeeding policy in the context of HIV: a qualitative study on community perspectives on facilitators and barriers related to breastfeeding among HIV positive mothers in Baringo County, Kenya. *Int Breastfeed J*. 2021;16(1):39. doi:10.1186/s13006-021-00385-1

127. Wainaina CW, Wanjohi M, Wekesah F, Woolhead G, Kimani-Murage E. Exploring the Experiences of Middle Income Mothers in Practicing Exclusive Breastfeeding in Nairobi, Kenya. *Matern Child Health J*. 2018;22(4):608-616. doi:10.1007/s10995-018-2430-4

128. Kumeh OW, Fallah MP, Desai IK, et al. Literacy is power: structural drivers of child malnutrition in rural Liberia. *BMJ Nutr Prev Health*. 2020;3(2):295-307. doi:10.1136/bmjnph-2020-000140

129. Moshy VH, Masenge TJ, Bryceson I. Undernutrition among under-five children in two fishing communities in Mafia Island Marine Park, Tanzania. *J Sustain Dev*. 2013;6(6):1-14.

130. Mushaphi LF, Mahopo TC, Nesamvuni CN, et al. Recommendations for Infant Feeding Policy and Programs in Dzimauli Region, South Africa: Results From the MAL-ED Birth Cohort. *Food Nutr Bull*. 2017;38(3):428-440. doi:10.1177/0379572117696662

131. West NS, Schwartz SR, Yende N, et al. Infant feeding by South African mothers living with HIV: implications for future training of health care workers and the need for consistent counseling. *Int Breastfeed J*. 2019;14:11. doi:10.1186/s13006-019-0205-1

132. Zulliger R, Abrams EJ, Myer L. Diversity of influences on infant feeding strategies in women living with HIV in Cape Town, South Africa: a mixed methods study. *Trop Med Int Health*. 2013;18(12):1547-1554. doi:10.1111/tmi.12212

133. Horwood C, Haskins L, Alfers L, Masango-Muzindutsi Z, Dobson R, Rollins N. A descriptive study to explore working conditions and childcare practices among informal women workers in KwaZulu-Natal, South Africa: identifying opportunities to support childcare for mothers in informal work. *BMC Pediatr*. 2019;19(1):382. doi:10.1186/s12887-019-1737-7

134. Nabunya P, Mubeezi R, Awor P. Prevalence of exclusive breastfeeding among mothers in the informal sector, Kampala Uganda. *PloS One*. 2020;15(9):e0239062. doi:10.1371/journal.pone.0239062

135. Luthuli S, Haskins L, Mapumulo S, Rollins N, Horwood C. “I decided to go back to work so I can afford to buy her formula”: a longitudinal mixed-methods study to explore how women in informal work balance the competing demands of infant feeding and working to provide for their family. *BMC Public Health*. 2020;20(1):1847. doi:10.1186/s12889-020-09917-6

136. Mulindwa IN, Ntozi JPM. Mothers in the informal economy and changes in child feeding and caring roles in Kampala, Uganda. *Afr Dev Afr Dev*. 2004;29(3):114-130.

137. Berihun Assefa D, Berhanu Boru B. Breastfeeding practice and associated factors among female nurses and midwives at North Gondar Zone, Northwest Ethiopia: a cross-sectional institution based study. *Int Breastfeed J*. 2014;9(11):(21 July 2014)-(21 July 2014).

138. Dun-Dery EJ, Laar AK. Exclusive breastfeeding among city-dwelling professional working mothers in Ghana. *Int Breastfeed J*. 2016;11(1):23. doi:10.1186/s13006-016-0083-8

139. Tesfa Dejenie H, Mohammed SH, Aklilu E, et al. Breast and complementary feeding in Ethiopia: new national evidence from systematic review and meta-analyses of studies in the past 10 years. *Eur J Nutr*. 2019;58(7):2565-2595.

140. Abekah-Nkrumah G, Antwi MY, Nkrumah J, Gbagbo FY. Examining working mothers’ experience of exclusive breastfeeding in Ghana. *Int Breastfeed J*. 2020;15(1):56. doi:10.1186/s13006-020-00300-0

141. Gebrekidan K, Plummer V, Fooladi E, Hall H. Attitudes and experiences of employed women when combining exclusive breastfeeding and work: A qualitative study among office workers in Northern Ethiopia. *Matern Child Nutr*. Published online 2021:e13190. doi:10.1111/mcn.13190

142. Ickes SB, Sanders H, Denno DM, et al. Exclusive breastfeeding among working mothers in Kenya: Perspectives from women, families and employers. *Matern Child Nutr*. Published online 2021:e13194. doi:10.1111/mcn.13194

143. Mabaso BP, Jaga A, Doherty T. Experiences of workplace breastfeeding in a provincial government setting: a qualitative exploratory study among managers and mothers in South Africa. *Int Breastfeed J*. 2020;15(1):100. doi:10.1186/s13006-020-00342-4

144. Mlay RS, Keddy B, Stern PN. Demands out of context: Tanzanian women combining exclusive breastfeeding with employment. *Health Care Women Int*. 2004;25(3):242-254. doi:10.1080/07399330490272741

145. Stumbitz B, Jaga A. A Southern encounter: Maternal body work and low-income mothers in South Africa. *Gend Work Organ*. 2020;27(6):1485-1500. doi:10.1111/gwao.12527

146. Iliyasu Z, Galadanci HS, Emokpae P, Amole TG, Nass N, Aliyu MH. Predictors of exclusive breastfeeding among health care workers in urban Kano, Nigeria. *J Obstet Gynecol Neonatal Nurs*. 2019;48(4):433-444.

147. Kebede T, Woldemichael K, Jarso H, Bekele BB. Exclusive breastfeeding cessation and associated factors among employed mothers in Dukem town, Central Ethiopia. *Int Breastfeed J*. 2020;15(1):6. doi:10.1186/s13006-019-0250-9

148. Anyanwu OU, Ezeonu CT, Ezeanosike OB, Okike CO. The practice of breastfeeding by healthcare workers in the federal teaching hospital, Abakaliki, southeastern Nigeria. *SAJCH South Afr J Child Health*. 2014;8(2):55-58. doi:10.7196/SAJCH.668

149. Dachew BA, Bifftu BB. Breastfeeding practice and associated factors among female nurses and midwives at North Gondar Zone, Northwest Ethiopia: a cross-sectional institution based study. *Int Breastfeed J*. 2014;9(1):11. doi:10.1186/1746-4358-9-11

150. Habtewold TD, Mohammed SH, Endalamaw A, et al. Breast and complementary feeding in Ethiopia: new national evidence from systematic review and meta-analyses of studies in the past 10 years. *Eur J Nutr*. 2019;58(7):2565-2595. doi:10.1007/s00394-018-1817-8

151. Horwood C, Haskins L, Engebretsen I, Connolly C, Coutsoudis A, Spies L. Are we doing enough? Improved breastfeeding practices at 14 weeks but challenges of non-initiation and early cessation of breastfeeding remain: findings of two consecutive cross-sectional surveys in KwaZulu-Natal, South Africa. *BMC Public Health*. 2020;20(1):440. doi:10.1186/s12889-020-08567-y

152. Kimani-Murage EW, Wilunda C, Macharia TN, et al. Effect of a baby-friendly workplace support intervention on exclusive breastfeeding in Kenya. *Matern Child Nutr*. Published online 2021:e13191. doi:10.1111/mcn.13191

153. Lakati A, Binns C, Stevenson M. Breast-feeding and the working mother in Nairobi. *Public Health Nutr*. 2002;5(6):715-718. doi:10.1079/phn2002349

154. Mohammed A, Aliyu I. The knowledge, acceptance, and practice of exclusive breastfeeding among caregivers seen in a pediatric outpatient department and immunization clinic. *Sahel Med J*. 2021;24(1):48-54. doi:10.4103/smj.smj-28-20

155. Osibogun OO, Olufunlayo TF, Oyibo SO. Knowledge, attitude and support for exclusive breastfeeding among bankers in Mainland Local Government in Lagos State, Nigeria. *Int Breastfeed J*. 2018;13:38. doi:10.1186/s13006-018-0182-9

156. Sadoh AE, Sadoh WE, Oniyelu P. Breast Feeding Practice among Medical Women in Nigeria. *Niger Med J*. 2011;52(1):7-12.

157. Tsegaw SA, Ali Dawed Y, Tadesse Amsalu E. Exploring the determinants of exclusive breastfeeding among infants under-six months in Ethiopia using multilevel analysis. *PloS One*. 2021;16(1):e0245034. doi:10.1371/journal.pone.0245034

158. Tsegaw SA, Dawed YA, Amsalu ET. Individual level and community level factors affecting exclusive breast feeding among infants under-six months in Ethiopia using multilevel analysis. *Ital J Pediatr*. 2021;47(1):106. doi:10.1186/s13052-021-01062-z

159. Tadesse F, Alemayehu Y, Shine S, Asresahegn H, Tadesse T. Exclusive breastfeeding and maternal employment among mothers of infants from three to five months old in the Fafan zone, Somali regional state of Ethiopia: a comparative cross-sectional study. *BMC Public Health*. 2019;19(1):1015. doi:10.1186/s12889-019-7345-5

160. Goon DT, Ajayi AI, Adeniyi OV. Sociodemographic and lifestyle correlates of exclusive breastfeeding practices among mothers on antiretroviral therapy in the Eastern Cape, South Africa. *Int Breastfeed J*. 2021;16(1):18. doi:10.1186/s13006-021-00366-4

161. Sadoh AE, Sadoh WE, Oniyelu P. Breast Feeding Practice among Medical Women in Nigeria. *Niger Med J*. 2011;52(1):7-12.

162. Teshale AB, Worku MG, Tesema GA. Spatial distribution and determinants of the change in pre-lacteal feeding practice over time in Ethiopia: A spatial and multivariate decomposition analysis. *PloS One*. 2021;16(1):e0244574. doi:10.1371/journal.pone.0244574

163. Bekere A. Exclusive Breastfeeding Practices of HIV Positive mothers and its Determinants in Selected Health Institution of West Oromia, Ethiopia. *J Nutr Food Sci*. 2014;04(06). doi:10.4172/2155-9600.1000319

164. Ahinkorah BO, Seidu AA, Budu E, et al. Maternal and child factors associated with early initiation of breastfeeding in Chad: evidence from nationally representative cross-sectional data. *Int Health*. 2022;14(5):510-518. doi:10.1093/inthealth/ihab060

165. Appiah F, Ahinkorah BO, Budu E, et al. Maternal and child factors associated with timely initiation of breastfeeding in sub-Saharan Africa. *Int Breastfeed J*. 2021;16(1):55. doi:10.1186/s13006-021-00402-3

166. Armar-Klemesu M, Ruel MT, Maxwell DG, Levin CE, Morris SS. Poor maternal schooling is the main constraint to good child care practices in Accra. *J Nutr*. 2000;130(6):1597-1607. doi:10.1093/jn/130.6.1597

167. Bankole TO, Solanke BL, Bisiriyu LA. What Are the Individual, Household, and Community Factors Associated With Optimal Breastfeeding Practices in Three Selected West African Countries? *J Popul Soc Stud*. 2022;30:797-815. doi:10.25133/JPSSv302022.044

168. Bekele A, Berhane Y. Magnitude and determinants of bottle feeding in rural communities. *East Afr Med J*. 1999;76(9):516-519.

169. Cherop CE, Keverenge-Ettyang AG, Mbagaya GM. Barriers to exclusive breastfeeding among infants aged 0-6 months in Eldoret municipality, Kenya. *East Afr J Public Health*. 2009;6(1):69-72. doi:10.4314/eajph.v6i1.45752

170. WHO MULTICENTRE GROWTH REFERENCE STUDY GROUP, De Onis M. Breastfeeding in the WHO Multicentre Growth Reference Study. *Acta Paediatr*. 2006;95(S450):16-26. doi:10.1111/j.1651-2227.2006.tb02372.x

171. Doherty T, Sanders D, Jackson D, et al. Early cessation of breastfeeding amongst women in South Africa: an area needing urgent attention to improve child health. *BMC Pediatr*. 2012;12:105. doi:10.1186/1471-2431-12-105

172. Esan OT, Olajide FO, Olubosede OA, Adeyanju TA. Breastfeeding practices of physician-mothers in Ife and Ilesa zones, Osun State, Nigeria. *Afr J Med Med Sci*. 2013;42(4):293-299.

173. Feleke DG, Kassahun CW, G/Mariam W/mariam T, Tassaw SF, Chanie ES. Non-exclusive breast feeding and its factors in the first 6-month life of infants among mother-infant pairs of 6–12 months in Debre Tabor town, Northwest Ethiopia, 2019: community-based cross-sectional study. *Heliyon*. 2021;7(4). doi:10.1016/j.heliyon.2021.e06922

174. Horwood C, Haskins L, Engebretsen IM, et al. Improved rates of exclusive breastfeeding at 14 weeks of age in KwaZulu Natal, South Africa: what are the challenges now? *BMC Public Health*. 2018;18(1):757. doi:10.1186/s12889-018-5657-5

175. Issaka AI, Agho KE, Page AN, Burns P, Stevens GJ, Dibley MJ. Determinants of early introduction of solid, semi-solid or soft foods among infants aged 3-5 months in four Anglophone West African countries. *Nutrients*. 2014;6(7):2602-2618. doi:10.3390/nu6072602

176. Jahanpour OF, Okango EL, Todd J, Mwambi H, Mahande MJ. Role of clusters in exclusive breastfeeding practices in Tanzania: A secondary analysis study using demographic and health survey data (2015/2016). *Front Pediatr*. 2022;10:939706. doi:10.3389/fped.2022.939706

177. Jimoh AO, Adaji SE, Adelaiye HA, et al. Factors associated with prelacteal feeding practices in a rural Northern nigerian setting. *South Afr J Clin Nutr*. 2018;31(2):13-18. doi:10.1080/16070658.2017.1359391

178. Kaldenbach S, Engebretsen IMS, Haskins L, Conolly C, Horwood C. Infant feeding, growth monitoring and the double burden of malnutrition among children aged 6 months and their mothers in KwaZulu‐Natal, South Africa. *Matern Child Nutr*. 2022;18(1):1-9. doi:10.1111/mcn.13288

179. Kulwa KB, Kinabo JL, Modest B. Constraints on good child-care practices and nutritional status in urban Dar-es-Salaam, Tanzania. *Food Nutr Bull*. 2006;27(3):236-244. doi:10.1177/156482650602700306

180. Lakew Y, Tabar L, Haile D. Socio-medical determinants of timely breastfeeding initiation in Ethiopia: Evidence from the 2011 nation wide Demographic and Health Survey. *Int Breastfeed J*. 2015;10:24. doi:10.1186/s13006-015-0050-9

181. Morhason-Bello IO, Yusuf OB, Akinyemi JO, et al. Prevalence and predictive factors for early initiation of breastfeeding in Nigeria: Evidence from the Nigerian demographic and health survey (2003-2018). *Afr J Reprod Health*. 2022;26(11):28-43. doi:10.29063/ajrh2022/v26i11s.3

182. Ogbo FA, Page A, Agho KE, Claudio F. Determinants of trends in breast-feeding indicators in Nigeria, 1999-2013. *Public Health Nutr*. 2015;18(18):3287-3299. doi:10.1017/s136898001500052x

183. Okechukwu FO, Nnodim EJ, Ezeonyeche CL, Nnubia UI, Igbokwe CC, Ogbonnaya EK. Child-care services adopted by mothers in banks (With children birth-5years) in nsukka local government area, Enugu state. *J Home Econ Res*. 2020;27(1):58-66.

184. Rutagumba D, Hitayezu J, Kalimba E. Predictors of exclusive breastfeeding practice in urban kigali, rwanda – a cross-sectional study. *Rwanda Med J*. 2021;78(1):38-46.

185. Sonko A, Worku A. Prevalence and predictors of exclusive breastfeeding for the first six months of life among women in Halaba special woreda, Southern Nations, Nationalities and Peoples’ Region/SNNPR/, Ethiopia: a community based cross-sectional study. *Arch Public Health*. 2015;73:53. doi:10.1186/s13690-015-0098-4

186. Tewabe T, Mandesh A, Gualu T, Alem G, Mekuria G, Zeleke H. Exclusive breastfeeding practice and associated factors among mothers in Motta town, East Gojjam zone, Amhara Regional State, Ethiopia, 2015: a cross-sectional study. *Int Breastfeed J*. 2016;12(1):12. doi:10.1186/s13006-017-0103-3

187. Tewabe T, Mandesh A, Gualu T, Alem G, Mekuria G, Zeleke H. Exclusive breastfeeding practice and associated factors among mothers in Motta town, East Gojjam zone, Amhara Regional State, Ethiopia, 2015: A cross-sectional study. *Int Breastfeed J*. 2017;12(1). doi:10.1186/s13006-017-0103-3

188. Tsegaw SA, Ali Dawed Y, Tadesse Amsalu E. Exploring the determinants of exclusive breastfeeding among infants under-six months in Ethiopia using multilevel analysis. *PloS One*. 2021;16(1):e0245034. doi:10.1371/journal.pone.0245034

189. Ukwuani FA, Suchindran CM. Implications of women’s work for child nutritional status in sub-Saharan Africa: a case study of Nigeria. *Soc Sci Med*. 2003;56(10):2109-2121. doi:10.1016/s0277-9536(02)00205-8

190. Victor R, Baines SK, Agho KE, Dibley MJ. Determinants of breastfeeding indicators among children less than 24 months of age in Tanzania: a secondary analysis of the 2010 Tanzania Demographic and Health Survey. *BMJ Open*. 2013;3(1). doi:10.1136/bmjopen-2012-001529

191. Warille EB, Onyango FE, Osano B. Knowledge and practice of exclusive breastfeeding among women with children aged between 9 and 12 months in Al-Sabah Children Hospital, Juba, South Sudan. *South Sudan Med J*. 2017;10(1):12-16.

192. Woldeamanuel BT. Trends and factors associated to early initiation of breastfeeding, exclusive breastfeeding and duration of breastfeeding in Ethiopia: evidence from the Ethiopia Demographic and Health Survey 2016. *Int Breastfeed J*. 2020;15(1):3. doi:10.1186/s13006-019-0248-3

193. Yako EM, Nzama NPB. Maintenance of the selected infant feeding methods amongst postnatal mothers at risk of HIV in the Eastern Cape Province, South Africa. *Health SA Gesondheid*. 2013;18(1). doi:10.4102/hsag.v18i1.585

194. Yeboah JY, Forkuor D, Agyemang-Duah W. Exclusive breastfeeding practices and associated factors among lactating mothers of infants aged 6-24 months in the Kumasi Metropolis, Ghana. *BMC Res Notes*. 2019;12(1):689. doi:10.1186/s13104-019-4723-0

195. Yeheyis T, Berhanie E, Yihun M, Workineh Y. Timely Initiation of Complementary Feeding and Associated Factors among Children Aged 6 to 12 Months in Addis Ababa Ethiopia, 2015. *Epidemiol Open Access*. 2016;6(5). doi:10.4172/2161-1165.1000272

196. Yimer DS, Adem OS, Arefayene M, Chanie T, Endalifer ML. Exclusive breastfeeding practice and its associated factors among children aged 6-23 months in Woldia Town, Northwest Ethiopia. *Afr Health Sci*. 2021;21(4):1877-1886. doi:10.4314/ahs.v21i4.46

197. Nkrumah J, Abuosi AA, Nkrumah RB. Towards a comprehensive breastfeeding-friendly workplace environment: insight from selected healthcare facilities in the central region of Ghana. *BMC Public Health*. 2021;21(1):1647. doi:10.1186/s12889-021-11652-5

198. Okafor AE, Uche OA, Uche IB. Sociocultural Factors as Predictor to Exclusive Breastfeeding (EBF) Practice among Nursing Mothers in some Communities in Eastern, Nigeria. *Soc Work Public Health*. Published online 2022:1-13. doi:10.1080/19371918.2022.2135663

199. Yeneabat T, Belachew T, Haile M. Determinants of cessation of exclusive breastfeeding in Ankesha Guagusa Woreda, Awi Zone, Northwest Ethiopia: a cross-sectional study. *BMC Pregnancy Childbirth*. 2014;14(1):262. doi:10.1186/1471-2393-14-262

200. Wake GE, Mittiku YM. Prevalence of exclusive breastfeeding practice and its association with maternal employment in Ethiopia: a systematic review and meta-analysis. *Int Breastfeed J*. 2021;16(1):86. doi:10.1186/s13006-021-00432-x

201. Ejie IL, Eleje GU, Chibuzor MT, et al. A systematic review of qualitative research on barriers and facilitators to exclusive breastfeeding practice in sub-Saharan African countries. *Int Breastfeed J*. 2021;16(1):44. doi:10.1186/s13006-021-00380-6

202. Gyamfi A, Jefferson UT, O’Neill B, Lucas R, Spatz DL, Henderson WA. Disparities in 6 Month Exclusive Breastfeeding in Ghana, Africa: A Scoping Review. *J Hum Lact*. Published online 2022:8903344221130988. doi:10.1177/08903344221130988

203. Gebrekidan K, Fooladi E, Plummer V, Hall H. Enablers and barriers of exclusive breastfeeding among employed women in low and lower middle-income countries. *Sex Reprod Healthc*. 2020;25:100514. doi:10.1016/j.srhc.2020.100514

204. Horwood C, Surie A, Haskins L, et al. Attitudes and perceptions about breastfeeding among female and male informal workers in India and South Africa. *BMC Public Health*. 2020;20(1):875. doi:10.1186/s12889-020-09013-9

205. Mabaso BP, Jaga A, Doherty T. Family supportive supervision in context: supporting breastfeeding at work among teachers in South Africa. *Community Work Fam*. Published online 2022. doi:10.1080/13668803.2022.2049704

206. Maponya N, Janse van Rensburg Z, Du Plessis-Faurie A. Understanding South African mothers’ challenges to adhere to exclusive breastfeeding at the workplace: A qualitative study. *Int J Nurs Sci*. 2021;8(3):339-346. doi:10.1016/j.ijnss.2021.05.010

207. Stumbitz B, Jaga A. A Southern encounter: Maternal body work and low-income mothers in South Africa. *Gend Work Organ*. 2020;27(6):1485-1500. doi:10.1111/gwao.12527

208. Wolde FB, Ali JH, Mengistu YG. Employed mothers’ breastfeeding: Exploring breastfeeding experience of employed mothers in different work environments in Ethiopia. *PloS One*. 2021;16(11):e0259831. doi:10.1371/journal.pone.0259831

209. Afolayan AK, Olajumoke AA. Perception and Practices of Complementary Feeding among Infants’ Mothers in Southwestern Nigeria: A Qualitative Study. *J Nutr Food Secur*. Published online 2020. doi:10.18502/jnfs.v6i4.7616

210. Agyekum MW, Codjoe SNA, Dake FAA, Abu M. Enablers and inhibitors of exclusive breastfeeding: perspectives from mothers and health workers in Accra, Ghana. *Int Breastfeed J*. 2022;17(1):21. doi:10.1186/s13006-022-00462-z

211. Ahishakiye J, Bouwman L, Brouwer ID, Matsiko E, Armar-Klemesu M, Koelen M. Challenges and responses to infant and young child feeding in rural Rwanda: a qualitative study. *J Health Popul Nutr*. 2019;38(1):43. doi:10.1186/s41043-019-0207-z

212. Kimani-Murage EW, Wekesah F, Wanjohi M, et al. Factors affecting actualisation of the WHO breastfeeding recommendations in urban poor settings in Kenya. *Matern Child Nutr*. 2015;11(3):314-332. doi:10.1111/mcn.12161

213. Operto E. Knowledge, attitudes, and practices regarding exclusive breastfeeding among HIV-positive mothers in Uganda: A qualitative study. *Int J Health Plann Manage*. 2020;35(4):888-896. doi:10.1002/hpm.2966

214. Sewannonda A, Medel-Herrero A, Nankabirwa V, Flaherman VJ. Experiences and attitudes related to newborn feeding in central Uganda: A qualitative study. *PloS One*. 2022;17(10):e0274010. doi:10.1371/journal.pone.0274010

215. Wainaina CW, Wanjohi M, Wekesah F, Woolhead G, Kimani-Murage E. Exploring the Experiences of Middle Income Mothers in Practicing Exclusive Breastfeeding in Nairobi, Kenya. *Matern Child Health J*. 2018;22(4):608-616. doi:10.1007/s10995-018-2430-4
